# Synthesis of siRNAs containing carbocyclic nucleotides and the role of cyclopentane conformation in RNAi activity

**DOI:** 10.1039/d6cb00038j

**Published:** 2026-03-20

**Authors:** Jayanta Kundu, Dhrubajyoti Datta, Masaaki Akabane-Nakata, Soham Mandal, Monika Krampert, Martin Egli, Muthiah Manoharan

**Affiliations:** a Alnylam Pharmaceuticals 675 West Kendall Street Cambridge MA 02142 USA mmanoharan@alnylam.com; b Axolabs GmbH, Fritz-Hornschuch-Strasse 9 95326 Kulmbach Germany; c Department of Biochemistry, Vanderbilt University, School of Medicine Nashville TN 37232 USA

## Abstract

5′-(*E*)- and 5′-(*Z*)-vinylphosphonate carbocyclic DNA and 5′-(*E*)-vinylphosphonate 2′- and 3′-*O*-methyl carbocyclic RNAs were incorporated at 5′ termini of antisense strands of small interfering RNAs. All but the 3′-*O*-methyl carbocyclic analogue resulted in gene silencing activity better than the siRNA lacking a 5′ phosphate in cells and in mice.

## Abstract 2

5′-(*E*)- and 5′-(*Z*)-vinylphosphonate carbocyclic DNA and 5′-(*E*)-vinylphosphonate 2′- and 3′-*O*-methyl carbocyclic RNAs were incorporated at 5′ termini of antisense strands of small interfering RNAs.

## Graphical abstract

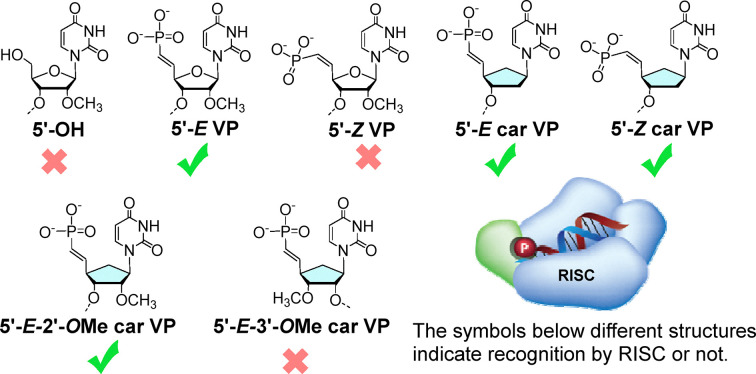

Chemical modification is necessary to ensure metabolic stability, specificity, and efficient delivery of small interfering RNAs (siRNAs).^1–3^ The RNA-induced silencing complex (RISC), which contains the endonuclease Ago2, mediates the gene silencing activities of siRNAs, and the reported high-resolution structures of these complexes have afforded insights into the mechanism of RNA interference (RNAi).^4–6^ In order to be loaded into the RISC, the antisense strand of the siRNA must be 5′ phosphorylated.^7^ When the antisense strand of a synthetic siRNA has a 5′-terminal residue that cannot be enzymatically phosphorylated, chemical incorporation of a natural 5′-monophosphate is ineffective due to rapid dephosphorylation by lysosomal acid phosphatases encountered by the siRNA during entry into cells *via* endocytosis.^8^

Incorporation of the metabolically stable phosphate mimic 5′-(*E*)-vinylphosphonate (5′-(*E*)-VP, I, Fig. 1) at the 5′ terminus of the antisense strand enhances RISC loading and siRNA potency, but the corresponding (*Z*) isomer (5′-(*Z*)-VP, II) does not.^8–12^ Crystal structures of Ago2 loaded with an antisense strand modified with 5′-(*E*)-VP (I) revealed that the 5′-nucleotide binding pocket, which involves residues of the MID and PIWI domains of Ago2, accommodates the 5′-(*E*)-VP (I) moiety but not the (*Z*) isomer (II).^5,13^ The combination of 5′-(*E*)-VP (I) in the antisense strand with targeting ligands in the sense strand such as triantennary *N*-acetylgalactosamine (GalNAc) for liver and 2′-*O*-hexadecyl lipid for central nervous system result in efficacious siRNAs.^14,15^ Though siRNAs with antisense strands modified with the 5′-(*Z*)-VP (II, Fig. 1) do not mediate gene silencing,^16^ siRNAs carrying 6′-(*E*)- and 6′-(*Z*)-VP (which are corresponding methylene homologues of 5′-(*E*)- and 5′-(*Z*)-VP, respectively) have comparable potency to siRNAs modified with 5′-(*E*)-VP (I) in mice.^17^ Gene silencing was more efficient when the antisense siRNA strand was modified with 5′-VP nucleosides that adopt a South C2′-*endo* pucker than with a 5′-VP nucleoside that adopts a North C3′-*endo* pucker.^18^

**Fig. 1:**
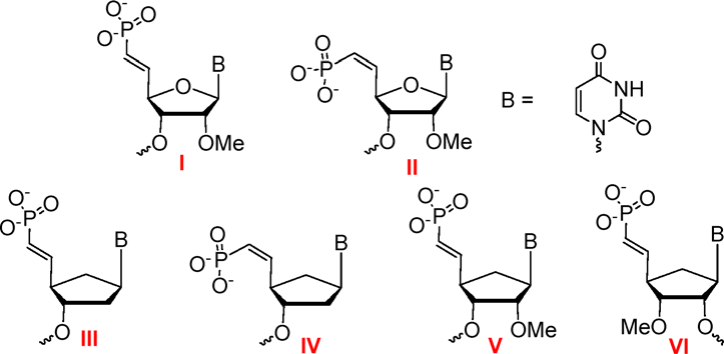
5′-(*E*)- and 5′-(*Z*)-VP-modified nucleotides previously tested in the context of siRNAs (I and II, respectively) and 5′-(*E*)- and 5′-(*Z*)-VP-modified nucleotides of car-DNA (III and IV, respectively) and 5′-(*E*)-VP-modified nucleotides of 2′- and 3′-*O*Me-car-RNA (V and VI, respectively) tested here.

In our effort to expand the toolbox of modifications for siRNAs, we previously evaluated biophysical properties of carbocyclic RNAs (car-RNAs).^19^ In this non-natural nucleic acid, the 4′-oxygen is replaced by a methylene group. Incorporation of a car-RNA residue does not alter the structure of an RNA duplex, and the 2′-OH group has higher p*K*_a_ and lower nucleophilicity than the ribose sugar, which explains their improved nuclease resistance.^20,21^ The (*E*)-VP analogue of car-RNA with a 2′-*O*-methyl (2′-OMe) sugar (V, Fig. 1) was mentioned in recently published patents,^22,23^ but the effect of this analogue on RNAi activity has not been reported. Here, we evaluated the effects of VP-modified carbocyclic DNA (car-DNA) and car-RNA residues (Fig. 1, III-VI) at the 5′ end of the antisense strand on siRNA potency in cell culture and in mice.

For the syntheses of VP-analogues of car-DNA nucleosides, we started from 2′-deoxycarbocyclic uridine nucleoside 1,^24,25^ which can be easily synthesized from the previously described car-U-RNA^19^ following a reported procedure.^26^ Compound 1 was reacted with excess *tert*-butyldimethylsilyl (TBS) chloride to afford 5′,3′-bis-OTBS product 2. Pyridinium *p*-toluenesulfonate-mediated selective removal of the 5′-OTBS yielded 3. Compound 3 was oxidized using 2-iodoxybenzoic acid to afford the aldehyde 4, which was used without further purification in a Wittig-type reaction in the presence of tetrakis[(pivaloyloxy)-methyl]methylenediphosphonate under basic conditions to afford a mixture of stereoisomers 5a and 5b. After 3′-OTBS removal under acidic conditions, an attempt to separate the *E* and *Z* isomers resulted in the pure 6a. However, 6b was obtained as an inseparable mixture with 10% 6a. Compound 6a and the partially pure 6b were then phosphitylated to afford the phosphoramidites 7a and 7b, respectively (Scheme 1).

**Scheme 1:**
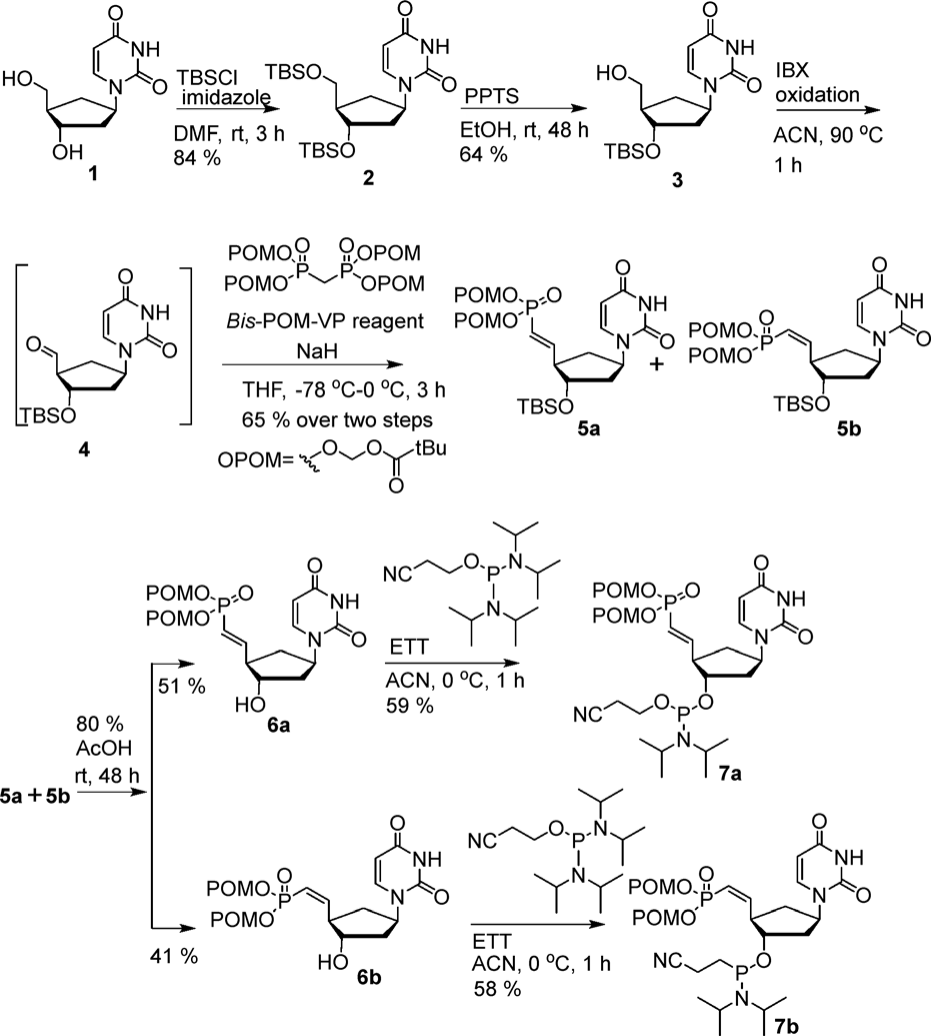
Syntheses of 5′-VP-car-DNA phosphoramidites 7a and 7b.

For the syntheses of VP analogues of 2′- and 3′-OMe-car-RNA, we converted 8, synthesized as described,^19^ into the corresponding organo-tin derivative 9 under Moffatt conditions.^27^ Compound 9 was separated into isomers 10 and 11 by a silylation–desilylation strategy *via*12 and 13. The corresponding VP analogues were synthesized as described in Scheme 1 to afford phosphoramidites 19 and 25 (Scheme 2).

**Scheme 2:**
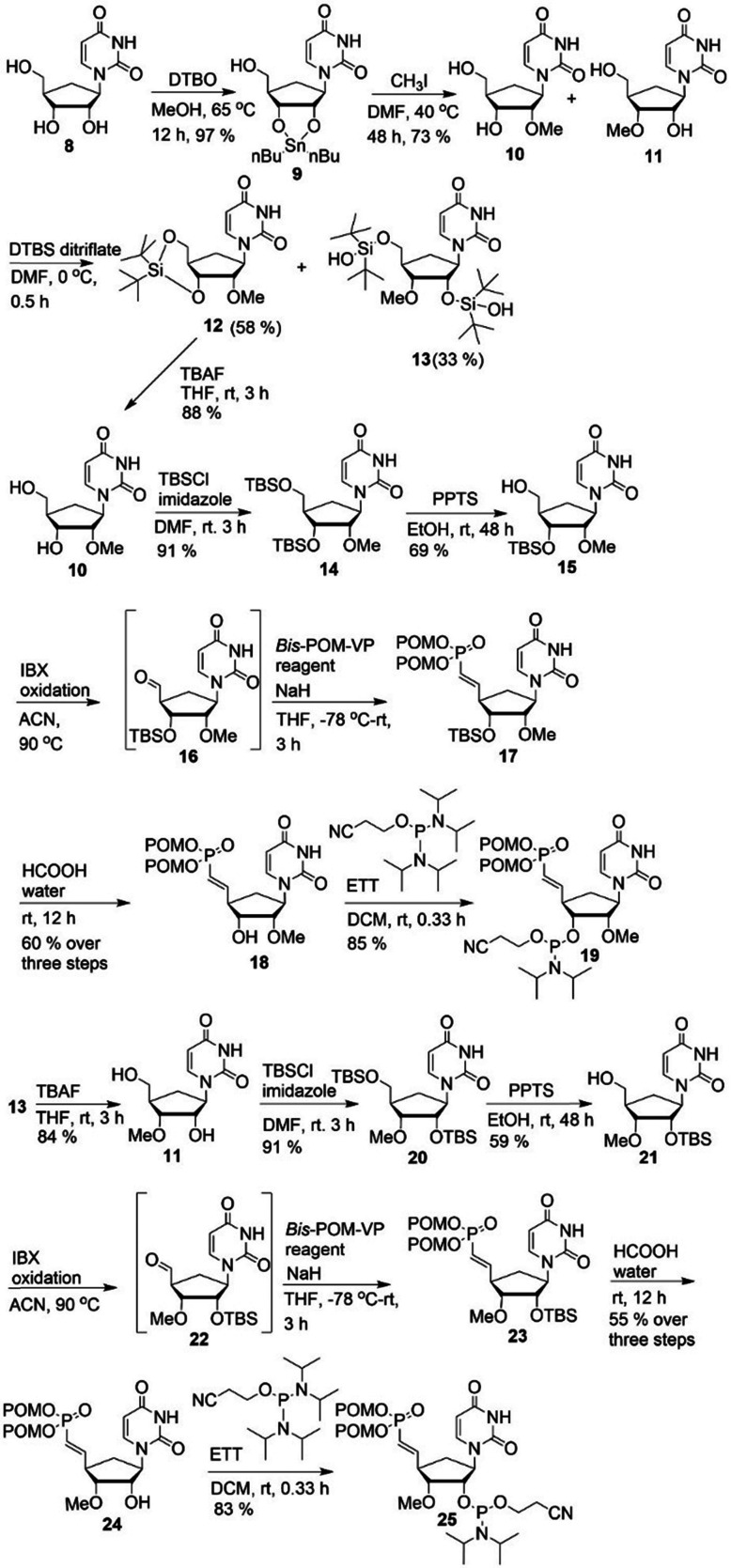
Syntheses of 5′-VP-2′-OMe-car-RNA and 5′-VP-3′-OMe-car-RNA phosphoramidites 19 and 25.

The phosphonate-protected phosphoramidites 7a**, 7**b, 19, and 25 were incorporated at the 5′ ends of antisense siRNA strands targeting mouse *Ttr* and *ApoB* mRNAs using standard automated solid-phase oligonucleotide synthesis procedures (Table 1 and Table S1). The 3′ termini of the sense strands were conjugated to triantennary GalNAc. Strands were chemically modified with 2′-fluoro-RNA, 2′-OMe-RNA, and phosphorothioate backbone linkages as previously described.^1,2^ Antisense strands that carried the novel car-DNA analogues were prepared with a phosphorothioate linkage and without. As controls, antisense strands without a 5′ phosphate and with a 5′-(*E*)-VP (I) were prepared. si-1 and si-2, without a 5′ phosphate and with a 5′-(*E*)-VP (I), respectively, were used as the controls for siRNAs targeting *Ttr*, and si-9 and si-10 served as controls for *ApoB*-targeting siRNAs.

**Table 1:** siRNAs used in this study

siRNA	5′ Antisense strand modification	Sense/antisense strand sequences (5′–3′)^a^
si-1	None	*A*•a•*C*a*G*u*G*u*UCU*u*G*c*U*c*U*a*U*a*A*L
		u•*U*•a*U*a*G*a*G*c*A*aga*A*c*A*c*U*g*U*u•u•u
si-2	5′-(*E)*-VP	*A*•a•*C*a*G*u*G*u*UCU*u*G*c*U*c*U*a*U*a*A*L
		**I**•*U*•a*U*a*G*a*G*c*A*aga*A*c*A*c*U*g*U*u•u•u
si-3	5′-(*E*)-VP-car-DNA	*A*•a•*C*a*G*u*G*u*UCU*u*G*c*U*c*U*a*U*a*A*L
		**III**•*U*•a*U*a*G*a*G*c*A*aga*A*c*A*c*U*g*U*u•u•u
si-4	5′-(*E*)-VP-car-DNA	*A*•a•*C*a*G*u*G*u*UCU*u*G*c*U*c*U*a*U*a*A*L
		**III** *U*•a*U*a*G*a*G*c*A*aga*A*c*A*c*U*g*U*u•u•u
si-5	5′-(*Z*)-VP-car-DNA	*A*•a•*C*a*G*u*G*u*UCU*u*G*c*U*c*U*a*U*a*A*L
		**IV**•*U*•a*U*a*G*a*G*c*A*aga*A*c*A*c*U*g*U*u•u•u
si-6	5′-(*Z*)-VP-car-DNA	*A*•a•*C*a*G*u*G*u*UCU*u*G*c*U*c*U*a*U*a*A*L
		**IV** *U*•a*U*a*G*a*G*c*A*aga*A*c*A*c*U*g*U*u•u•u
si-7	5′-(*E*)-VP-2′-OMe-car-RNA	*A*•a•*C*a*G*u*G*u*UCU*u*G*c*U*c*U*a*U*a*A*L
		**V**•*U*•a*U*a*G*a*G*c*A*aga*A*c*A*c*U*g*U*u•u•u
si-8	5′-(*E*)-VP-3′-OMe-car-RNA	*A*•a•*C*a*G*u*G*u*UCU*u*G*c*U*c*U*a*U*a*A*L
		**VI**•*U*•a*U*a*G*a*G*c*A*aga*A*c*A*c*U*g*U*u•u•u
si-9	None	*C*•c•*U*g*G*a*C*a*UUC*a*G*a*A*c*A*a*G*a*A*L
		u•*U*•c*U*u*G*u*U*c*U*gaa*U*g*U*c*C*a*G*g•g•u
si-10	5′-(*E*)-VP	*C*•c•*U*g*G*a*C*a*UUC*a*G*a*A*c*A*a*G*a*A*L
		**I**•*U*•c*U*u*G*u*U*c*U*gaa*U*g*U*c*C*a*G*g•g•u
si-11	5′-(*E*)-VP-car-DNA	*C*•c•*U*g*G*a*C*a*UUC*a*G*a*A*c*A*a*G*a*A*L
		**III**•*U*•c*U*u*G*u*U*c*U*gaa*U*g*U*c*C*a*G*g•g•u
si-12	5′-(*E*)-VP-2′-OMe-car-RNA	*C*•c•*U*g*G*a*C*a*UUC*a*G*a*A*c*A*a*G*a*A*L
		**V**•*U*•c*U*u*G*u*U*c*U*gaa*U*g*U*c*C*a*G*g•g•u
si-13	5′-(*E*)-VP-3′-OMe-car-RNA	*C*•c•*U*g*G*a*C*a*UUC*a*G*a*A*c*A*a*G*a*A*L
		**VI**•*U*•c*U*u*G*u*U*c*U*gaa*U*g*U*c*C*a*G*g•g•u

a Top and bottom rows show sense and antisense strand sequences, respectively. Upper case italics indicate 2′-fluoro RNA; lower case indicates 2′-OMe modification; I–VI are modifications shown in Fig. 1; L indicates triantennary GalNAc; and •indicates a phosphorothioate linkage.

The siRNAs were first evaluated in a gene silencing assay in primary mouse hepatocytes under free uptake conditions. At the lower doses tested, 1 nM for *Ttr*- and 20 nM for *ApoB*-targeted siRNAs, the controls carrying the 5′-(*E*)-VP (I; si-2 and si-10) were more potent than the controls lacking a 5′ phosphate analogue (si-1 and si-9) (Fig. 2A and C). The siRNAs modified with 5′-(*E*)-VP-2′-OMe-car-RNA (V; si-7 and si-12) and 5′-(*E*)-VP-car-DNA (III; si-3 and si-11) were similar in potency to control siRNAs modified with 5′-(*E*)-VP (I) (Fig. 2A and C). The siRNAs functionalized with 5′-(*Z*)-car-DNA (IV; si-5 and si-6) had potencies equivalent to that of si-2, the control modified with 5′-(*E)*-VP (I) (Fig. 2B). This was unexpected as a previous report demonstrated that siRNA functionalized with the 5′-(*Z*)-VP isomer II was not active at the concentrations evaluated here.^16^ There were no differences in potency between siRNAs modified with 5′-(*E*)-car-DNA (III) with and without phosphorothioate linkages between the first and second residues of the antisense strand (si-3*vs.*si-4, respectively) (Fig. 2B). This was expected as the car-DNA should enhance nuclease resistance.^21^

**Fig. 2:**
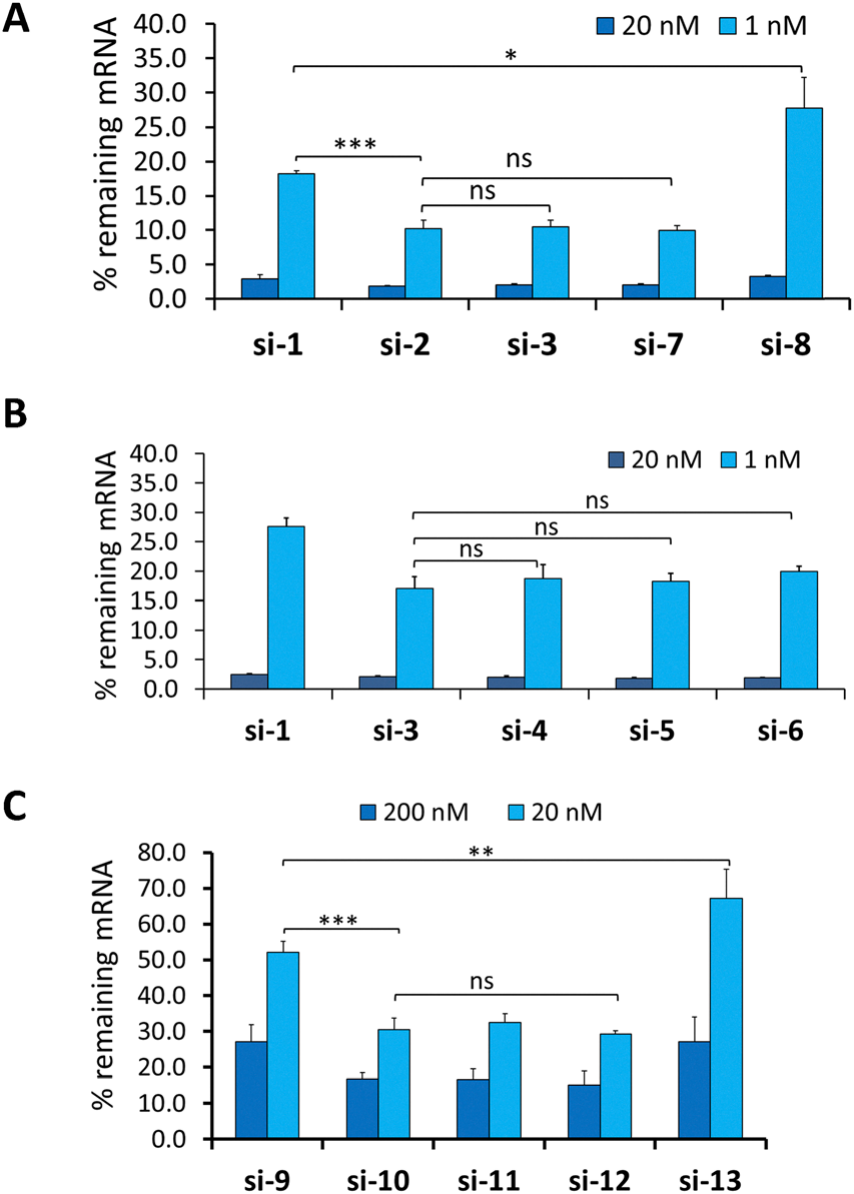
(A and B) Percent *Ttr* mRNA remaining in mouse hepatocytes after treatment with indicated siRNAs. Panels A and B show data from separate experiments. Primary mouse hepatocytes were cultured with siRNAs under free uptake conditions for 48 h. *Ttr* mRNA was quantified using a Quantigene Singleplex assay, and percent RNA remaining relative to samples treated with control, non-targeted siRNA was determined. Averages ± standard deviations are plotted (*n* = 3). *p < 0.05, ns means not significant; student's *t*-test was used. (C) Analysis of silencing by *ApoB*-targeting siRNAs in cultured primary mouse hepatocytes. Primary mouse hepatocytes were cultured with siRNAs under free uptake conditions for 48 h. *Apo* mRNA was quantified using a Quantigene Singleplex assay, and percent RNA remaining relative to samples treated with control, non-targeted siRNA was determined. Averages ± standard deviations are plotted (*n* = 3). *p < 0.05, ns means not significant; student's *t*-test was used.

The siRNAs modified with 3′-OMe-car-RNA (VI; si-8 and si-13), a modification with a 2′–5′ linkage, were even less potent than controls si-1 and si-9, which do not have a 5′ phosphate (Fig. 2A). siRNAs with 2′–5′ linkages have been evaluated in the past for RNAi activity. 2′–5′-linked DNA with a 5′-(*E)*-VP showed enhanced RNAi activity compared to the corresponding 5′-OH compound.^11^ On the other hand, 2′–5′-linked RNA, which has reduced immunostimulatory effects compared to RNA, showed nuclease resistance but reduced the Argonaute-2 loading when it was placed at the position 1 of the antisense strand even though the sugar ring had a favorable “clover leaf” bend and C2′-*endo* conformation.^29^ Moreover, multiple 2′-5′-linked RNA modifications in the antisense strand significantly reduced activity.^28,29^ However, a single 2′-5′-linked RNA modification at position 7 of the antisense strand seed region was recently shown to mitigate off-target effects arising from miRNA-type interactions with non-targeted mRNAs while maintaining the on-target activity.^30^

Next, we determined the potencies of selected *Ttr*-targeted siRNAs in mice. Mice were treated subcutaneously with a dose of 0.4 mg kg^−1^, and TTR protein was quantified in serum over time. si-3, which is modified with 5′-(*E*)-VP-car-DNA (III), and si-7, which is modified with 5′-(*E*)-VP-2′-OMe-car-RNA (V), had potencies equivalent to that of si-2, which carries 5′-(*E*)-VP (I), and were more efficacious than the non-phosphorylated control si-1 (Fig. 3). Consistent with the *in vitro* data, the siRNA with the 5′-(*E*)-VP-3′-OMe-car-RNA (VI) modification, si-8, was less potent than both control siRNAs (Fig. 3).

**Fig. 3:**
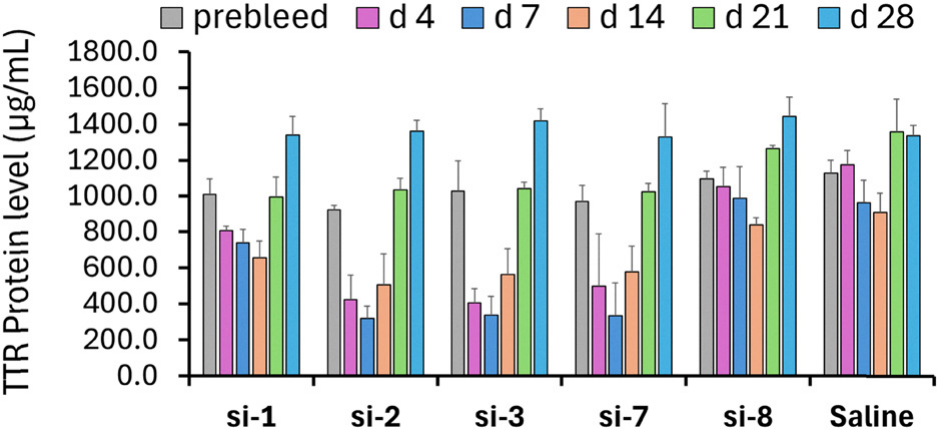
Levels of TTR protein in serum of mice dosed subcutaneously with 0.4 mg kg^−1^ indicated siRNA. TTR protein was quantified at the indicated days after dosing using an ELISA assay. Plotted are averages ± standard deviations normalized to pre-dose levels in individual animals (*n* = 3).

We used computational modelling to evaluate how modified carbocyclic 5′-VP analogues interact with the Ago2 MID domain. The complex between miR-20a, which has a 5′ UMP, served as the reference structure (PDB ID 4f3t).^4^ UCSF Chimera was used to install modified residues at the 5′ terminus of the RNA.^31^ All models were energy-minimized until conversion with the AMBER ff14 force field as implemented in UCSF Chimera.^32^ The sugar of 5′-(*E*)-VP-car-DNA (III) is accommodated within the MID domain binding site and adopts the C2′-*endo* pucker (Fig. 4A). The sugar of the 5′-(*Z*)-VP-car-DNA (IV) adopts the C3′-*exo* pucker (Fig. 4B). The conformations of both these residues are very similar to the conformation of the 5′ UMP in the crystal structure. The 5′-(*E*)-VP-2′-OMe-car-RNA (V) also adopts the C2′-*endo* sugar pucker (Fig. 5A). The conformations of these 5′-VP residues also closely correspond to that of the 5′-(*E*)-VP-2′-OMe uracil in the complex of a modified strand bound to Ago2, which was previously analysed by crystallography (PDB ID 5t7b), although in that structure, the pucker is C1′-*exo* (Δ*P* = 50°).^33^ In the crystal structures and our models, the uracil stacks favourably with Y529 Ago2. The siRNA modified with 5′-(*E*)-VP-3′-OMe-car-RNA (VI) was less active than the other modified siRNAs tested, and VI is not well accommodated in the MID domain binding site; there are short contacts to phosphates (sum vdW = 2.8 + 2 = 4.8) and a short O3′ distance of 2.7 Å to C5′ of the second nucleotide in the antisense strand (Fig. 5B). Moreover, neither the VP moiety nor the base are planar, and the sugar pucker of the carbocyclic ring is C1′-*exo* (Southeast). The interaction with Y529 Ago2 is also disrupted in the 5′-(*E*)-VP-3′-OMe-car-RNA (VI) model, although the uracil base does form a hydrogen bond to the main chain of the local Ago2 loop (*via* N3H).

**Fig. 4:**
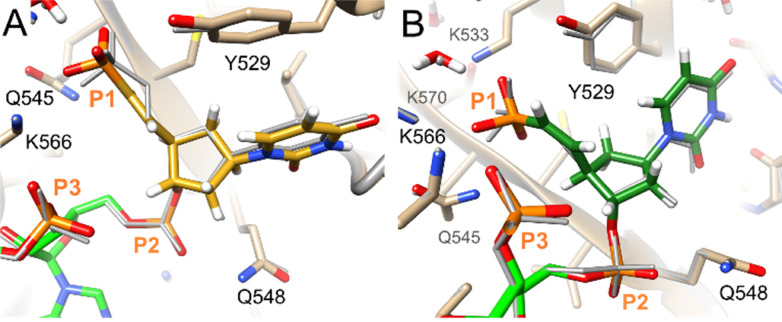
Models of antisense strands modified with (A) 5′-(*E*)-VP-car-DNA III in golden and (B) 5′-(*Z*)-VP-car-DNA IV in dark green lodged at the Ago2 MID domain binding site with the RNA in the crystal structure of the miR-20a and Ago2 complex (PDB ID 4f3t) shown as a grey wire.

**Fig. 5:**
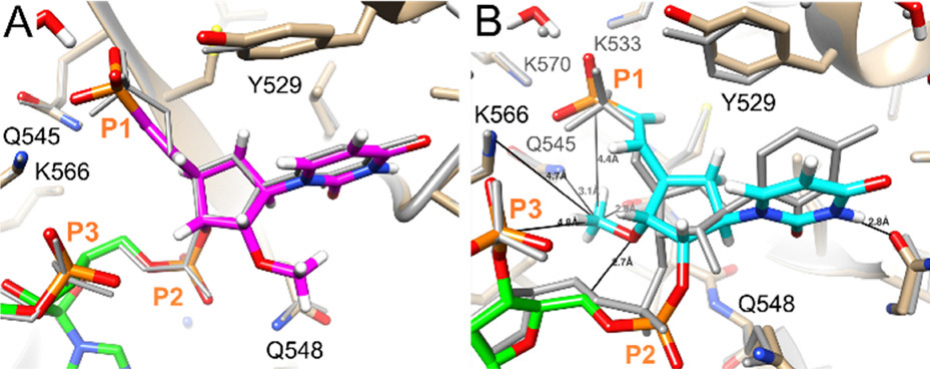
Models of antisense strands modified with (A) 5′-(*E*)-VP-2′-OMe-car-RNA V in pink and (B) 5′-(*E*)-VP-3′-OMe-car-RNA VI in cyan lodged at the Ago2 MID domain binding site. The RNA in the crystal structure (PDB ID 4f3t) is shown as a grey wire.

In summary, we report the syntheses of four carbocyclic phosphoramidites and their incorporation at the 5′ termini of antisense strands of siRNAs. Modification of siRNA with either isomer of 5′-VP-car-DNA (III or IV) or with 5′-(*E)*-2′-OMe-car-RNA (V) resulted in siRNAs with potencies comparable to the siRNA with an antisense strand modified with 5′-(*E*)-VP (I) and more active than the siRNA with an antisense strand lacking a 5′ phosphate. The 5′-(*E*)-3′-OMe-car-RNA (VI) analogue was less active than the siRNA with an antisense strand lacking a 5′ phosphate. Interestingly, the siRNA modified with 5′-(*Z*)-VP-car-DNA (IV) was as potent as the siRNA modified with the 5′-(*E*) analogue (III). The former was not tested in mice due to poor yields. The activity of the siRNA modified with the (*Z*) isomer was unexpected as siRNAs with an antisense strand carrying the 5′-(*Z*)-VP (II) moiety on a 2′-OMe sugar are not active at the concentrations tested here.^5,13^ Molecular modelling studies showed that the car-DNAs III and IV as well as 5′-VP-2′-OMe-car-RNA (V) fit well inside the Ago2 MID domain binding pocket. However, the 5′-(*E*)-VP-3′-OMe-car-RNA (VI) has steric clashes that stem from the 3′-OMe group even though the sugar pucker of the carbocyclic ring is C1′-*exo*. Given the high metabolic stability of car-RNA and car-DNA analogs^21^ and the unexpected silencing activity of 5′-(*Z*)-VP-car-DNA (IV), these new VP analogues should prove useful in development of more efficacious RNAi therapeutics.

## Conflicts of interest

There are no conflicts to declare.

## Live subject statement

All animal studies were conducted following the animal welfare regulations of the state of Bavaria (Germany) and the European Union (guideline 2010/63/EU). Protocols were approved by the government of lower Franconia (Approval Nr. 55.2.2-2532-2-1548-20). This has been included as part of the SI.

## Supplementary Material

CB-OLF-D6CB00038J-s001

## Data Availability

The data supporting this article has been included as part of the supplementary information (SI). Supplementary information: Synthesis of building blocks and oligonucleotide characterization are available in the published version. See DOI: https://doi.org/10.1039/d6cb00038j.
