# Supplementary material for: Synthesis of siRNAs containing carbocyclic nucleotides and the role of cyclopentane conformation in RNAi activity
Source: RSC Chem Biol. 2026 Mar 20;7(4):590–5. doi: 10.1039/d6cb00038j (PMC13003969; doi:10.1039/d6cb00038j)
Supplement: CB-007-D6CB00038J-s001 [file CB-007-D6CB00038J-s001.pdf]

This journal is © The Royal Society of Chemistry 2026

SUPPLEMENTARY INFORMATION

Synthesis of siRNAs containing carbocyclic nucleotides and the role of cyclopentane conformation in RNAi activity

Jayanta Kundu[a], Dhrubajyoti Datta[a], Masaaki Akabane-Nakata[a], Soham Mandal[b], Monika Krampert[b], Martin Egli[c], and Muthiah Manoharan[a]*

[a]*Alnylam Pharmaceuticals, 675 West Kendall, Cambridge, MA 02142, USA*
[b]*Axolabs GmbH, Fritz-Hornschuch-Strasse 9, 95326 Kulmbach, Germany*
[c]*Department of Biochemistry, Vanderbilt University, School of Medicine Nashville, TN 37232, USA*

Correspondence to: mmanoharan@alnylam.com

Table of Contents

Synthetic procedures and compound characterization………………………………………………………..	**S-2**
Oligonucleotide synthesis, purification, and characterization………………………………………………	**S-13**
Table S1: Analytical data for sense and antisense strands……………………………………………………	**S-14**
Analyses of siRNA activity in cultured primary mouse hepatocytes…………………………………….	**S-15**
Analyses of siRNA activity in mice………………………………………………………………………………………	**S-15**
^{1}H, ^{13}C and ^{31}P NMR spectra of new compounds………………………………………………………………..	**S-16**
HPLC profiles of oligonucleotides……………………………………………………………………………………….	**S-64**
References…….	**S-79**
.	

Synthetic procedures and compound characterization

General conditions: TLC was performed on Merck silica gel 60 plates coated with F254. Compounds were visualized under UV light (254 nm) or after spraying with the *p*-anisaldehyde staining solution followed by heating. Flash column chromatography was performed using a Teledyne ISCO Combi Flash system with pre-packed RediSep Teledyne ISCO silica gel cartridges. All moisture-sensitive reactions were carried out under anhydrous conditions using dry glassware, anhydrous solvents, and argon atmosphere. All commercially available reagents and solvents were purchased from Sigma-Aldrich unless otherwise stated and were used as received. ESI-MS spectra were recorded on a Waters Q-Tof Premier instrument using the direct flow injection mode. ^{1}H NMR spectra were recorded at 600 MHz. ^{13}C NMR spectra were recorded at 151 MHz. ^{31}P NMR spectra were recorded at 243 MHz. Chemical shifts are given in ppm referenced to the solvent residual peak (DMSO-d_6 – ^{1}H δ at 2.50 ppm and ^{13}C δ at 39.5 ppm; $CDCl_3$ – ^{1}H δ at 7.26 ppm and ^{13}C δ at 77.2 ppm; ACN-d_3 – ^{1}H δ at 1.96 ppm and ^{13}C δ at 1.8 and 118.3 ppm; D_2O – ^{1}H δ 4.87 ppm). Coupling constants are given in Hertz (Hz). Signal splitting patterns are described as singlet (s), doublet (d), triplet (t), dd (doublet of doublets), dt (doublet of triplets), td (triplet of doublets), tt (triplet of triplets) ddd (doublet of doublets of doublets), dtd (doublet of triplets of doublets), ddt (doublet of doublets of triplets), p (pentet), qt (quintet of triplets), dp (doublet of pentets), dq (doublet of quartets), dddd (doublet of doublets of doublets of doublets), or multiplet (m).

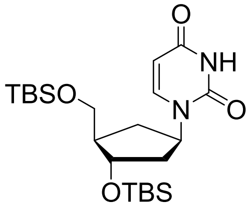

2

1-((1R,3S,4R)-3-((tert-butyldimethylsilyl)oxy)-4-(((tert-butyldimethylsilyl)oxy)methyl)cyclopentyl)pyrimidine-2,4(1H,3H)-dione **2**: To a solution of **1** (2 g, 8.84 mmol) and imidazole (1.81 g, 26.5 mmol) in DMF (100 mL) was added *tert*-butyldimethylsilyl (TBS) chloride (2.80 g, 18.6 mmol), and the mixture was stirred at room temperature for 3 h. The reaction was quenched with saturated $NaHCO_3$ (aq.) and diluted with diethyl ether. The organic layer was washed with water and brine, dried (Na_2SO_4), and concentrated under vacuum. The crude residue was purified by column chromatography on silica gel (0–40% EtOAc in hexanes) to afford **2** (3.38 g, 84% yield) as a white foam. ^{1}H NMR (600 MHz, DMSO-d_6) δ 11.21 (d, *J* = 2.4 Hz, 1H), 7.63 (d, *J* = 8.0 Hz, 1H), 5.56 (dd, *J* = 8.0, 2.3 Hz, 1H), 4.91 (p, *J* = 8.9 Hz, 1H), 4.18 (d, *J* = 5.6 Hz, 1H), 3.64 – 3.52 (m, 2H), 2.10 (dt, *J* = 12.7, 7.9 Hz, 1H), 2.05 – 1.91 (m, 2H), 1.78 (ddd, *J* = 12.6, 8.2, 3.8 Hz, 1H), 1.35 (dt, *J* = 12.6, 9.6 Hz, 1H), 0.86 (d, *J* = 9.9 Hz, 18H), 0.03 (d, *J* = 5.7 Hz, 12H) ppm. ^{13}C NMR (126 MHz, DMSO-d_6) δ 163.1, 150.9, 142.4, 101.4, 72.5, 63.4, 53.8, 49.2, 38.6, 31.4, 25.8, 25.7, 18.0, 17.7, -4.7, -4.9, -5.5, -5.5 ppm. HRMS calc. for $C_{22}H_{43}N_2O_4Si_2$ [M+H]$^{+}$: 455.2761; found: 455.2739.

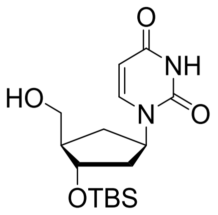

3

1-((1R,3S,4R)-3-((tert-butyldimethylsilyl)oxy)-4-(hydroxymethyl)cyclopentyl)pyrimidine-2,4 (1H,3H)-dione **3**: To a solution of **2** (1 g, 2.20 mmol) in EtOH (20 mL) was added pyridinium *p*-toluene sulfonate (138.15 mg, 549.75 µmol), and the mixture was stirred at room temperature for 48 h. The reaction mixture was concentrated under vacuum. The crude residue was purified by column chromatography on silica gel (0–10% MeOH in CH_2Cl_2) to afford **3** (480 mg, 64% yield) and **2** (127 mg, 26 %) both as white solids. Starting material **6** (113 mg) was also recovered. ^{1}H NMR (600 MHz, DMSO-d_6) δ 11.21 (s, 1H), 7.68 (d, J = 8.0 Hz, 1H), 5.57 (d, J = 8.0 Hz, 1H), 4.99 – 4.88 (m, 1H), 4.65 (t, J = 5.1 Hz, 1H), 4.22 – 4.13 (m, 1H), 3.46 (dt, J = 10.6, 5.4 Hz, 1H), 3.38 (dt, J = 10.8, 5.6 Hz, 1H), 2.09 (dt, J = 12.6, 7.8 Hz, 1H), 1.96 (dtd, J = 17.9, 9.0, 6.1 Hz, 1H), 1.91 (dt, J = 8.2, 4.2 Hz, 1H), 1.77 (ddd, J = 12.4, 8.1, 3.5 Hz, 1H), 1.38 (dt, J = 12.5, 9.6 Hz, 1H), 0.85 (s, 9H), 0.04 (d, J = 5.9 Hz, 6H) ppm. ^{13}C NMR (151 MHz, DMSO-d_6) δ 163.2, 151.0, 142.4, 101.4, 73.0, 62.1, 53.6, 49.4, 31.9, 25.8, 17.7, -4.7, -4.8 ppm. HRMS calc. for $C_{16}H_{29}N_2O_4Si$ $[M+H]^+$: 341.1897; found: 341.1899.

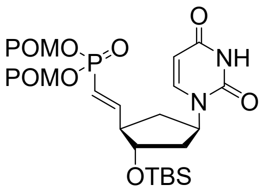

5a

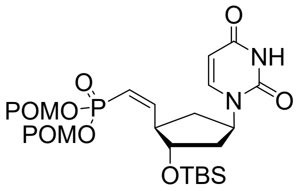

5b

[[(*E*)-2-[(1R,4R)-2-[tert-butyl(dimethyl)silyl]oxy-4-(2,4-dioxopyrimidin-1-yl)cyclopentyl]vinyl]-(2,2-dimethylpropanoyloxymethoxy)phosphoryl]oxymethyl 2,2-dimethylpropanoate 5a and [[(Z)-2-[(1R,4R)-2-[tert-butyl(dimethyl)silyl]oxy-4-(2,4-dioxopyrimidin-1-yl)cyclopentyl]vinyl]-(2,2-dimethylpropanoyloxymethoxy)phosphoryl]oxymethyl 2,2-dimethylpropanoate **5b**: To a solution of **3** (800 mg, 2.35 mmol) in CH_3CN (25 mL) was added 2-iodoxybenzoic acid (45 wt %; 2.49 g, 3.99 mmol), and the mixture was stirred at 90 °C for 1 h. The reaction mixture was filtered through a silica gel pad, and the filtrate was concentrated under vacuum. The crude residue **4** was used for the next reaction without further purification. To a suspension of sodium hydride (60% dispersion in mineral oil; 112.77 mg, 4.70 mmol) in THF (15 ml) was added dropwise a solution of tetra(pivaloyloxymethyl)-bis-phosphonate (2.97 g, 4.70 mmol) at -78 °C, and the mixture was stirred at 0° C for 30 min. To the suspension was added dropwise a solution of the crude compound in THF (10 mL), and the mixture was stirred at room temperature for 3 h. The reaction mixture was quenched with saturated NH_4Cl (aq.) and extracted with EtOAc. The organic layer was washed with water and brine, dried (Na_2SO_4), and concentrated under vacuum. The crude residue was purified by column chromatography on silica gel (60–100% EtOAc in hexanes) to afford a mixture of **5a** and **5b** (982 mg, 65% yield) as a white foam. ^{1}H NMR (600 MHz, DMSO-d_6) δ 11.23 (d, J = 2.1 Hz, 1H), 7.73 (d, J = 8.0 Hz, 1H), 6.80 – 6.70 (m, 1H), 5.95 – 5.83 (m, 1H), 5.64 – 5.53 (m, 5H), 4.92 (ddd, J = 17.4, 10.0, 7.2 Hz, 1H), 4.28 (q, J = 7.4 Hz, 1H), 3.32 (s, 1H), 2.58 (dq, J = 14.6, 7.6 Hz, 1H), 2.08 (ddt, J = 14.1, 11.4, 7.5 Hz, 2H), 1.89 (ddd, J = 13.5, 10.3,

6.5 Hz, 1H), 1.74 (td, J = 12.1, 10.2 Hz, 1H), 1.16 (d, J = 4.9 Hz, 18H), 0.84 (s, 9H), 0.01 (s, 6H) ppm. ^{13}C NMR (151 MHz, DMSO-d_6) δ 176.0, 176.0, 163.2, 154.4, 154.3, 150.8, 143.1, 117.7, 116.4, 101.5, 81.4, 81.4, 81.3, 81.3, 81.2, 81.2, 81.2, 76.4, 75.4, 53.8, 53.3, 51.5, 51.4, 26.5, 25.7, 17.7, -4.7, -4.9 ppm. ^{31}P NMR (243 MHz, DMSO-d_6) δ 17.83, 16.20 ppm. HRMS calc. for $C_{29}H_{49}N_2O_{10}PSiNa$ $[M+Na]^+$: 667.2792; found: 667.2761.

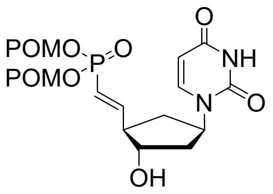

6a

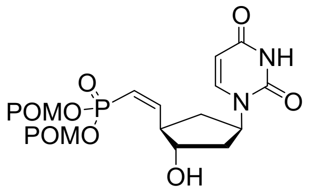

6b

((((*E*)-2-((1R,2S,4R)-4-(2,4-dioxo-3,4-dihydropyrimidin-1(2H)-yl)-2-hydroxycyclopentyl)vinyl) phosphoryl)bis(oxy))bis(methylene) bis(2,2-dimethylpropanoate) **6a**: The mixture of **5a** and **5b** (600 mg, 930.57 µmol) was dissolved in 80% aqueous acetic acid and the mixture was stirred at room temperature for 48 h. The reaction mixture was partitioned between EtOAc and water. The organic layer was washed with water, dried over Na_2SO_4, and concentrated under vacuum. The crude residue was purified by column chromatography on silica gel (50–100% EtOAc in hexanes) to afford (*E*) isomer **6a** (252 mg, 51%) and a mixture of **6a (**10 %**)** and (*Z*) isomer **6b** (200 mg, 41%) both as white foams.

Data for **6a**: ^{1}H NMR (600 MHz, DMSO-d_6) δ 11.23 (d, J = 2.2 Hz, 1H), 7.71 (d, J = 8.0 Hz, 1H), 6.88 – 6.78 (m, 1H), 5.94 – 5.82 (m, 1H), 5.59 (dd, J = 13.5, 1.9 Hz, 5H), 5.14 (d, J = 5.4 Hz, 1H), 4.95 – 4.89 (m, 1H), 4.06 (ddd, J = 13.1, 7.5, 5.7 Hz, 1H), 3.33 (s, 1H), 2.54 (dd, J = 11.5, 6.7 Hz, 1H), 2.09 (dt, J = 13.6, 7.3 Hz, 1H), 2.02 (dt, J = 13.5, 7.9 Hz, 1H), 1.88 (ddt, J = 14.2, 9.6, 5.6 Hz, 1H), 1.69 (q, J = 11.4 Hz, 1H), 1.16 (d, J = 3.5 Hz, 18H) ppm. ^{13}C NMR (151 MHz, DMSO-d_6) δ 176.1, 176.1, 163.2, 155.1, 155.0, 150.9, 142.8, 116.8, 115.5, 101.5, 81.4, 81.4, 81.4, 81.4, 73.8, 53.2, 51.0, 50.9, 26.5 ppm. ^{31}P NMR (243 MHz, DMSO-d_6) δ 18.48 ppm. HRMS calc. for $C_{23}H_{35}N_2O_{10}PNa$ $[M+Na]^+$: 553.1927; found: 553.1918.

Data for **6b**: ^{1}H NMR (DMSO-d_6) δ 11.23 (d, J = 2.2 Hz, 1H), 7.71 (d, J = 8.0 Hz, 1H), 6.70 – 6.54 (m, 1H), 5.77 – 5.68 (m, 1H), 5.65 – 5.56 (m, 5H), 4.99 (d, J = 5.4 Hz, 1H), 4.93 (tt, J = 9.9, 7.8 Hz, 1H), 4.08 (p, J = 6.0 Hz, 1H), 3.33 (s, 5H), 2.15 – 2.02 (m, 2H), 1.87 (ddd, J = 14.2, 9.6, 5.6 Hz, 1H), 1.59 (dt, J = 12.4, 10.5 Hz, 1H), 1.16 (d, J = 11.5 Hz, 18H) ppm. ^{13}C NMR (151 MHz, DMSO-d_6) δ 176.1, 176.0, 163.2, 156.7, 156.7, 150.9, 142.8, 116.5, 115.2, 101.5, 81.4, 81.4, 81.3, 81.3, 74.9, 53.6, 47.7, 47.6, 26.5, 26.45. ^{31}P NMR (243 MHz, DMSO-d_6) δ 16.65. HRMS calc. for $C_{23}H_{36}N_2O_{10}P$ $[M+H]^+$: 531.2108; found: 531.2100.

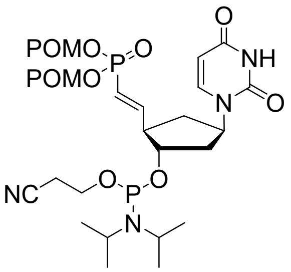

7a

[[(*E*)-2-[(1R,4R)-2-[2-cyanoethoxy-(diisopropylamino)phosphanyl]oxy-4-(2,4-dioxopyrimidin-1-yl)cyclopentyl]vinyl]-(2,2-dimethylpropanoyloxymethoxy)phosphoryl]oxymethyl-2,2-dimethylpropanoate **7a**: To a solution of **6a** (750 mg, 1.41 mmol) and 5-ethylthio-1*H*-tetrazole (0.25 M in CH_3CN; 5.6 mL, 1.41 mmol) in CH_2Cl_2 (8 mL) was added dropwise 2-cyanoethyl-*N*,*N*-diisopropylchlorophosphoramidite (0.449 mL, 1.41 mmol) at 0 °C, and the mixture was stirred at room temperature for 1 h. The reaction mixture was diluted with EtOAc and washed with saturated $NaHCO_3$ (aq.), water, and brine, dried (Na_2SO_4), and concentrated under vacuum. The crude residue was purified by column chromatography on silica gel (50–80% EtOAc in hexanes) to afford **7a** (605 mg, 59%) as a white foam. ^1H NMR (600 MHz, CD_3CN) δ 9.21 (s, 1H), 7.37 (dd, *J* = 8.1, 5.3 Hz, 1H), 6.90 – 6.77 (m, 1H), 5.94 – 5.78 (m, 1H), 5.67 – 5.55 (m, 5H), 4.93 (qt, *J* = 9.8, 7.5 Hz, 1H), 4.45 – 4.26 (m, 1H), 3.84 – 3.69 (m, 2H), 3.59 (dp, *J* = 10.2, 6.8 Hz, 2H), 2.89 – 2.73 (m, 1H), 2.72 – 2.61 (m, 2H), 2.28 – 2.08 (m, 4H), 1.75 (dddd, *J* = 12.8, 11.3, 10.1, 8.5 Hz, 1H), 1.28 – 1.10 (m, 31H) ppm. ^{13}C NMR (151 MHz, CD_3CN) δ 177.5, 164.2, 164.2, 155.0, 155.0, 154.7, 154.7, 151.8, 151.8, 143.7, 143.7, 118.3, 102.5, 102.5, 82.6, 82.6, 82.6, 82.5, 82.5, 77.8, 77.7, 77.3, 77.2, 59.7, 59.6, 59.3, 59.2, 55.5, 55.4, 51.7, 51.7, 51.6, 51.6, 51.4, 51.3, 51.2, 51.2, 44.0, 43.9, 39.4, 38.8, 38.8, 38.6, 38.6, 34.9, 34.9, 27.1, 25.0, 25.0, 24.9, 24.9, 24.8, 24.8, 21.0, 21.0, 21.0, 21.0 ppm. ^{31}P NMR (243 MHz, CD_3CN) δ 147.58, 147.54, 17.59 ppm. HRMS calc. for $C_{32}H_{53}N_4O_{11}P_2$ $[M+H]^+$: 731.3186; found: 731.3181.

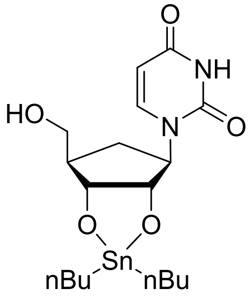

9

1-[2,2-di(butyl)-6-(oxidanylmethyl)-3a,4,6,6a-tetratritio-5H-cyclopenta[d][1,3,2]dioxastannol-4-yl]pyrimidine-2,4-dione **9**: To a suspension of **8** (2 g, 8.26 mmol) in dry MeOH (100 mL) under nitrogen atmosphere was added dibutyltin oxide (2.06 g, 8.26 mmol). The resulting mixture was allowed to reflux for 12 h at 65 °C. After evaporation to dryness, the residue was co-evaporated with DCM five times and dried under vacuum to afford **9** (3.79 g, 97% yield) as a yellowish white solid. The compound was used for next step without further purification. ^1H NMR (600 MHz, DMSO-d_6) δ 11.24 (s, 1H), 7.75 (d, *J* = 8.1 Hz, 1H), 5.60 (d, *J* = 8.0 Hz, 1H), 4.79 (s, 1H), 4.41 (d, *J* = 9.5 Hz, 1H), 4.21 (d, *J* = 7.6 Hz, 1H), 3.70 (dd, *J* = 6.2, 2.8 Hz, 1H), 3.51 – 3.37 (m, 2H), 2.07 – 1.82 (m, 2H), 1.67 – 1.41 (m, 5H), 1.34 – 1.17 (m, 8H), 0.86 (t, *J* = 7.3 Hz, 6H) ppm. ^{13}C

NMR (151 MHz, DMSO-d_6) δ 163.6, 151.9, 142.6, 101.7, 76.4, 75.4, 63.9, 61.8, 55.4, 49.1, 46.4, 29.6, 26.8, 14.0 ppm. HRMS calc. for $C_{18}H_{31}N_2O_5Si$ $[M+H]^+$: 383.2002; found: 383.2007.

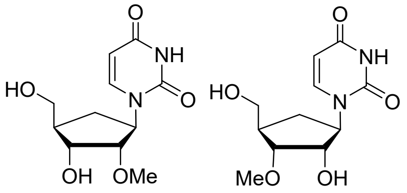

10 **11**

1-[2-methoxy-3-oxidanyl-4-(oxidanylmethyl)-1,2,3,4-tetratritio-cyclopentyl]pyrimidine-2,4-dione 10 and 1-[3-methoxy-2-oxidanyl-4-(oxidanylmethyl)-1,2,3,4-tetratritio-cyclopentyl]pyrimidine-2,4-dione **11**: To a clear solution of **9** (3.81 g, 8.05 mmol) in DMF (30 mL) was added CH_3I (34.29 g, 241.57 mmol, 15.04 mL), and the reaction mixture was heated at 40 °C for 48 h. The reaction mixture was dried and purified by silica gel column chromatography (5-20% MeOH in DCM) to afford **10** and **11** as a mixture (1.5 g total, 73% yield), which was used for next step. ^1H NMR (600 MHz, DMSO-d_6) δ 11.24 (s, 1H), 11.21 (s, 1H), 7.71 (d, *J* = 8.0 Hz, 1H), 7.67 (d, *J* = 8.0 Hz, 1H), 5.59 (dd, *J* = 10.8, 7.9 Hz, 2H), 4.89 (d, *J* = 6.9 Hz, 1H), 4.79 – 4.73 (m, 2H), 4.72 (t, *J* = 5.2 Hz, 1H), 4.62 (d, *J* = 4.8 Hz, 1H), 4.61 – 4.56 (m, 1H), 4.12 – 4.05 (m, 1H), 3.94 (td, *J* = 4.8, 2.6 Hz, 1H), 3.72 (dd, *J* = 9.5, 4.9 Hz, 1H), 3.38 (dt, *J* = 11.3, 5.0 Hz, 3H), 3.32 (s, 1H), 3.31 (s, 2H), 3.25 (s, 3H), 2.07 – 1.91 (m, 4H), 1.29 – 1.18 (m, 2H) ppm. ^{13}C NMR (151 MHz, DMSO-d_6) δ 163.1, 151.4, 151.2, 142.6, 142.3, 101.5, 101.2, 82.5, 81.3, 72.9, 68.9, 62.8, 62.8, 59.9, 58.0, 56.9, 56.8, 54.9, 45.0, 42.0, 27.6, 27.3 ppm. HRMS calc. for $C_{11}H_{16}N_2O_5Na$ $[M+Na]^+$: 279.0957; found: 279.0962.

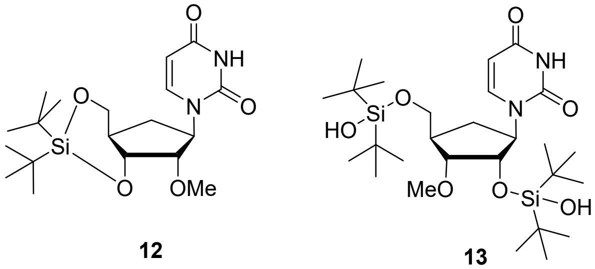

12 **13**

1-((4aR,6R,7S,7aR)-2,2-di-tert-butyl-7-methoxyhexahydrocyclopenta[d][1,3,2]dioxasilin-6-yl)pyrimidine-2,4(1H,3H)-dione 12 and [[3-[bis[1,1-di(methyl)ethyl]-[tris(fluoranyl)methylsulfonyloxy]silyl]oxy-4-[2,4-bis(oxidanylidene)pyrimidin-1-yl]-2-methoxy-1,2,3,4-tetratritio-cyclopentyl]methoxy-bis[1,1-di(methyl)ethyl]silyl] tris(fluoranyl)methanesulfonate **13**: To a clear solution of the mixture of **10** and **11** **(**833 mg, 3.25 mmol) in DMF (20 mL) was added [bis[1,1-di(methyl)ethyl]-[tris(fluoranyl) methylsulfonyloxy]silyl]tris(fluoranyl) methanesulfonate (2.58 g, 5.85 mmol, 1.91 mL) at 0 °C, and the reaction mixture was allowed to stir at room temperature for 30 min. TLC showed complete consumption of starting material. The reaction mixture was diluted with EtOAc, washed with water and brine, dried over Na_2SO_4, and filtered. The organic layer was concentrated under vacuum, and the crude product was purified by silica gel column chromatography (30-50 % EtOAc in hexanes) to afford **12** (452 mg, 58% yield) and **13** (546 mg, 33% yield) both as white foams.

Data for **12**: ^{1}H NMR (600 MHz, DMSO-d_6) δ 11.22 (s, 1H), 7.65 (d, *J* = 8.1 Hz, 1H), 5.58 (d, *J* = 8.0 Hz, 1H), 4.95 (d, *J* = 6.9 Hz, 1H), 4.56 (td, *J* = 10.5, 8.4 Hz, 1H), 4.15 (ddd, *J* = 9.8, 6.9, 5.4 Hz, 1H), 3.68 (d, *J* = 6.8 Hz, 2H), 3.33 (s, 3H), 2.13 – 2.06 (m, 1H), 1.99 (dt, *J* = 12.7, 8.6 Hz, 1H), 1.25 (ddd, *J* = 12.7, 10.8, 7.8 Hz, 1H), 0.97 (s, 18H) ppm. ^{13}C NMR (151 MHz, DMSO-d_6) δ 163.7, 151.8, 143.3, 101.5, 81.4, 72.9, 65.0, 60.8, 57.5, 42.7, 28.1, 28.0, 27.5, 20.9, 20.8 ppm. HRMS calc. for $C_{19}H_{33}N_2O_5Si$ $[M+H]^+$: 397.2159; found: 397.2151.

Data for **13**: ^{1}H NMR (600 MHz, DMSO-d_6) δ 11.28 (s, 1H), 7.69 (d, *J* = 8.0 Hz, 1H), 5.58 (dd, *J* = 7.9, 2.3 Hz, 1H), 4.43 (td, *J* = 9.4, 1.9 Hz, 1H), 4.30 (dd, *J* = 11.2, 5.7 Hz, 1H), 4.16 (dd, *J* = 10.2, 4.6 Hz, 1H), 3.88 – 3.82 (m, 1H), 3.76 (dd, *J* = 5.8, 1.9 Hz, 1H), 3.40 (s, 3H), 2.28 – 2.15 (m, 1H), 1.90 (ddd, *J* = 12.1, 9.0, 5.9 Hz, 1H), 1.30 (td, *J* = 12.3, 9.7 Hz, 1H), 1.07 – 0.98 (m, 19H), 0.93 (s, 17H) ppm. ^{13}C NMR (151 MHz, DMSO-d_6) δ 163.8, 151.0, 145.0, 102.1, 83.7, 79.5, 68.6, 62.9, 58.4, 28.1, 27.7, 27.4, 22.7, 20.3, 20.2 ppm. HRMS calc. for $C_{27}H_{52}N_2O_7Si_2Na$ $[M+Na]^+$: 595.3211; found: 595.3219.

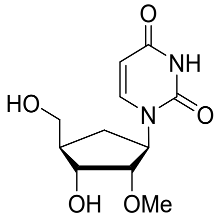

10

<u>(1S)-1-[(1R,2S,3R)-3-hydroxy-4-(hydroxymethyl)-2-methoxy-cyclopentyl]pyrimidine-2,4-dione</u> **<u>10</u>**: To a clear solution of **12** (300 mg, 756.52 μmol) in anhydrous THF (5 mL) was added TBAF (1 M in THF) (0.8 mL) at 0 °C, and the reaction mixture was stirred at room temperature for 3 h. TLC showed complete consumption of starting material. Solvent was removed under vacuum, and the residue was purified by silica gel column chromatography (5-20% MeOH in DCM) to afford **10** (171 mg, 88% yield) as a yellowish white foam. ^{1}H NMR (600 MHz, DMSO-d_6) δ 11.24 (s, 1H), 7.70 (d, *J* = 8.0 Hz, 1H), 5.60 (d, *J* = 8.0 Hz, 1H), 4.80 – 4.74 (m, 1H), 4.72 (t, *J* = 5.2 Hz, 1H), 4.62 (d, *J* = 4.7 Hz, 1H), 3.94 (td, *J* = 4.8, 2.6 Hz, 1H), 3.72 (dd, *J* = 9.5, 4.9 Hz, 1H), 3.43 – 3.36 (m, 2H), 3.25 (s, 3H), 2.04 (dt, *J* = 12.8, 8.9 Hz, 1H), 1.95 (td, *J* = 6.7, 3.4 Hz, 1H), 1.26 (ddd, *J* = 12.8, 10.4, 7.2 Hz, 1H) ppm. ^{13}C NMR (151 MHz, DMSO-d_6) δ 163.2, 151.2, 142.4, 101.5, 82.6, 68.9, 62.8, 58.0, 56.9, 54.9, 45.0, 27.6 ppm. HRMS calc. for $C_{11}H_{17}N_2O_5$ $[M+H]^+$: 257.1137; found: 257.1147.

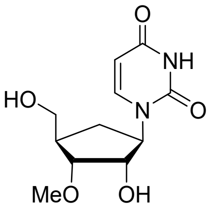

11

<u>1-[(1R,4R)-2-hydroxy-4-(hydroxymethyl)-3-methoxy-cyclopentyl]pyrimidine-2,4-dione</u> **<u>15</u>**: To a clear solution of **13** (1.5 g, 1.79 mmol) in anhydrous THF (10 mL) was added TBAF (1 M in THF, 5.37 mL, 5.37 mmol) at 0 °C, and the mixture was stirred at room temperature for 3 h. TLC showed complete consumption of starting material. The reaction mixture was concentrated, and the crude

product was purified by silica gel column chromatography (0-15% MeOH in DCM) to afford **11** (384 mg, 84% yield) as a white foam. ^{1}H NMR (600 MHz, DMSO-d_6) δ 11.22 (s, 1H), 7.67 (d, *J* = 8.0 Hz, 1H), 5.59 (dd, *J* = 8.0, 1.9 Hz, 1H), 4.90 (d, *J* = 7.0 Hz, 1H), 4.75 (t, *J* = 5.2 Hz, 1H), 4.60 (td, *J* = 10.3, 7.8 Hz, 1H), 4.10 (ddd, *J* = 9.9, 6.9, 5.3 Hz, 1H), 3.41 – 3.36 (m, 3H), 3.32 (s, 3H), 3.19 – 3.13 (m, 1H), 2.04 – 1.94 (m, 2H), 1.56 (q, *J* = 8.1 Hz, 1H), 1.31 (q, *J* = 7.4 Hz, 1H), 1.26 – 1.20 (m, 1H), 0.94 (t, *J* = 7.4 Hz, 2H) ppm. ^{13}C NMR (151 MHz, DMSO-d_6) δ 163.2, 151.4, 142.6, 101.2, 81.3, 72.9, 62.8, 59.9, 56.9, 42.0, 27.3, 23.1, 19.2, 13.5 ppm. HRMS calc. for $C_{11}H_{17}N_2O_5$ [M+H]$^+$: 257.1137; found: 257.1134.

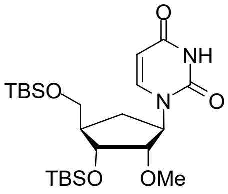

14

1-[3-[1,1-di(methyl)ethyl-di(methyl)silyl]oxy-4-[[1,1-di(methyl)ethyl-di(methyl)silyl]oxymethyl]-2-methoxy-1,2,3,4-tetratritio-cyclopentyl]pyrimidine-2,4-dione **14**: To a clear solution of compound **10** (220 mg, 858.52 μmol) in anhydrous DMF (10 mL) were added TBS chloride (386.33 mg, 2.58 mmol) and imidazole (233.79 mg, 3.43 mmol), and the reaction mixture was allowed to stir at room temperature for 3 h. The reaction was quenched with aq. saturated $NaHCO_3$ solution, and the mixture was diluted with diethyl ether. The organic layer was washed with water and brine and then dried over Na_2SO_4 and filtered. The solvent was removed, and the crude product was purified by silica gel column chromatography (20-50% EtOAc in hexanes) to afford **14** (379 mg, 91% yield) as a white foam. ^{1}H NMR (600 MHz, CD_3CN) δ 9.37 (s, 1H), 7.40 (d, *J* = 8.1 Hz, 1H), 5.61 (d, *J* = 8.0 Hz, 1H), 4.77 (q, *J* = 9.2 Hz, 1H), 4.18 (dd, *J* = 4.6, 3.0 Hz, 1H), 3.72 (dd, *J* = 8.7, 4.6 Hz, 1H), 3.66 (dd, *J* = 10.3, 4.8 Hz, 1H), 3.59 (dd, *J* = 10.3, 6.1 Hz, 1H), 3.29 (s, 3H), 2.25 – 2.14 (m, 1H), 2.10 (dddd, *J* = 9.5, 8.3, 4.1, 2.4 Hz, 1H), 1.38 (ddd, *J* = 13.4, 9.7, 6.8 Hz, 1H), 0.91 (d, *J* = 7.8 Hz, 18H), 0.11 – 0.08 (m, 12H) ppm. ^{13}C NMR (151 MHz, CD_3CN) δ 163.8, 151.8, 142.9, 102.1, 83.8, 71.8, 64.6, 59.7, 57.5, 46.1, 27.4, 26.0, 25.8, 18.6, 18.3, -4.8, -4.8, -5.6, -5.6 ppm. HRMS calc. for $C_{23}H_{45}N_2O_5Si_2$ [M+H]$^+$: 485.2867; found: 485.2853.

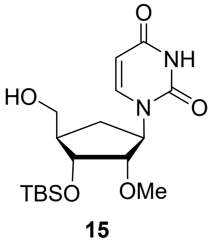

15

1-[3-[1,1-di(methyl)ethyl-di(methyl)silyl]oxy-2-methoxy-4-(oxidanylmethyl)-1,2,3,4-tetratritio-cyclopentyl]pyrimidine-2,4-dione **15**: To a clear solution of **14** (360 mg, 742.61 μmol) in anhydrous EtOH (7 mL) was added pyridinium *p*-toluenesulfonate (46.65 mg, 185.65 μmol), and the reaction was stirred at room temperature for 48 h. TLC showed that protecting groups were removed. The mixture was concentrated, and the crude product was purified by silica gel column chromatography (0-10% MeOH in DCM) to afford **15** (191 mg, 69% yield) as a white foam. ^{1}H NMR (600 MHz, CD_2Cl_2) δ 7.17 (d, *J* = 6.1 Hz, 1H), 5.47 (d, *J* = 6.6 Hz, 1H), 4.39 (q, *J* = 9.3 Hz,

1H), 3.96 (d, J = 4.0 Hz, 1H), 3.68 (dt, J = 7.4, 3.6 Hz, 1H), 3.47 (dt, J = 7.1, 3.4 Hz, 1H), 3.44 – 3.37 (m, 1H), 3.15 – 3.04 (m, 3H), 2.13 – 2.02 (m, 1H), 1.98 – 1.90 (m, 1H), 1.49 (dt, J = 14.7, 7.8 Hz, 1H), 0.68 (d, J = 2.2 Hz, 9H), -0.13 (d, J = 3.1 Hz, 6H) ppm. ^{13}C NMR (151 MHz, CD_2Cl_2) δ 164.5, 151.8, 144.4, 102.4, 83.9, 72.6, 63.8, 62.9, 58.0, 46.2, 26.9, 25.9, 18.4, -4.6, -4.6 ppm. HRMS calc. for $C_{17}H_{31}N_2O_5Si$ $[M+H]^+$: 371.2002; found: 371.1993.

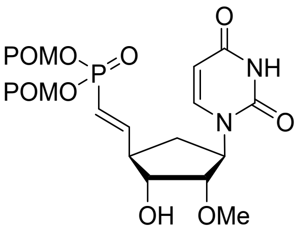

18

[[(*E*)-2-[4-[2,4-bis(oxidanylidene)pyrimidin-1-yl]-3-methoxy-2-oxidanyl-1,2,3,4-tetratritio-cyclopentyl]vinyl]-[2,2-di(methyl)propanoyloxymethoxy]phosphoryl]oxymethyl-2,2-di(methyl)propanoate **18**: To a clear solution of **15** (191 mg, 515.50 μmol) in anhydrous acetonitrile (7 mL) was added IBX (259.81 mg, 927.90 μmol), and the reaction mixture was refluxed at 90 °C for 1 h. TLC showed complete consumption of the starting material. The reaction mixture was filtered through a celite pad, and the filtrate was concentrated and dried to yield crude aldehyde **16**. To a suspension of sodium hydride (60% dispersion in mineral oil; 23.70 mg, 1.03 mmol) in anhydrous THF (10 mL) at -78 °C was added tetra(pivaloyloxymethyl)-bis-phosphonate (651.59 mg, 1.03 mmol) dissolved in 3 mL THF. Aldehyde **16** was added at 0 °C, and the reaction mixture was stirred at room temperature for 3 h. The reaction was quenched with saturated aq. NH_4Cl solution (5 mL) and concentrated. The residue was dissolved in EtOAc (100 mL), washed with water and brine, and dried over Na_2SO_4. The organic layer was filtered and concentrated to yield the crude product **17**. The silyl was removed using (1:1) v/v of H_2O-HCOOH. The mixture was concentrated, and the crude product was purified by silica gel column chromatography (60-100% EtOAc in hexanes) to afford a mixture of isomers (as evident from ^{31}P NMR) (26 mg, 9% yield) and pure **18** (173 mg, 60% overall yield in three steps) as a white foam. ^{1}H NMR (600 MHz, CD_3CN) δ 9.44 – 9.37 (m, 1H), 7.37 (d, J = 8.1 Hz, 1H), 6.84 – 6.75 (m, 1H), 5.87 – 5.78 (m, 1H), 5.61 – 5.58 (m, 5H), 4.57 (td, J = 9.4, 5.6 Hz, 1H), 4.17 (dd, J = 6.7, 5.4 Hz, 1H), 3.77 (t, J = 5.5 Hz, 1H), 3.31 (s, 3H), 2.80 – 2.67 (m, 1H), 2.30 – 2.16 (m, 2H), 1.65 (dt, J = 13.2, 9.6 Hz, 1H), 1.19 (d, J = 5.4 Hz, 24H), 0.89 (s, 10H), 0.08 (d, J = 1.4 Hz, 6H) ppm. ^{13}C NMR (151 MHz, CD_3CN) δ 177.6, 177.6, 164.3, 155.4, 155.4, 151.8, 144.4, 118.3, 102.7, 83.8, 82.6, 82.6, 82.6, 74.8, 62.3, 58.3, 48.9, 48.8, 39.4, 30.9, 27.1 ppm. ^{31}P NMR (243 MHz, CD_3CN) δ 17.86 ppm. ^{31}P NMR for (*E*-*Z*) mixture (243 MHz, CD_3CN) δ 17.52, 15.84 ppm. HRMS calc. for $C_{24}H_{38}N_2O_{11}P$ $[M+H]^+$: 561.2213; found: 561.2203.

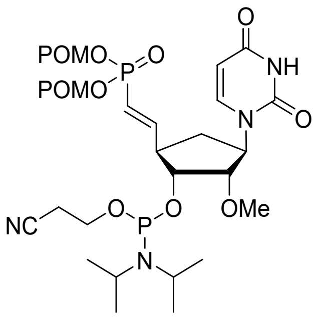

19

[[(*E*)-2-[2-[2-(azanylidynemethyl)ethoxy-[bis(1-methylethyl)amino]phosphanyl]oxy-4-[2,4-bis(oxidanylidene)pyrimidin-1-yl]-3-methoxy-1,2,3,4-tetratritio-cyclopentyl]vinyl]-[2,2-di(methyl)propanoyloxymethoxy]phosphoryl]oxymethyl-2,2-di(methyl)propanoate **19**: To a clear solution of **18** (150 mg, 267.60 μmol) in anhydrous DCM (6 mL) were added ETT (34.79 mg, 267.60 μmol) and 2-cyanoethyl-*N,N,N',N'*-tetraisopropylphosphorodiamidite (80.55 mg, 267.60 μmol), and the mixture was stirred for 20 min. The mixture was concentrated, and the crude product was purified by silica gel column chromatography (60-100% EtOAc in hexanes) to afford **19** (173 mg, 85% yield) as a white foam. ^{1}H NMR (600 MHz, CD_3CN) δ 9.55 (s, 1H), 7.38 (dd, *J* = 8.0, 6.1 Hz, 1H), 6.92 – 6.80 (m, 1H), 5.92 – 5.79 (m, 1H), 5.62 – 5.58 (m, 4H), 4.61 (dtd, *J* = 11.4, 9.4, 6.4 Hz, 1H), 4.34 – 4.22 (m, 1H), 3.95 – 3.87 (m, 1H), 3.87 – 3.76 (m, 2H), 3.69 – 3.58 (m, 2H), 3.34 (d, *J* = 17.5 Hz, 3H), 3.00 – 2.87 (m, 1H), 2.71 – 2.65 (m, 2H), 2.33 – 2.25 (m, 1H), 1.69 (dtd, *J* = 13.4, 9.4, 5.6 Hz, 1H), 1.24 – 1.15 (m, 30H) ppm. ^{13}C NMR (151 MHz, CD_3CN) δ 177.5, 164.3, 154.8, 154.8, 151.9, 144.4, 118.3, 102.6, 102.6, 82.6, 76.4, 76.3, 75.8, 75.7, 62.5, 62.3, 59.8, 59.7, 59.3, 59.2, 58.3, 58.3, 58.1, 58.0, 43.9, 39.4, 30.5, 30.4, 27.1 ppm. ^{31}P NMR (243 MHz, CD_3CN) δ 149.13, 148.52, 17.44 ppm. HRMS calc. for $C_{33}H_{54}N_4O_{12}P_2Na$ $[M+Na]^+$: 783.3111; found: 783.3093.

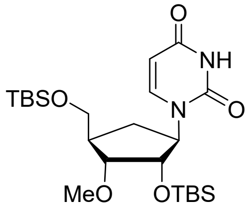

20

1-[2-[1,1-di(methyl)ethyl-di(methyl)silyl]oxy-4-[[1,1-di(methyl)ethyl-di(methyl)silyl]oxymethyl]-3-methoxy-1,2,3,4-tetratritio-cyclopentyl]pyrimidine-2,4-dione **20**: To a clear solution of **11** (233 mg, 909.25 μmol) in anhydrous DMF (10 mL) were added imidazole (247.61 mg, 3.64 mmol) and TBDMSCl (409.16 mg, 2.71 mmol), and the reaction mixture was allowed to stir at room temperature for 3 h. TLC showed complete consumption of starting material. The reaction mixture was diluted with diethyl ether and washed with water and brine. The organic layer was dried over Na_2SO_4, filtered, and concentrated. The crude product was purified by silica gel column chromatography (20-50 % EtOAc in hexanes) to afford **20** (403 mg, 91 % yield) as a white foam. ^{1}H NMR (600 MHz, CD_3CN) δ 9.31 (s, 1H), 7.38 (d, *J* = 8.1 Hz, 1H), 5.62 (dd, *J* = 8.0, 2.0 Hz, 1H), 4.74 (td, *J* = 10.1, 9.0 Hz, 1H), 4.20 (dd, *J* = 9.7, 5.0 Hz, 1H), 3.68 (dd, *J* = 10.2, 4.5 Hz, 1H), 3.57 (dd, *J* = 10.2, 6.4 Hz, 1H), 3.43 (dd, *J* = 5.0, 1.9 Hz, 1H), 3.35 (s, 3H), 2.22 – 2.14 (m, 1H), 2.09 (dt, *J* = 13.3, 9.3 Hz, 1H), 1.47 (ddd, *J* = 13.3, 10.4, 6.7 Hz, 1H), 0.93 (s, 9H), 0.83 (s, 9H), 0.09 (s, 6H), 0.04 (s, 3H), -0.03 (s, 3H). ^{1}H NMR (600 MHz, CD_3CN) δ 9.31 (s, 1H), 7.38 (d, *J* =

8.1 Hz, 1H), 5.62 (dd, J = 8.0, 2.0 Hz, 1H), 4.74 (td, J = 10.1, 9.0 Hz, 1H), 4.20 (dd, J = 9.7, 5.0 Hz, 1H), 3.68 (dd, J = 10.2, 4.5 Hz, 1H), 3.57 (dd, J = 10.2, 6.4 Hz, 1H), 3.43 (dd, J = 5.0, 1.9 Hz, 1H), 3.35 (s, 3H), 2.22 – 2.14 (m, 1H), 2.09 (dt, J = 13.3, 9.3 Hz, 1H), 1.47 (ddd, J = 13.3, 10.4, 6.7 Hz, 1H), 0.93 (s, 9H), 0.83 (s, 9H), 0.09 (s, 6H), 0.04 (s, 3H), -0.03 (s, 3H) ppm. ^{13}C NMR (151 MHz, CD_3CN) δ 163.7, 151.9, 143.0, 102.0, 82.5, 76.0, 65.1, 61.4, 57.4, 42.5, 25.9, 25.6, 18.5, 18.1, -4.8, -5.2, -5.6, -5.6 ppm. HRMS calc. for $C_{23}H_{45}N_2O_5Si_2$ $[M+H]^+$: 485.2867; found: 485.2889.

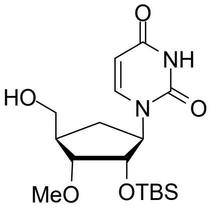

21

1-[2-[1,1-di(methyl)ethyl-di(methyl)silyl]oxy-3-methoxy-4-(oxidanylmethyl)-1,2,3,4-tetratritio-cyclopentyl]pyrimidine-2,4-dione **21**: To a clear solution of **20** (381 mg, 785.93 µmol) in anhydrous EtOH (10 mL) was added pyridinium *p*-toluenesulfonate (49.38 mg, 196.48 µmol), and the reaction mixture was allowed to stir at room temperature for 48 h. The solvent was evaporated to dryness, and the residue was purified by silica gel column chromatography (0-10% MeO in DCM) to afford **21** (171 mg, 59 % yield) as a white foam. ^{1}H NMR (600 MHz, DMSO-d_6) δ 11.23 (s, 1H), 7.72 (d, J = 8.0 Hz, 1H), 5.61 (dd, J = 7.9, 2.3 Hz, 1H), 4.82 (t, J = 5.2 Hz, 1H), 4.72 (td, J = 10.3, 8.7 Hz, 1H), 4.26 (dd, J = 10.0, 5.0 Hz, 1H), 3.43 – 3.36 (m, 3H), 3.31 (s, 3H), 2.08 – 2.02 (m, 1H), 1.96 (dt, J = 12.9, 9.1 Hz, 1H), 1.36 (ddd, J = 12.9, 10.6, 6.6 Hz, 1H), 0.79 (s, 9H), 0.01 (s, 3H), -0.07 (s, 3H) ppm.^{13}C NMR (151 MHz, DMSO-d_6) δ 163.1, 151.3, 142.9, 101.3, 81.4, 74.8, 62.9, 59.9, 56.9, 42.1, 26.0, 25.5, 17.6, -4.7, -5.1 ppm. HRMS calc. for $C_{17}H_{31}N_2O_5Si$ $[M+H]^+$: 371.2002; found: 371.2007.

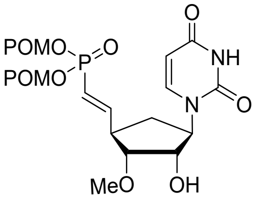

24

[[(*E*)-2-[4-[2,4-bis(oxidanylidene)pyrimidin-1-yl]-2-methoxy-3-oxidanyl[1,1-1,2,3,4-tetratritio-cyclopentyl]vinyl]-[2,2-di(methyl)propanoyloxymethoxy]phosphoryl]oxymethyl-2,2-di(methyl) propanoate **24**: To a clear solution of **21** (201 mg, 542.50 µmol) in anhydrous acetonitrile (8 mL) was added IBX (272.80 mg, 974.30 µmol), and the reaction mixture was refluxed at 90 °C for 1 h. TLC showed complete consumption of the starting material. The reaction mixture was filtered through a celite pad, and the filtrate was concentrated and dried to yield crude aldehyde **22**. To a suspension of sodium hydride (60% dispersion in mineral oil; 24.89 mg, 1.08 mmol) in anhydrous THF (10 mL) at -78 °C was added tetra(pivaloyloxymethyl)-bis-phosphonate (684.17 mg, 1.08 mmol) dissolved in 3 mL THF. Aldehyde **22** was added at 0 °C, and the reaction mixture was stirred at room temperature for 3 h. The reaction was quenched with saturated aq. NH_4Cl solution (5 mL) and concentrated. The residue was dissolved in EtOAc (100 mL), washed with water and brine, and dried over Na_2SO_4. The organic layer was filtered and concentrated to yield

the crude product **23**. The silyl was removed using H_2O/HCOOH (1:1, v/v). The mixture was concentrated, and the crude product was purified by silica gel column chromatography (60-100% EtOAc in hexanes) to afford a mixture of isomers (as evident from ^{31}P NMR) (24 mg, 8% yield) and pure **24** (167 mg, 55% overall yield in three steps) as a white foam. ^{1}H NMR (600 MHz, CD_3CN) δ 9.41 (s, 1H), 7.37 (d, *J* = 8.1 Hz, 1H), 6.89 – 6.79 (m, 1H), 5.90 – 5.81 (m, 1H), 5.61 (s, 4H), 4.45 (ddd, *J* = 10.4, 8.4, 6.2 Hz, 1H), 4.25 (t, *J* = 6.0 Hz, 1H), 3.64 (t, *J* = 6.1 Hz, 1H), 3.38 (s, 3H), 3.28 (h, *J* = 5.6 Hz, 1H), 2.86 – 2.79 (m, 1H), 2.19 (dt, *J* = 13.0, 8.1 Hz, 1H), 1.68 (dt, *J* = 13.1, 10.4 Hz, 1H), 1.19 (d, *J* = 3.5 Hz, 18H) ppm. ^{13}C NMR (151 MHz, CD_3CN) δ 177.6, 164.3, 155.4, 155.4, 152.1, 144.5, 118.3, 102.4, 84.4, 82.6, 82.6, 82.6, 73.7, 64.7, 58.4, 46.1, 45.9, 39.4, 30.7, 27.1 ppm. ^{31}P NMR (243 MHz, CD_3CN) δ 17.78 ppm. ^{31}P NMR for (*E*-*Z*) mixture (243 MHz, CD_3CN) δ 17.578, 15.89 ppm. HRMS calc. for $C_{24}H_{37}N_2O_{11}PNa$ $[M+Na]^+$: 583.2033; found: 583.2039.

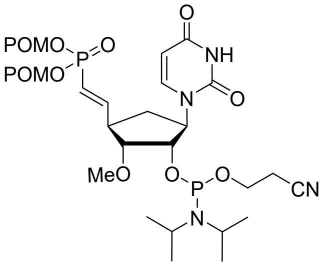

25

[[(*E*)-2-[3-[2-(azanylidynemethyl)ethoxy-[bis(1-methylethyl)amino]phosphanyl]oxy-4-[2,4-bis(oxidanylidene)pyrimidin-1-yl]-2-methoxy-1,2,3,4-tetratritio-cyclopentyl]vinyl]-[2,2-di(methyl)propanoyloxymethoxy]phosphoryl]oxymethyl 2,2-di(methyl)propanoate **25**: To a clear solution of **24** (143 mg, 255.12 µmol) in anhydrous DCM (6 mL) were added ETT (33.17 mg, 255.12 µmol) and 2-cyanoethyl-*N,N,N',N'*-tetraisopropylphosphorodiamidite (76.79 mg, 255.12 µmol), and the reaction mixture was allowed to stir for 20 min. The mixture was concentrated, and the crude product was purified by silica gel column chromatography (60-100% EtOAc in hexanes) to afford **25** (161 mg, 83% yield) as a white foam. ^{1}H NMR (600 MHz, CD_3CN) δ 9.48 (s, 1H), 7.38 (d, *J* = 8.0 Hz, 1H), 6.91 – 6.78 (m, 1H), 5.90 – 5.81 (m, 1H), 5.63 – 5.57 (m, 5H), 4.71 – 4.53 (m, 1H), 4.48 (dt, *J* = 8.0, 3.9 Hz, 1H), 4.15 – 4.01 (m, 1H), 3.85 – 3.56 (m, 6H), 3.53 – 3.45 (m, 1H), 3.42 (s, 1H), 3.36 (s, 2H), 2.87 (dt, *J* = 17.7, 7.7 Hz, 1H), 2.76 (t, *J* = 6.0 Hz, 1H), 2.69 – 2.64 (m, 1H), 2.60 (td, *J* = 6.1, 1.8 Hz, 1H), 2.21 (ddd, *J* = 15.2, 7.8, 4.8 Hz, 1H), 1.81 – 1.67 (m, 1H), 1.19 (s, 18H), 1.17 – 1.12 (m, 10H), 1.07 (d, *J* = 6.8 Hz, 3H) ppm. ^{13}C NMR (151 MHz, CD_3CN) δ 177.5, 164.4, 164.2, 159.5, 155.4, 155.4, 155.3, 155.2, 151.9, 151.9, 145.5, 144.5, 131.0, 131.0, 131.0, 129.0, 128.9, 128.8, 118.3, 114.0, 102.6, 102.3, 84.1, 83.9, 83.9, 82.6, 82.6, 82.6, 82.5, 75.8, 75.6, 75.5, 75.4, 65.4, 59.5, 59.4, 59.2, 59.1, 59.1, 59.0, 58.7, 58.7, 58.3, 58.2, 46.4, 46.2, 46.0, 45.9, 44.0, 44.0, 43.9, 43.9, 39.4, 30.1, 29.8, 27.1, 25.0, 24.9, 24.9, 24.9, 24.8, 24.8, 24.8, 23.2, 23.1, 23.1, 23.1, 21.0, 20.9, 20.9, 20.9, 20.6, 20.5 ppm. ^{31}P NMR (243 MHz, CD_3CN) δ 149.63, 149.60, 17.77 ppm. HRMS calc. for $C_{33}H_{55}N_4O_{12}P_2$ $[M+H]^+$: 761.3292; found: 761.3324.

Oligonucleotide synthesis, purification, and characterization

For preparation of siRNAs targeting *Ttr* and *ApoB* mRNA, oligonucleotides were synthesized on a ABI DNA/RNA synthesizer at scales between 1−10 μmol using commercially available 5′-O-(4,4′-dimethoxytrityl)-2′-fluoro- and 5′-O-(4,4′-dimethoxytrityl)-2′-O-methyl-3′-O-(2-cyanoethyl-*N,N*-diisopropyl)phosphoramidite monomers of uridine, 4-*N*-acetylcytidine, 6-*N*-benzoyladenosine, and 2-N-isobutyrylguanosine using standard solid-phase oligonucleotide synthesis protocols.[1] The GalNAc ligand was introduced at the 3′ end of the sense strand of the siRNA using a functionalized solid support as described.[2] For 5′ terminal modification, the (n − 1)-mer of the desired antisense strand was synthesized on a solid-support under standard solid-phase synthesis conditions, and the 5′-DMTr was removed. The VP-modified phosphoramidite (**7a, 7b, 19**, or **25**) was then introduced at the 5′ terminus of the solid-support-bound oligonucleotide using 0.25 M 5-(ethylthio)-1*H*-tetrazole in acetonitrile as phosphoramidite activator to obtain the desired 5′-phosphonate-modified, full-length antisense strand. If desired, the phosphite triester was converted into a phosphorothioate linkage with 0.1 M DDTT. No DMT removal step was required as VP-containing phosphoramidites lack the DMT group. After completion of synthesis, the 5′-phosphonate-modified oligonucleotides were deprotected in 30% ammonia solution containing 5% DEA (v/v) for 5 h at 60 °C. The crude oligonucleotides were purified by IEX-HPLC using a column packed with TSK-Gel Super Q-5PW support and a linear gradient of 15−45 % buffer B over 120 min with 5 mL/min flow rate (buffer A, 0.02 M Na_2HPO_4 in 15% acetonitrile, pH 8.5; buffer B, buffer A plus 1 M NaBr). All single strands were purified to >90% based on HPLC (260 nm).

Oligonucleotides were desalted by size exclusion chromatography on an AKTA desaltification system using a HiPrep 26/10 packed with Sephadex G25 resin (GE Healthcare) with elution with sterile nuclease-free water at 10 mL/min. The isolated yields were calculated based on the ratios of measured to theoretical 260 nm optical density units. Extinction coefficients were calculated using the following extinction coefficients for each residue: A, 13.86; T/U, 7.92; C, 6.57; and G, 10.53 $M^{-1}cm^{-1}$. The identities of modified oligonucleotides were verified by mass spectrometry (Table S1). Purities were evaluated by analytical reverse-phase HPLC. For reverse-phase HPLC, a C-18 column was used with a gradient of 2-29% buffer B (buffer A, 95 mM hexafluoroisopropanol, 16.3 mM TEA, 0.05 mM EDTA; buffer B: methanol) over 39 min.

Equimolar amounts of complementary sense and antisense strands were annealed by heating in a water bath at 95 °C for 5 min and cooling to room temperature to obtain the desired siRNAs. The siRNA samples were analysed for purity, endotoxin, and osmolality, and the observed values were within the allowed range for the concentration tested.

Table S1: Analytical data for sense and antisense strands

Strand ID	Target	Single strand sequences (5′-3′)[a]	Single Strand Purity (IEX/RP-HPLC) Result [%]	ESI-MS (free acid)	
				Calcd. [Da]	Obsd. [Da]
**ON1**	*Ttr*	*A*•a•*C*a*G*u*G*u*UCU*u*G*c*U*c*U*a*U*a*A*L96	93.1	8590.2	8590.0
**ON2**		u•*U*•a*U*a*G*a*G*c*A*aga*A*c*A*c*U*g*U*u•u•u	92.0	7595.9	7595.2
**ON3**		**I**•*U*•a*U*a*G*a*G*c*A*aga*A*c*A*c*U*g*U*u•u•u	87.3	7671.9	7671.2
**ON4**		**III**•*U*•a*U*a*G*a*G*c*A*aga*A*c*A*c*U*g*U*u•u•u	94.8	7639.9	7639.3
**ON5**		**IV**•*U*•a*U*a*G*a*G*c*A*aga*A*c*A*c*U*g*U*u•u•u	97.3	7639.9	7639.0
**ON6**		**V**•*U*•a*U*a*G*a*G*c*A*aga*A*c*A*c*U*g*U*u•u•u	94.7	7671.0	7669.3
**ON7**		**VI**•*U*•a*U*a*G*a*G*c*A*aga*A*c*A*c*U*g*U*u•u•u	97.7	7671.0	7669.3
**ON8**		**III***U*•a*U*a*G*a*G*c*A*aga*A*c*A*c*U*g*U*u•u•u	91.7	7623.9	7623.1
**ON9**		**IV***U*•a*U*a*G*a*G*c*A*aga*A*c*A*c*U*g*U*u•u•u	97.1	7623.9	7623.1
**ON10**	*ApoB*	*C*•c•*U*g*G*a*C*a*UUC*a*G*a*A*c*A*a*G*a*A*L96	90.1	8697.4	8696.1
**ON11**		u•*U*•c*U*u*G*u*U*c*U*gaa*U*g*U*c*C*a*G*g•g•u	94.5	7557.8	7557.5
**ON12**		**I**•*U*•c*U*u*G*u*U*c*U*gaa*U*g*U*c*C*a*G*g•g•u	89.8	7633.8	7633.5
**ON13**		**III**•*U*•c*U*u*G*u*U*c*U*gaa*U*g*U*c*C*a*G*g•g•u	88.9	7601.8	7601.5
**ON14**		**V**•*U*•c*U*u*G*u*U*c*U*gaa*U*g*U*c*C*a*G*g•g•u	91.6	7632.9	7631.2
**ON15**		**VI**•*U*•c*U*u*G*u*U*c*U*gaa*U*g*U*c*C*a*G*g•g•u	97.4	7632.9	7631.2

[a] Upper case italics indicate 2'-F RNA; lower case indicates 2'-OMe modification; **I** indicates 5′-(*E*)-VP; **III** indicates 5′-(*E*)-VP-car-DNA; **IV** indicates 5′-(*Z*)-VP-car-DNA; **V** indicates 5′-(*E*)-VP-2′-OMe-car-RNA; **VI** indicates 5′-(*E*)-VP-3′-OMe-car-RNA; L indicates triantennary GalNAc; and • indicates a phosphorothioate linkage.

Analyses of siRNA activity in cultured primary mouse hepatocytes

Primary mouse hepatocytes were plated in 96-well format (20,000 cells/well) in hepatocyte plating medium (Primacyt). Each siRNA was tested in quadruplicate at 1 nM and 10 nM final concentrations. Cells were incubated 48 h with siRNA at 37 °C. Cells were lysed, and mRNA was quantified using the Quantigene Singleplex assay system (Thermo) according to the manufacturer's protocol. Probe sets for *Ttr*, *ApoB*, and *Gapdh* were designed by Thermo. Luminescent signal was read on a Victor light plate luminometer (Perkin Elmer). For each well, target mRNA level was normalized to the *Gapdh* mRNA level. The activity of a given siRNA was expressed as percent of target mRNA concentration[2] in treated cells relative to the target mRNA concentration averaged across control wells treated with a nonspecific siRNA.

Analyses of siRNA activity in mice

All studies were conducted following the animal welfare regulations of the state of Bavaria (Germany) and the European Union (guideline 2010/63/EU). Protocols were approved by the government of lower Franconia (Approval Nr. 55.2.2-2532-2-1548-20). Female C57/Bl6 mice were given a single subcutaneous injection of 0.5 mg/kg siRNA, prepared in an injection volume of 5 µL/g body weight in PBS. At the indicated time pre- or post-dosing, blood was obtained by puncturing the facial vein. TTR protein was quantified by ELISA from serum isolated from whole blood. The ELISA was performed according to the manufacturer's protocol (ALPCO, 41-PALMS-E01) after a 4000-fold dilution of the serum samples. TTR protein concentration was calculated from a standard curve.

^{1}H, ^{13}C, and ^{31}P NMR data for new compounds

^{1}H NMR (600 MHz, DMSO-d_6) of compound **2**

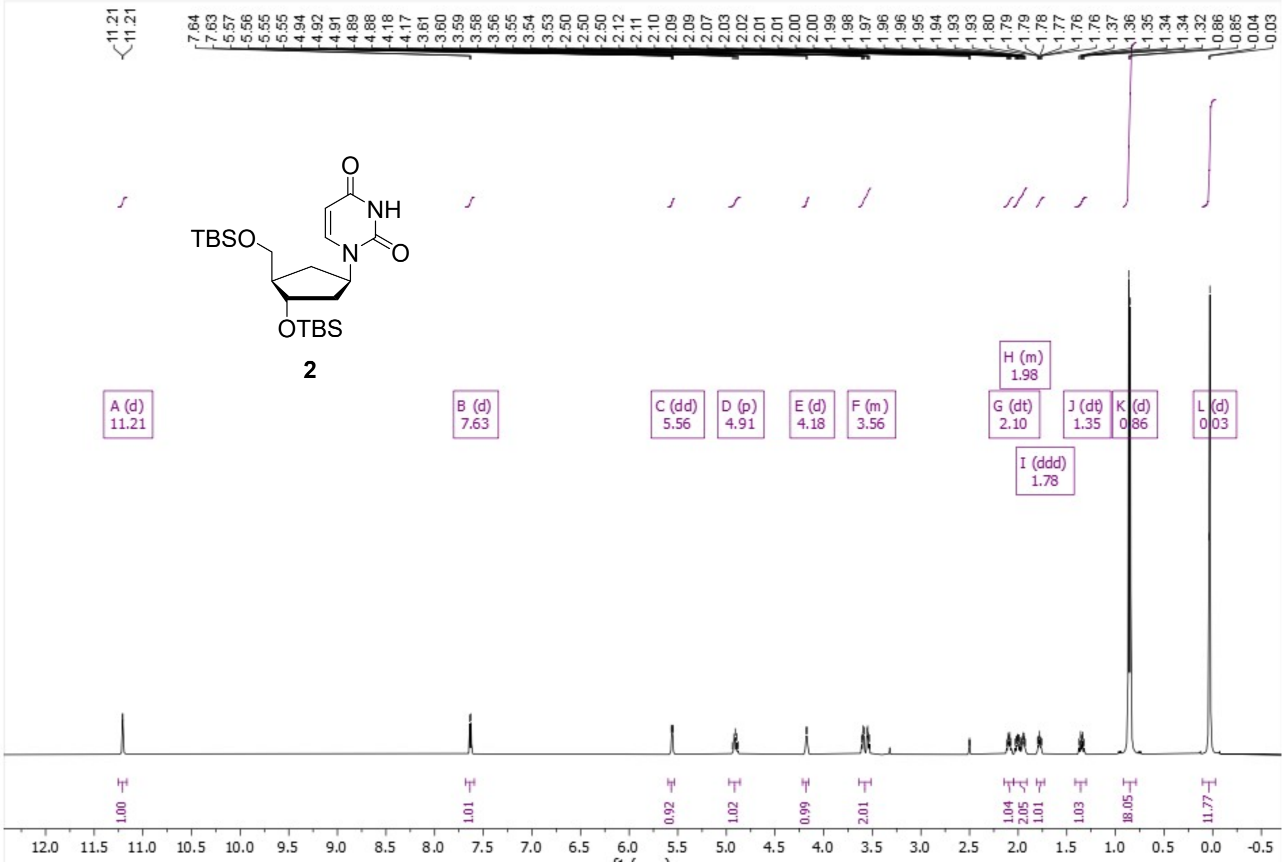

^{13}C NMR (151 MHz, DMSO-d_6) of compound **2**

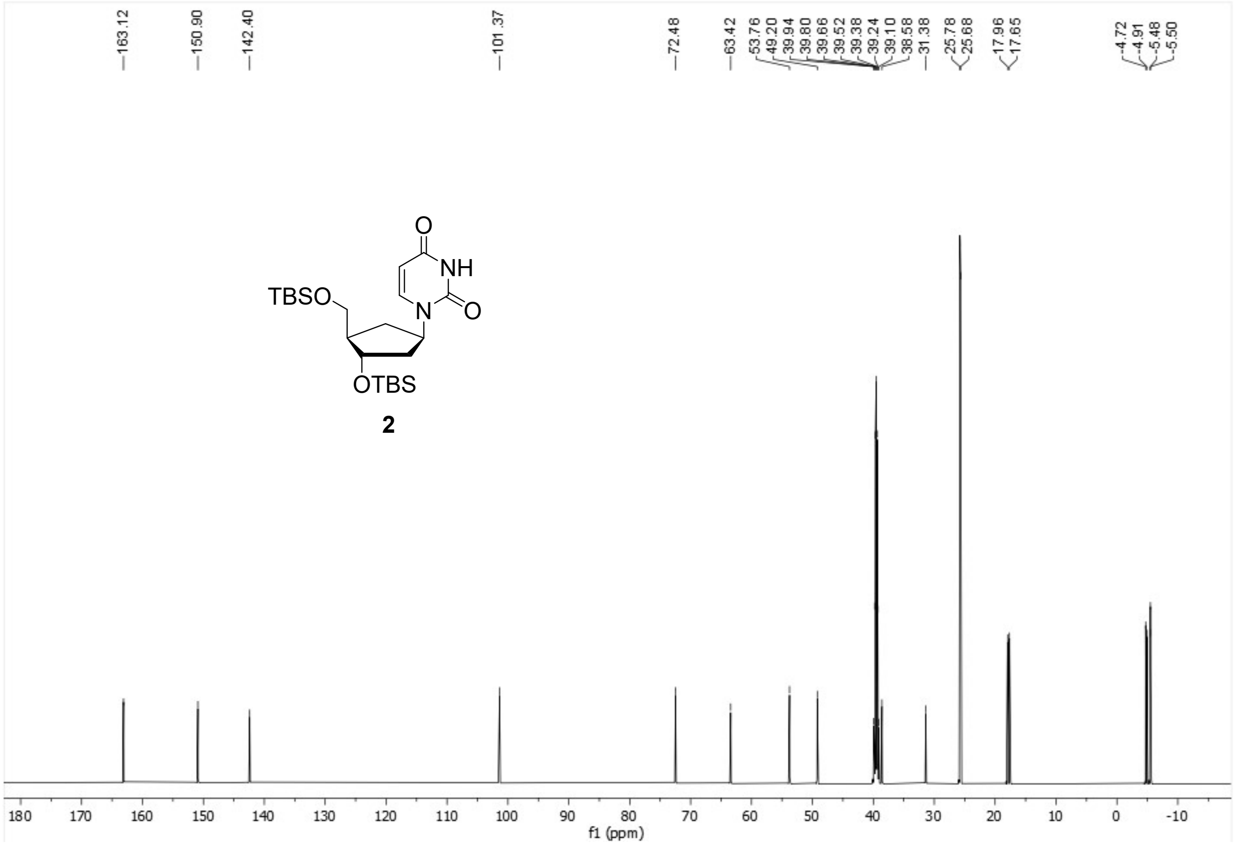

^{1}H NMR (600 MHz, DMSO-d_6) of compound **3**

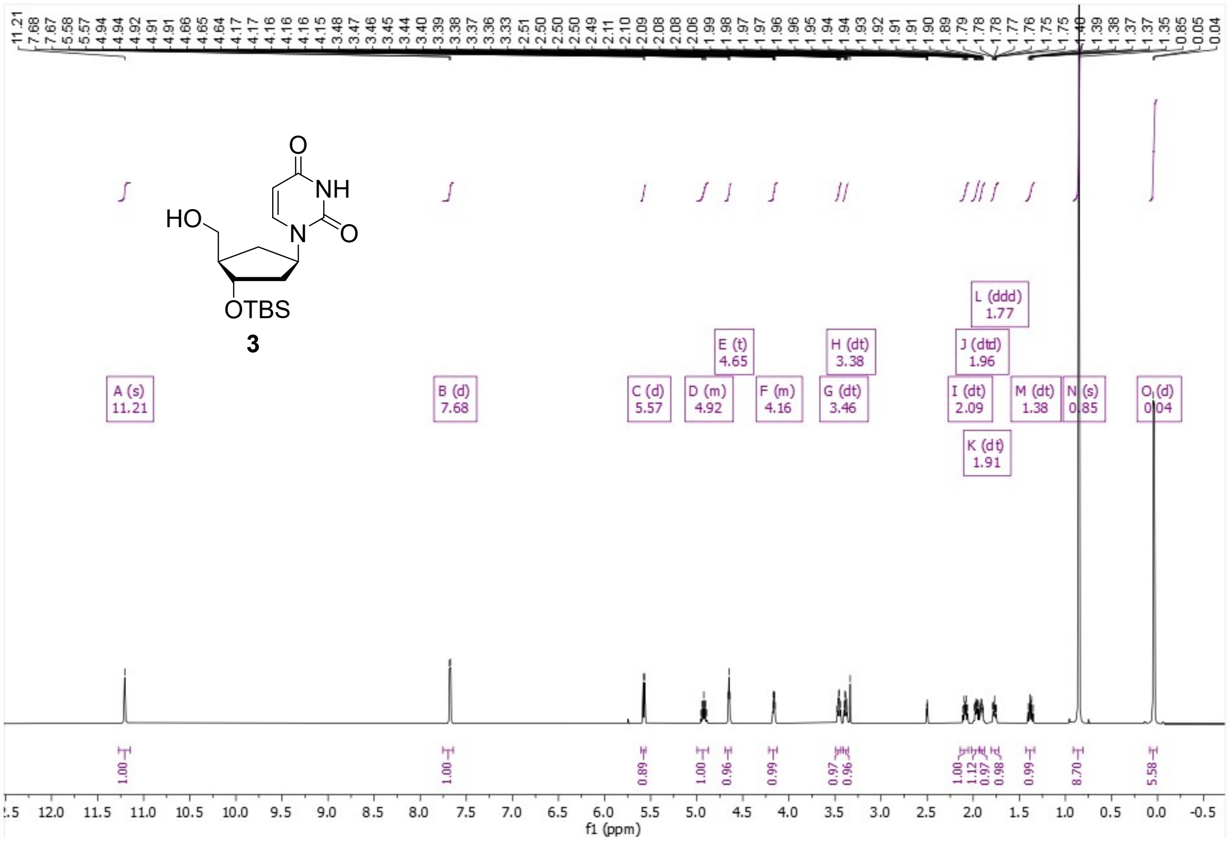

^{13}C NMR (151 MHz, DMSO-d_6) of compound **3**

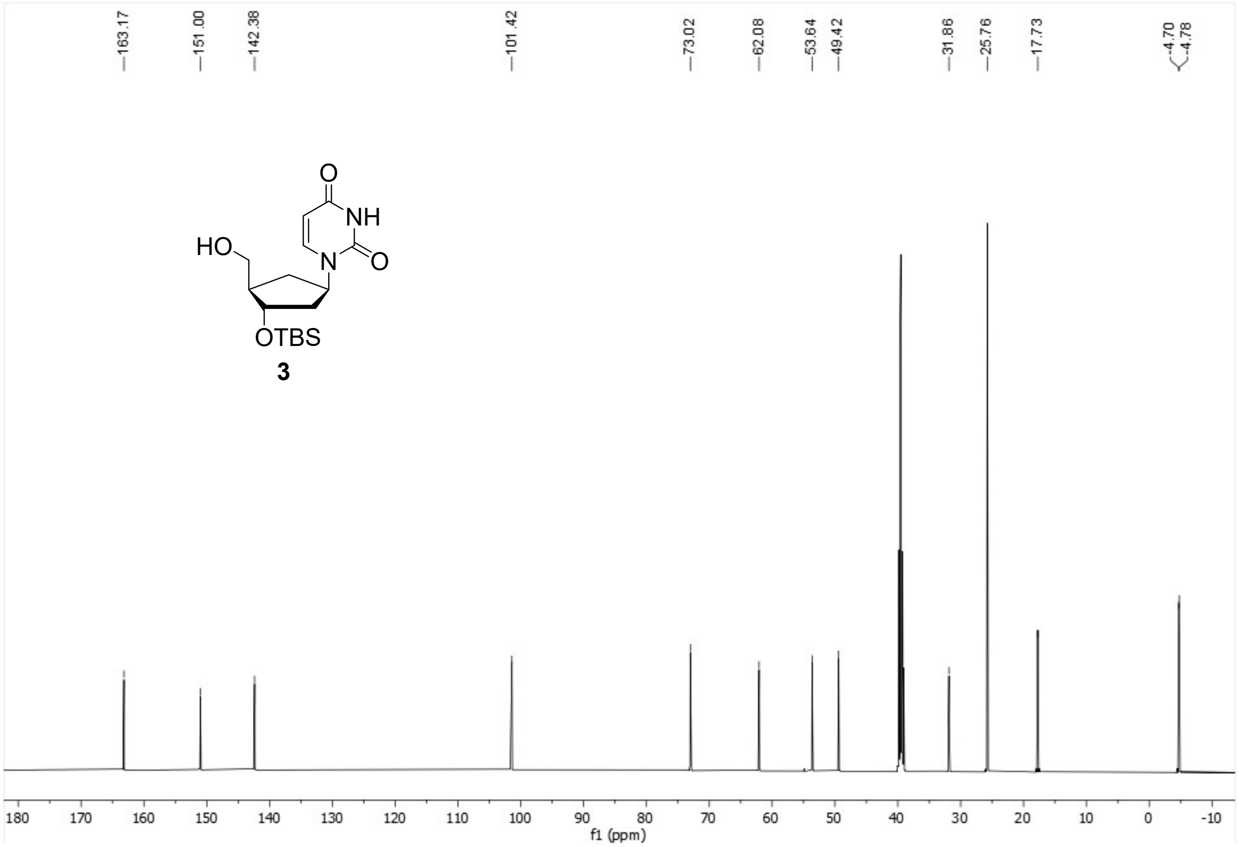

^{1}H NMR (600 MHz, DMSO-d_6) of compound **5a** and **5b**

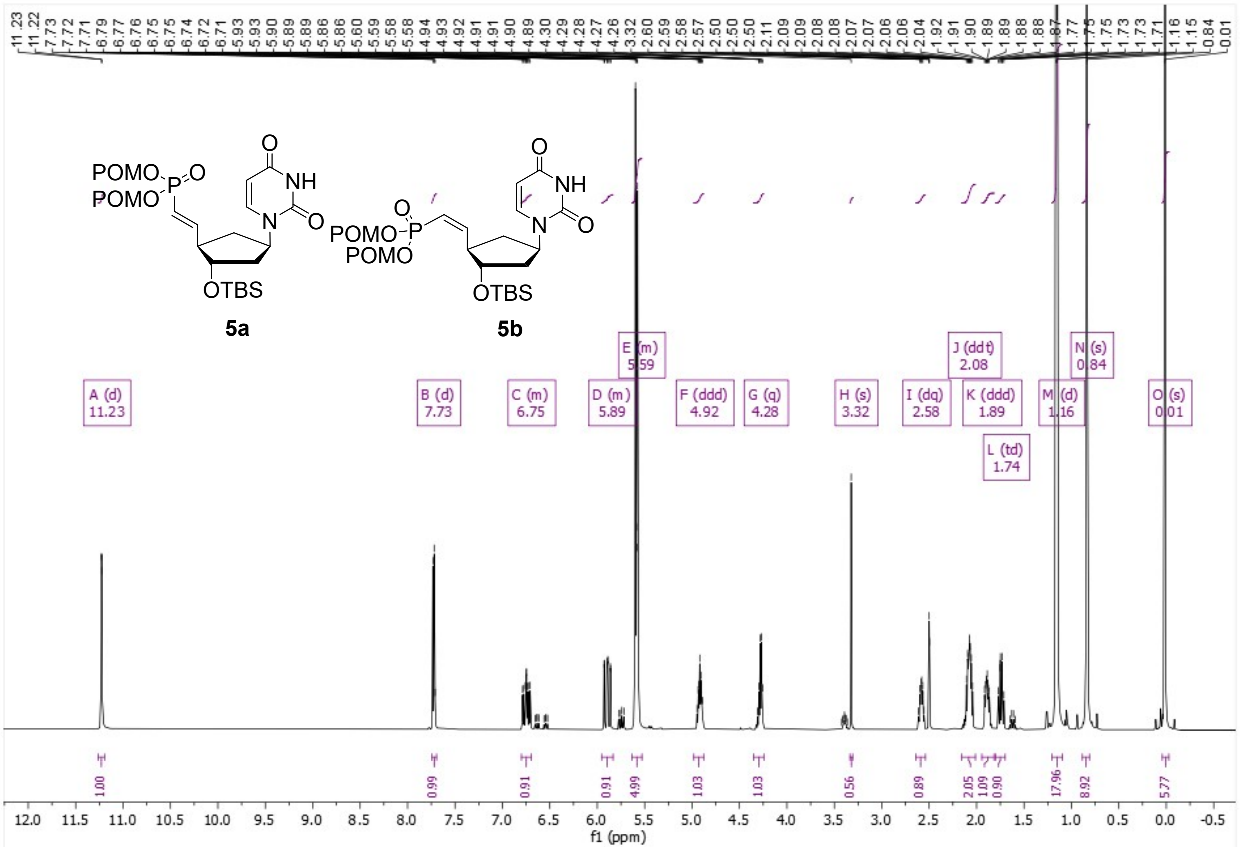

^{13}C NMR (151 MHz, DMSO-d_6) of compound **5a** and **5b**

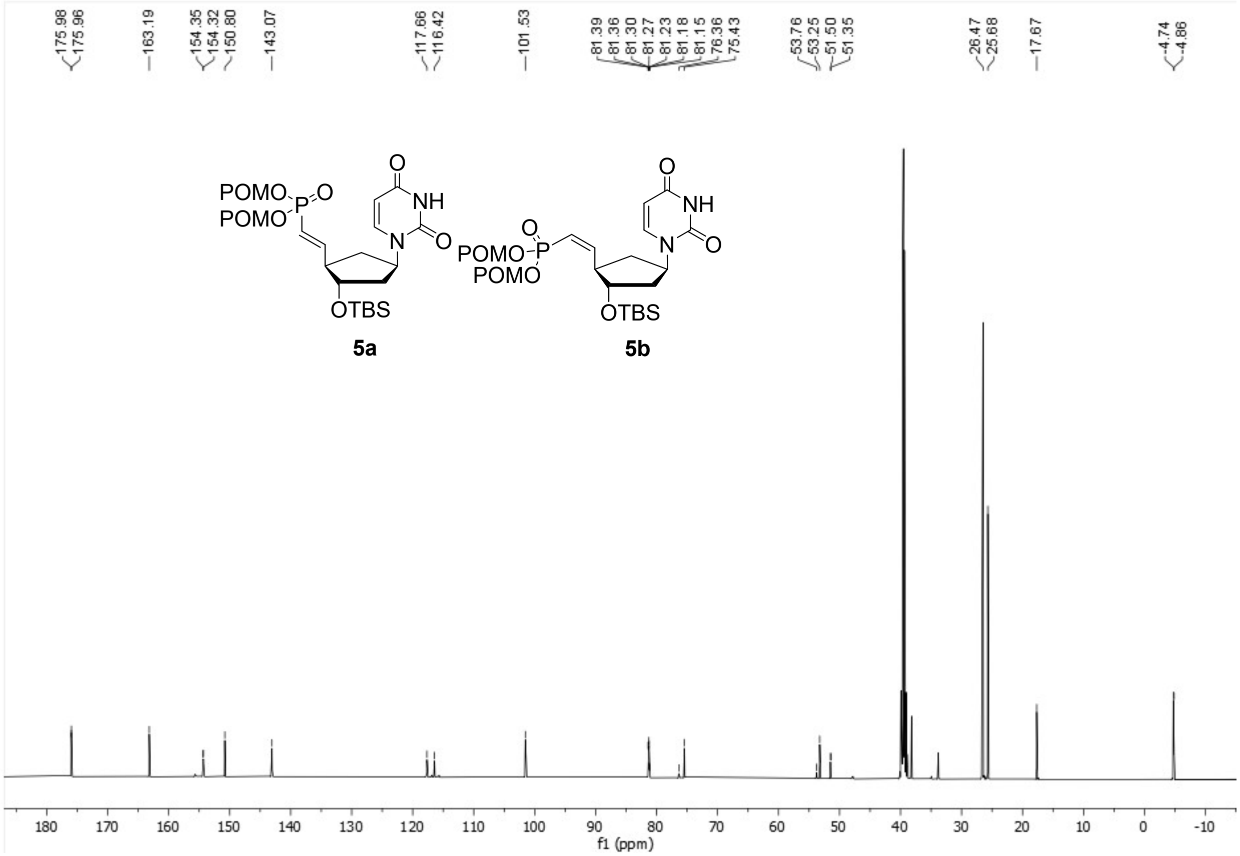

^{31}P NMR (243 MHz, DMSO-d_6) of compound **5a** and **5b**

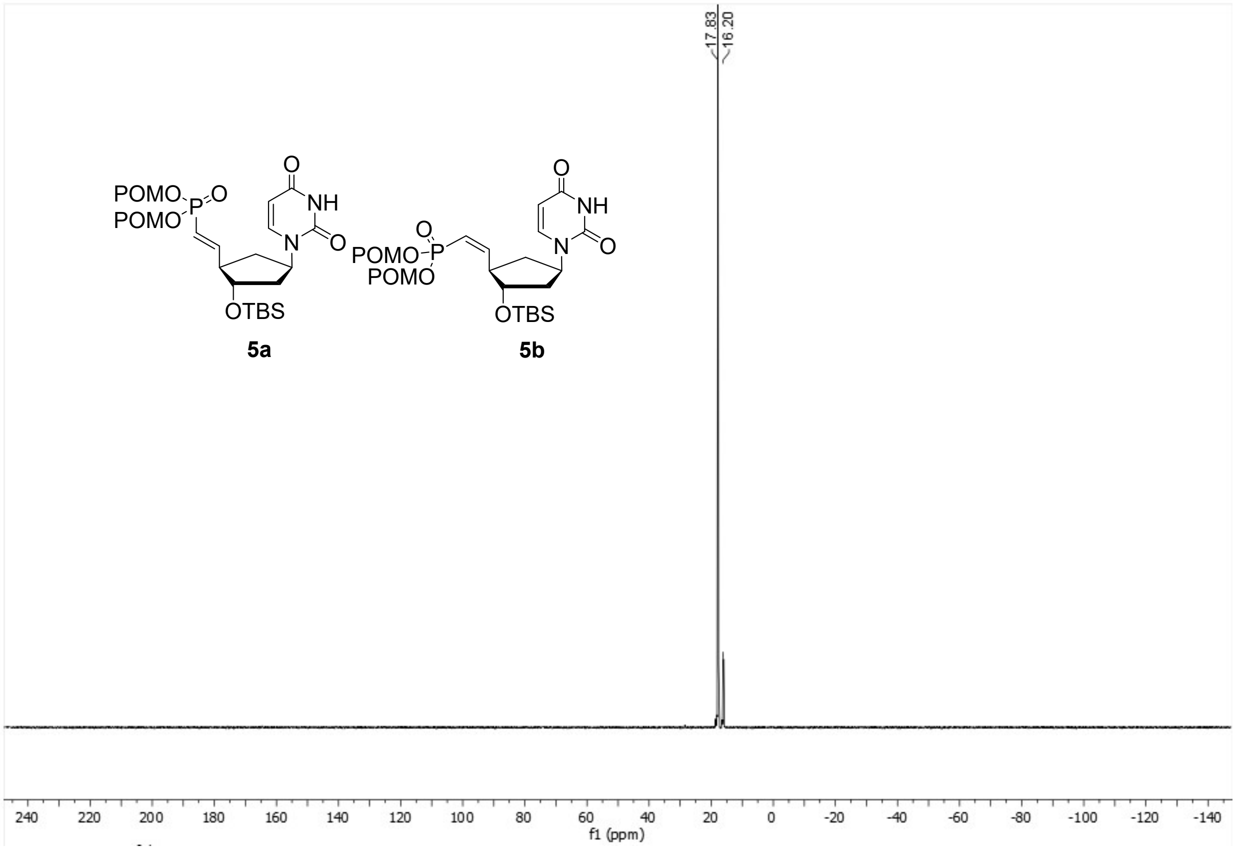

^{1}H NMR (600 MHz, DMSO-d_6) of compound **6a**

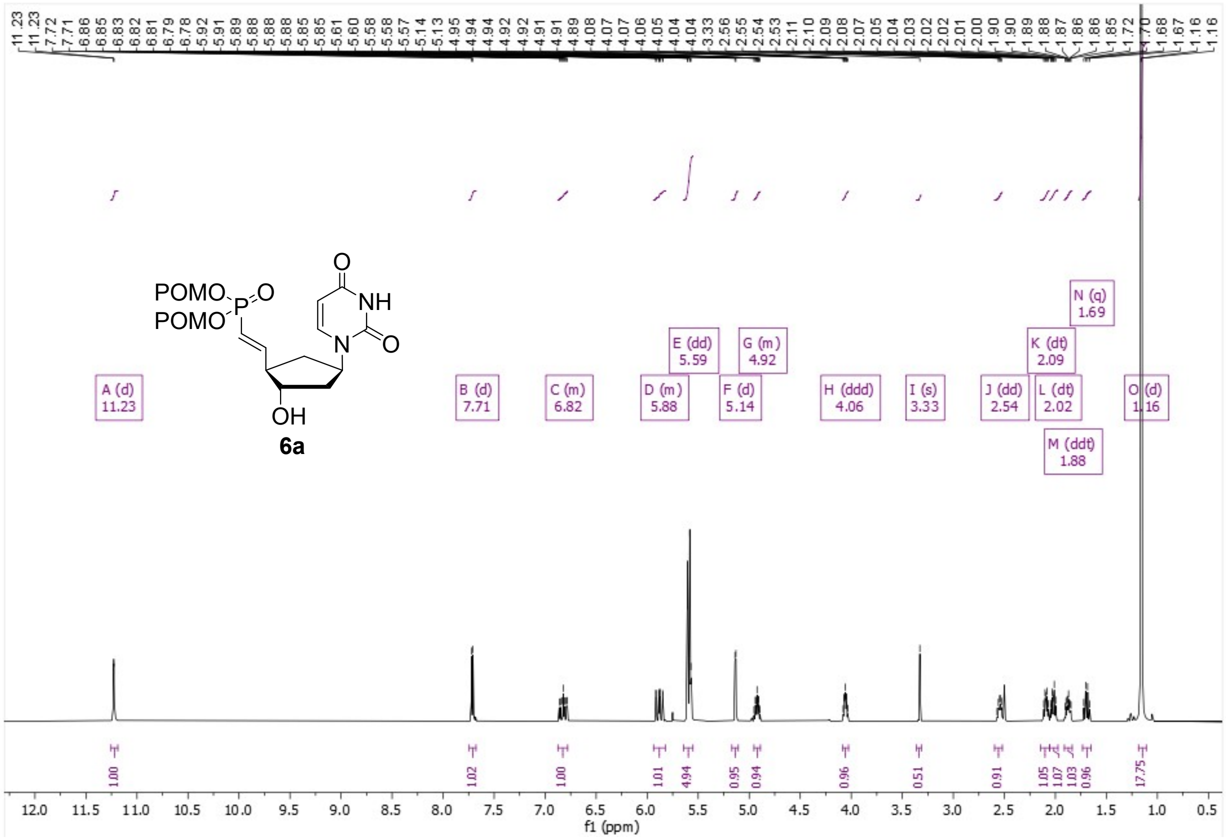

^{13}C NMR (151 MHz, DMSO-d_6) of compound **6a**

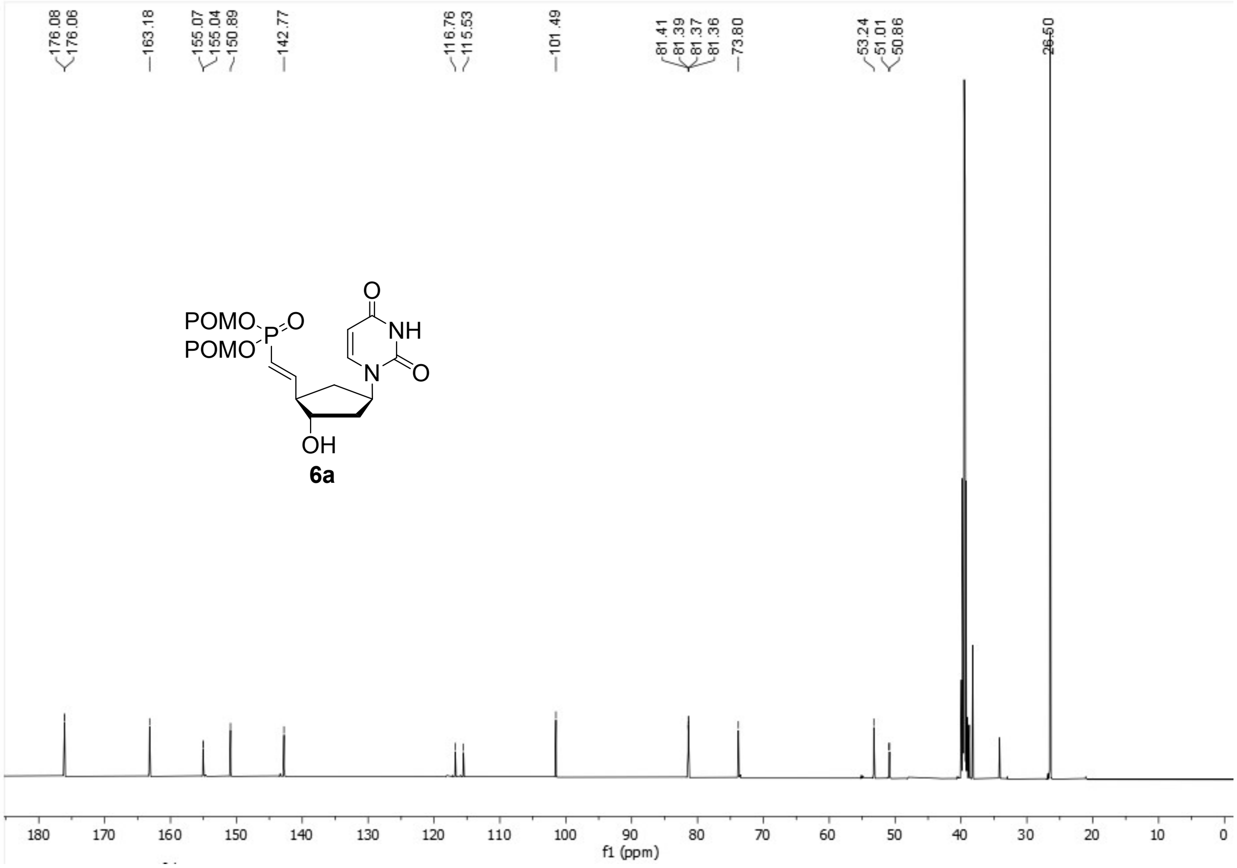

^{31}P NMR (243 MHz, DMSO-d_6) of compound **6a**

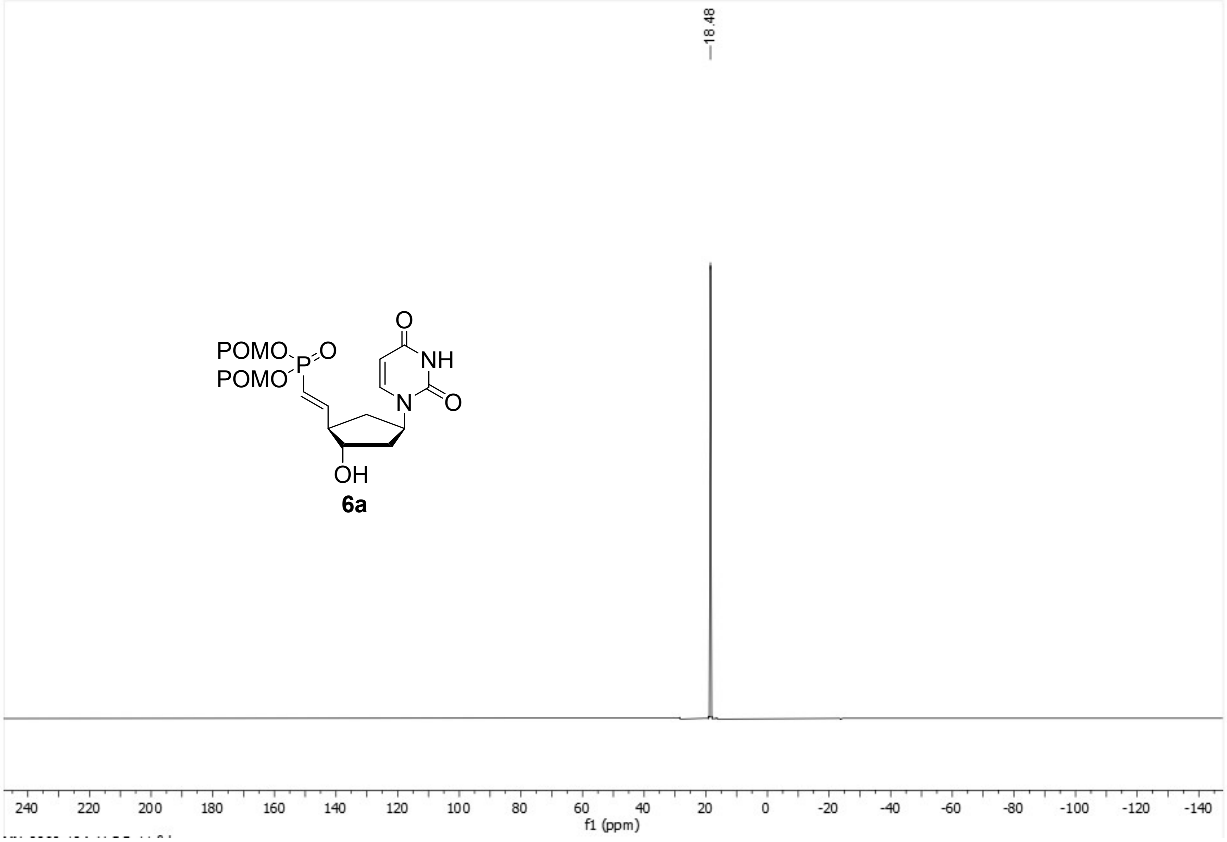

^{1}H NMR (600 MHz, DMSO-d_6) of compound **6b**

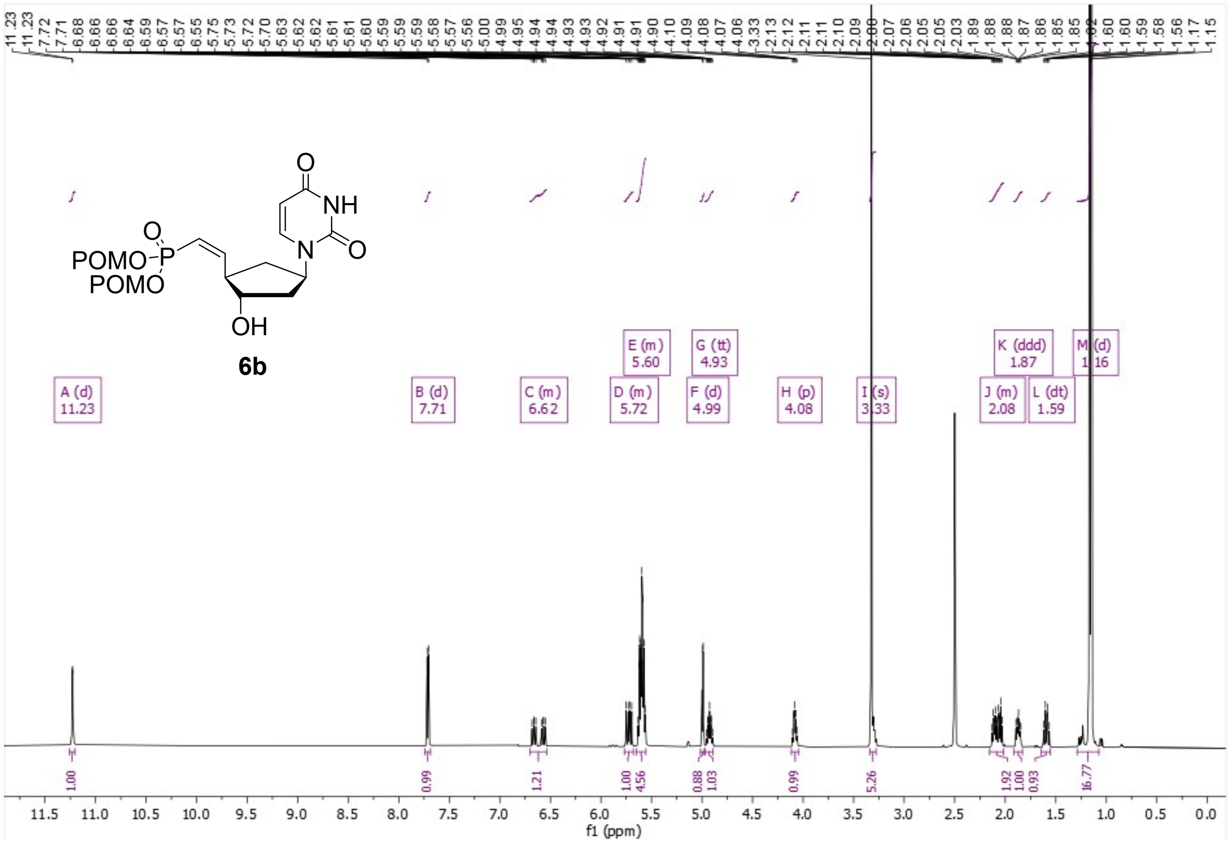

^{13}C NMR (151 MHz, DMSO-d_6) of compound **6b**

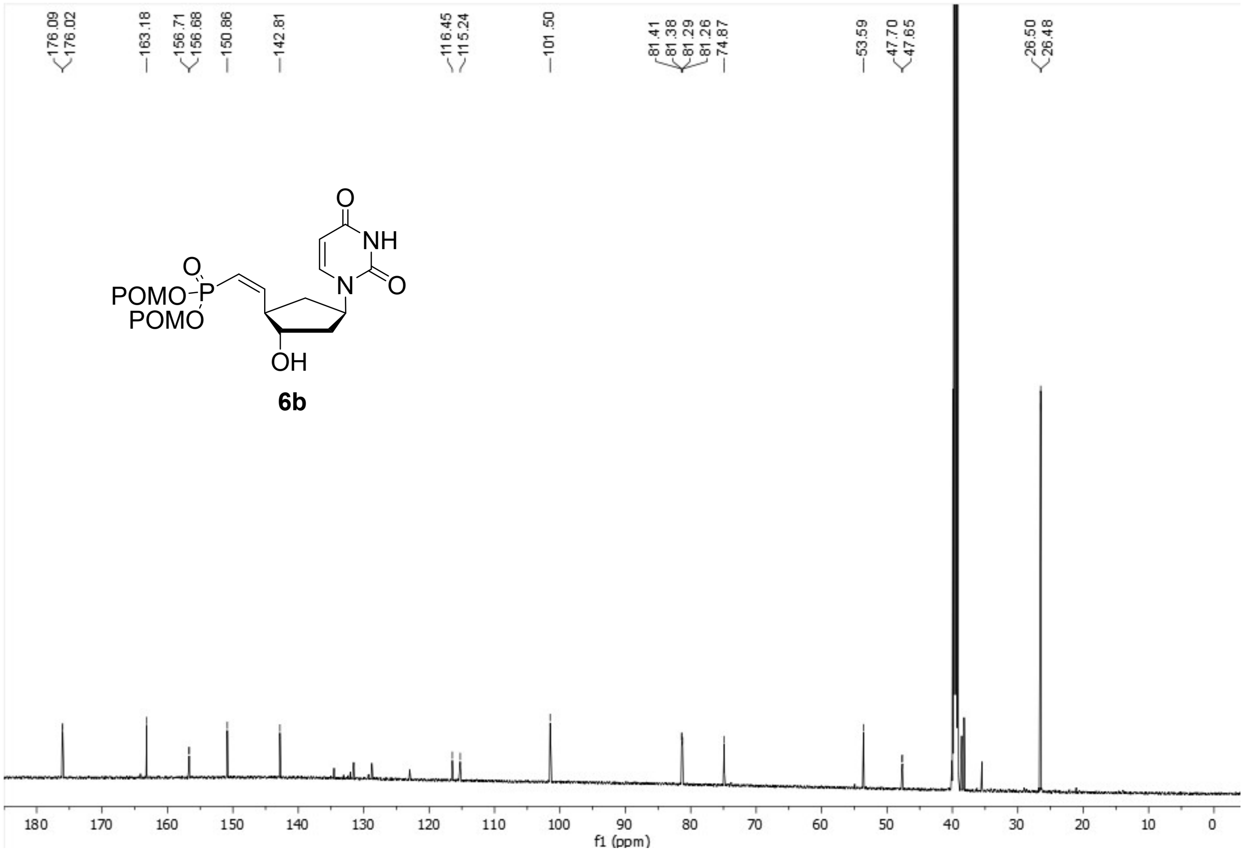

^{31}P NMR (243 MHz, DMSO-d_6) of compound **6b**

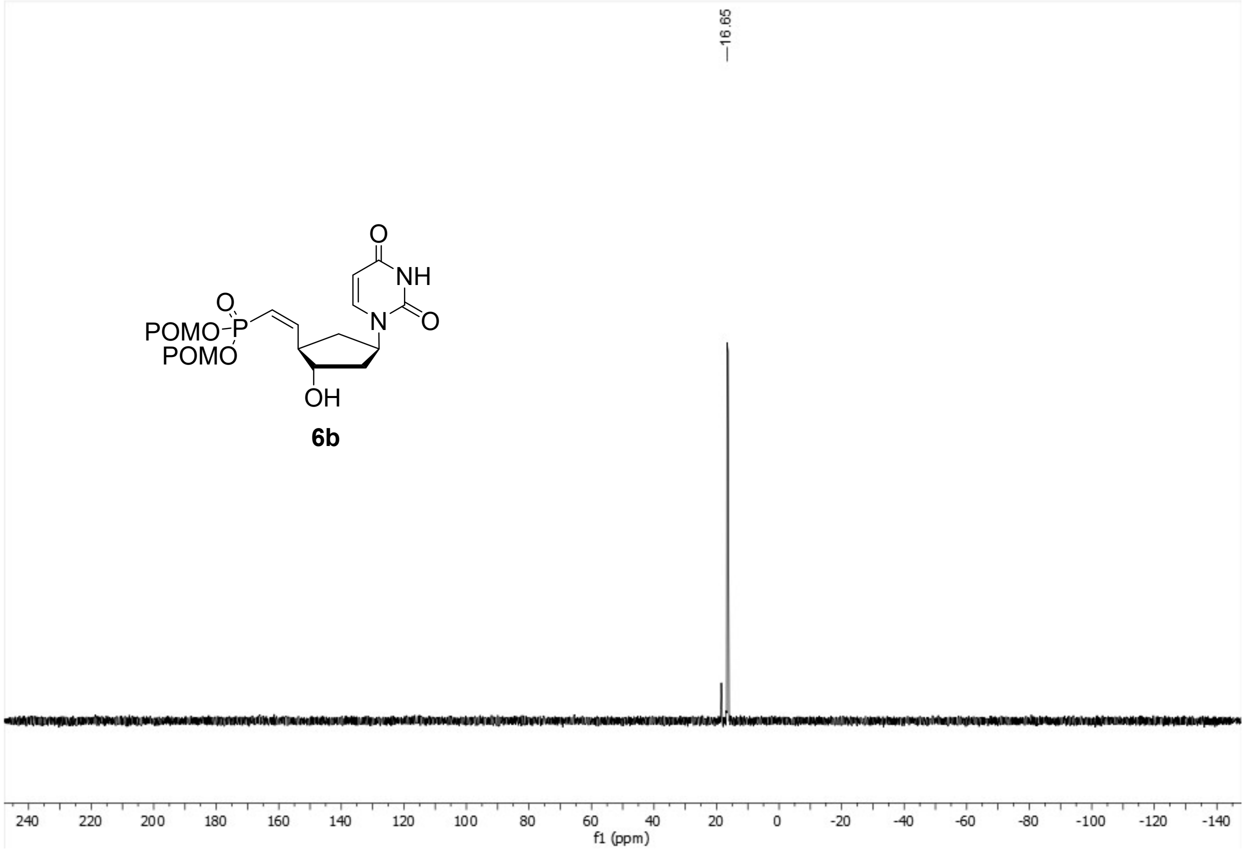

^{1}H NMR (600 MHz, CD_3CN) of compound **7a**

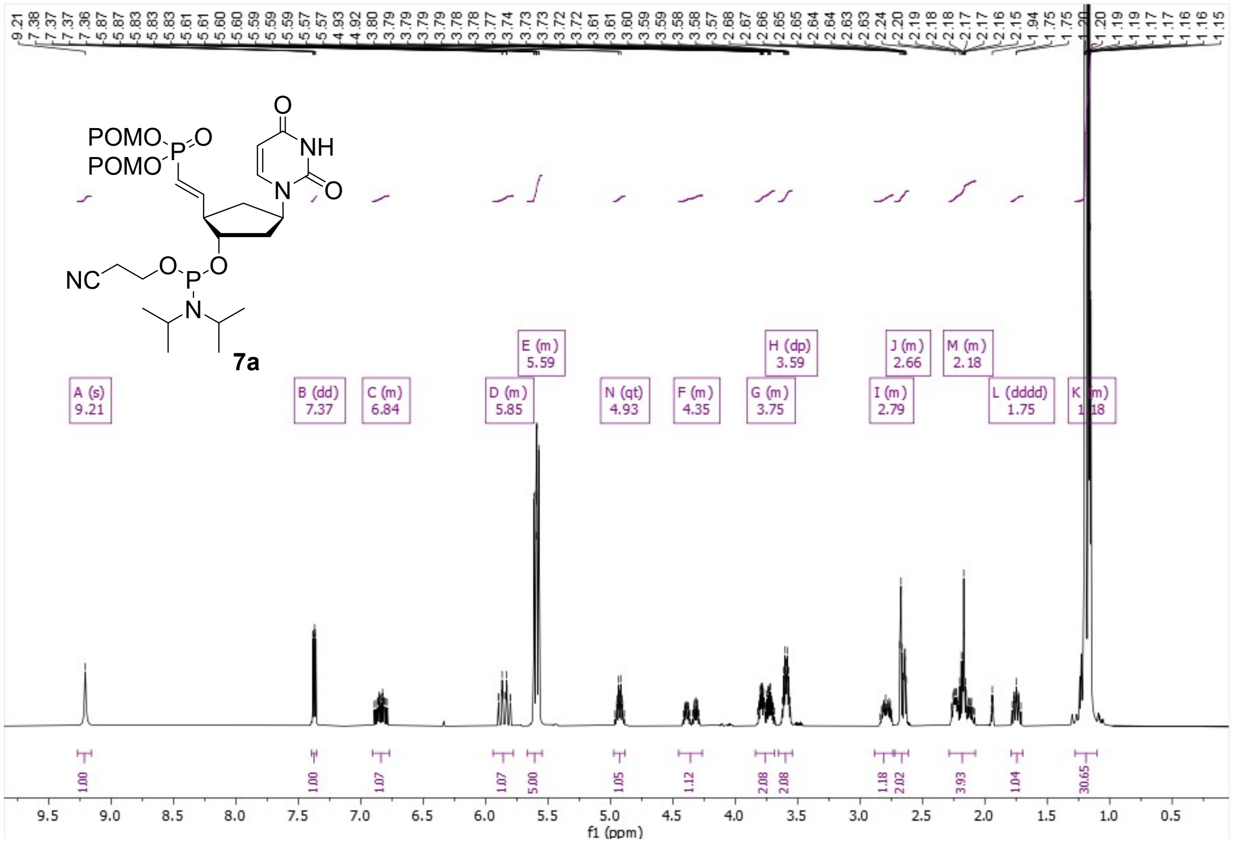

^{13}C NMR (151 MHz, CD_3CN) of compound **7a**

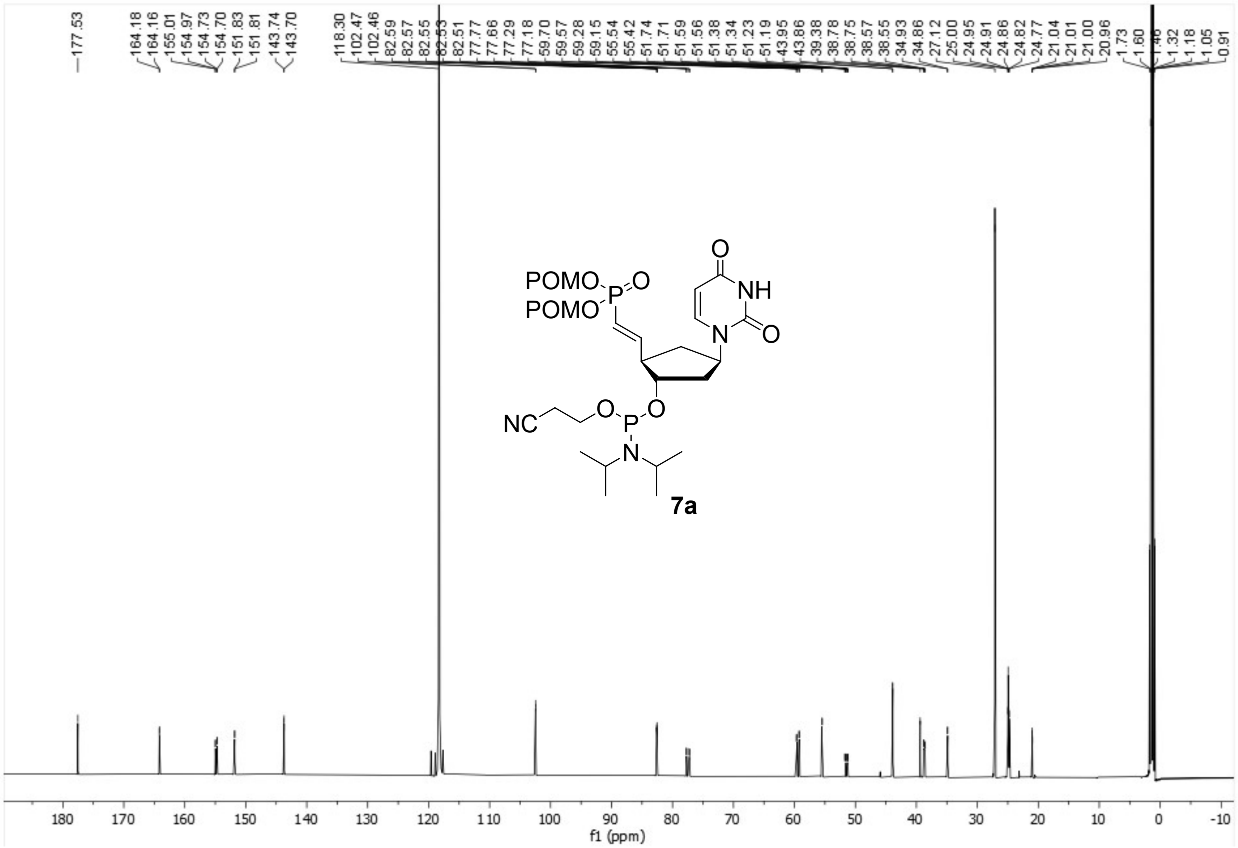

^{31}P NMR (243 MHz, CD_3CN) of compound **7a**

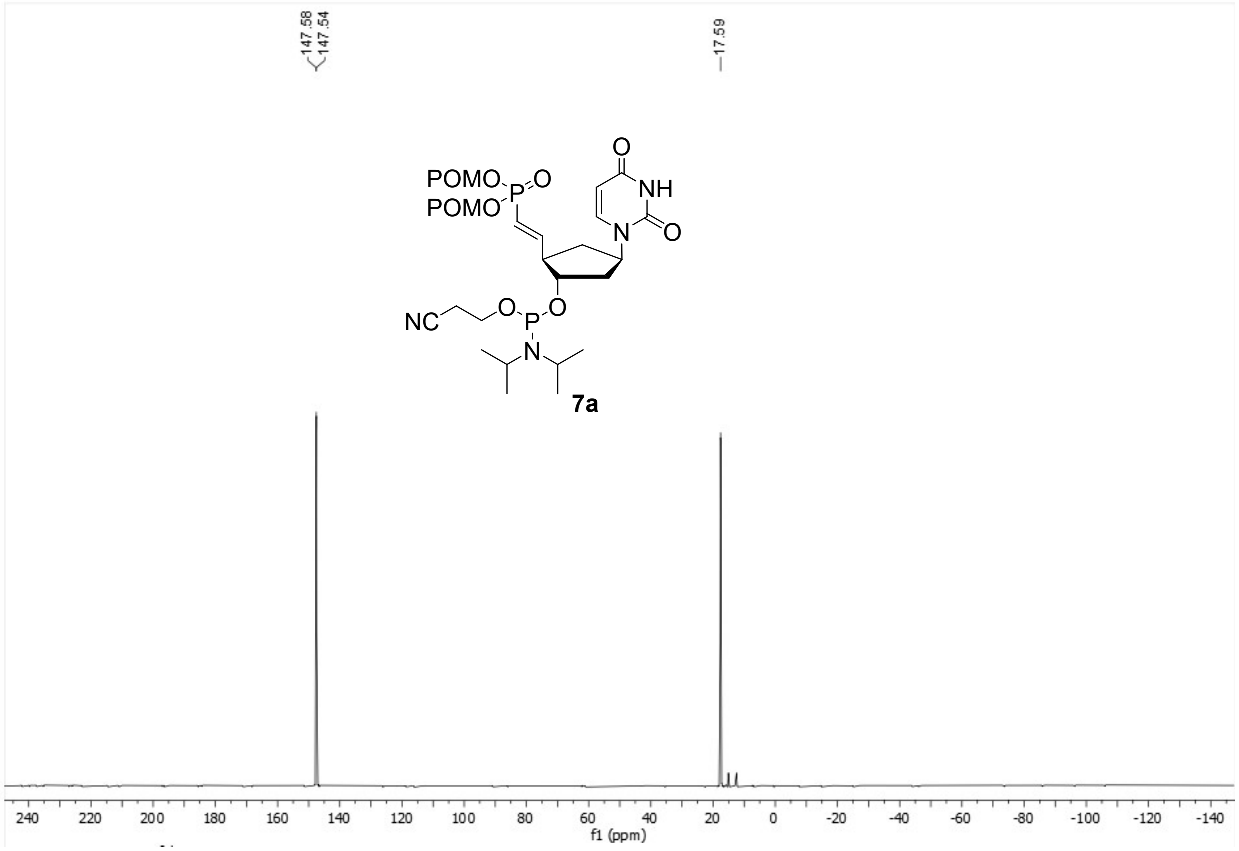

^{1}H NMR (600 MHz, DMSO-d_6) of compound **9**

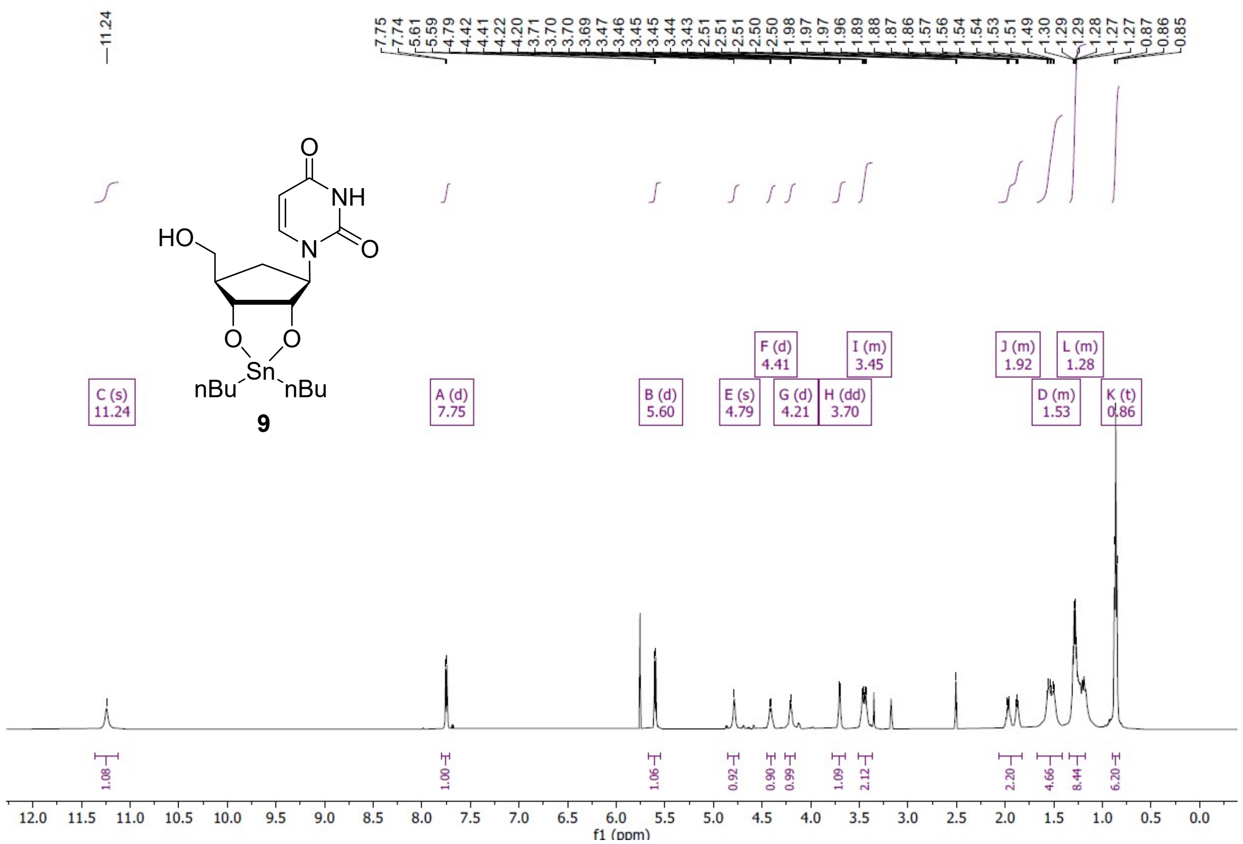

^{13}C NMR (151 MHz, DMSO-d_6) of compound **9**

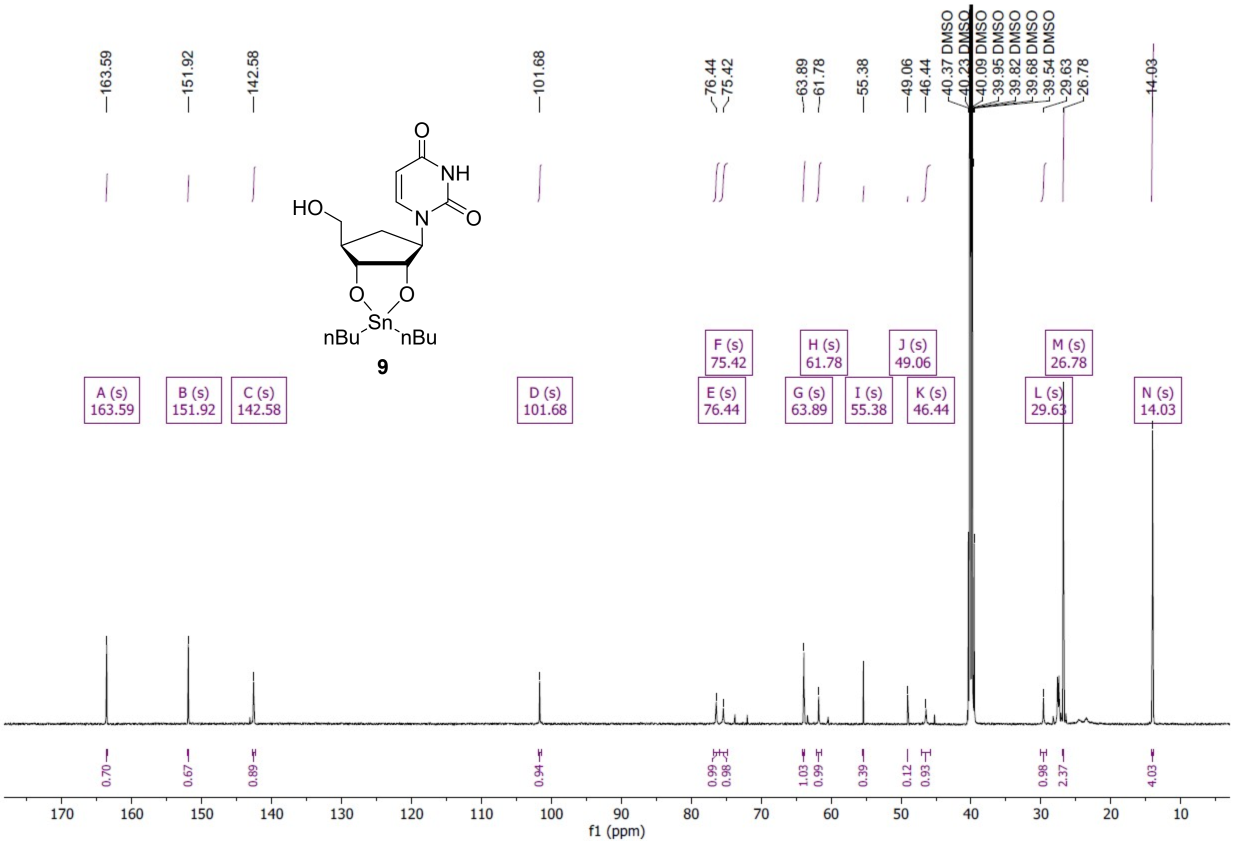

^{1}H NMR (600 MHz, DMSO-d_6) of compound **10** and **11**

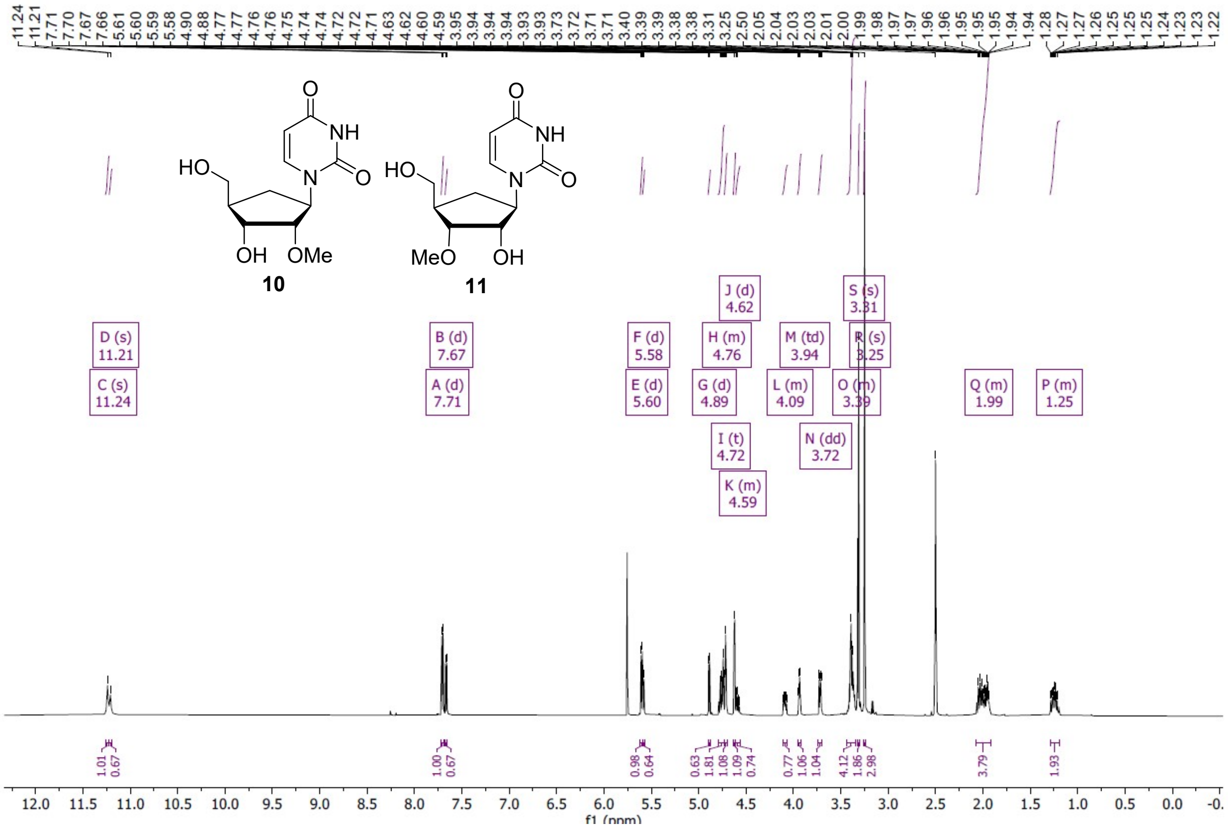

^{13}C NMR (151 MHz, DMSO-d_6) of compound **10** and **11**

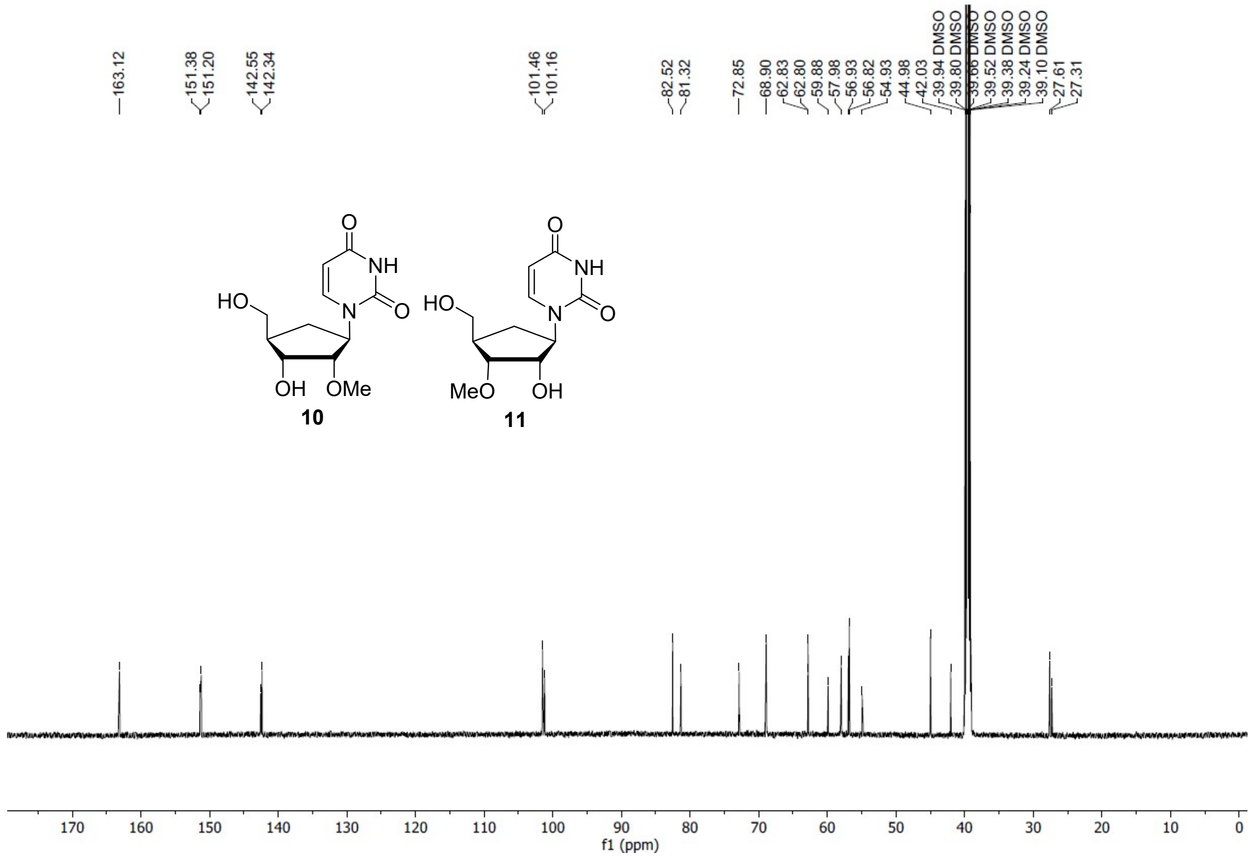

^{1}H NMR (600 MHz, DMSO-d_6) of compound **12**

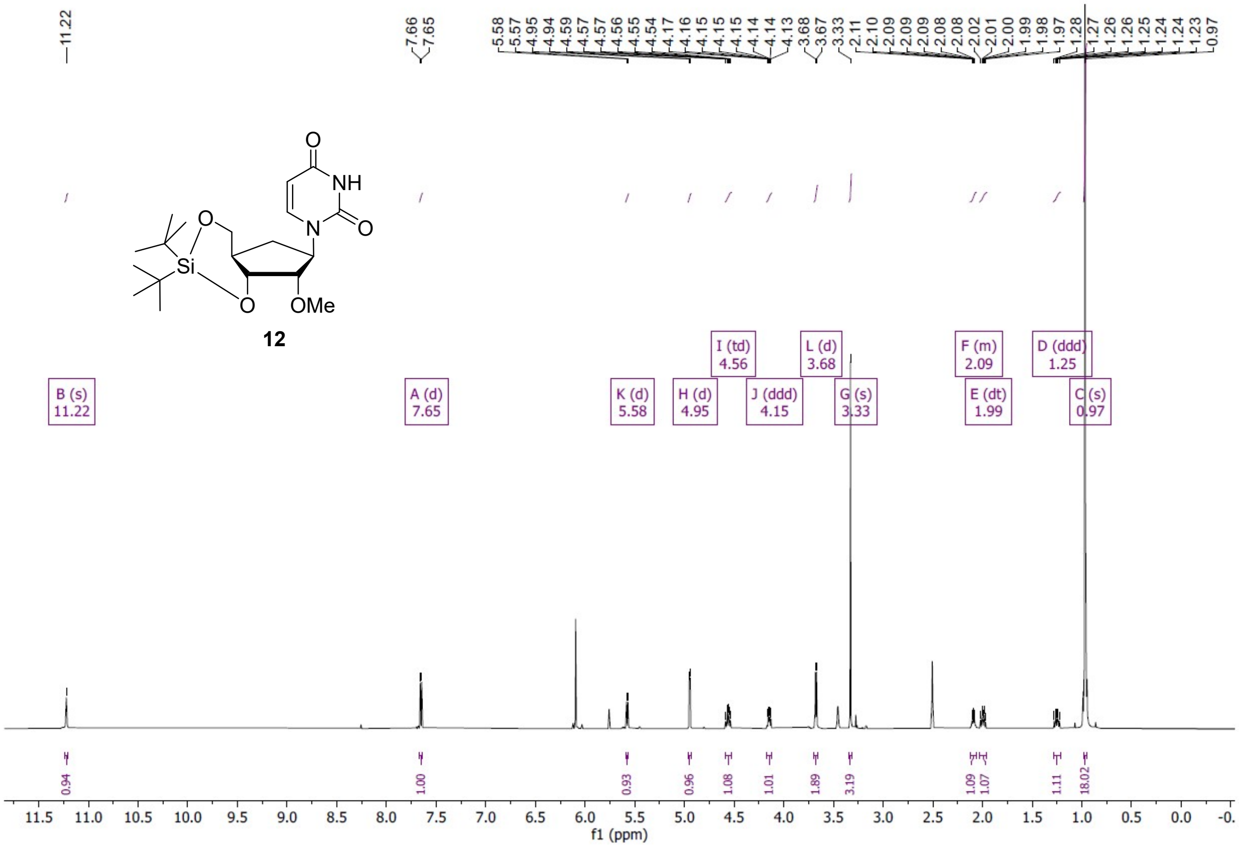

^{13}C NMR (151 MHz, DMSO-d_6) of compound **12**

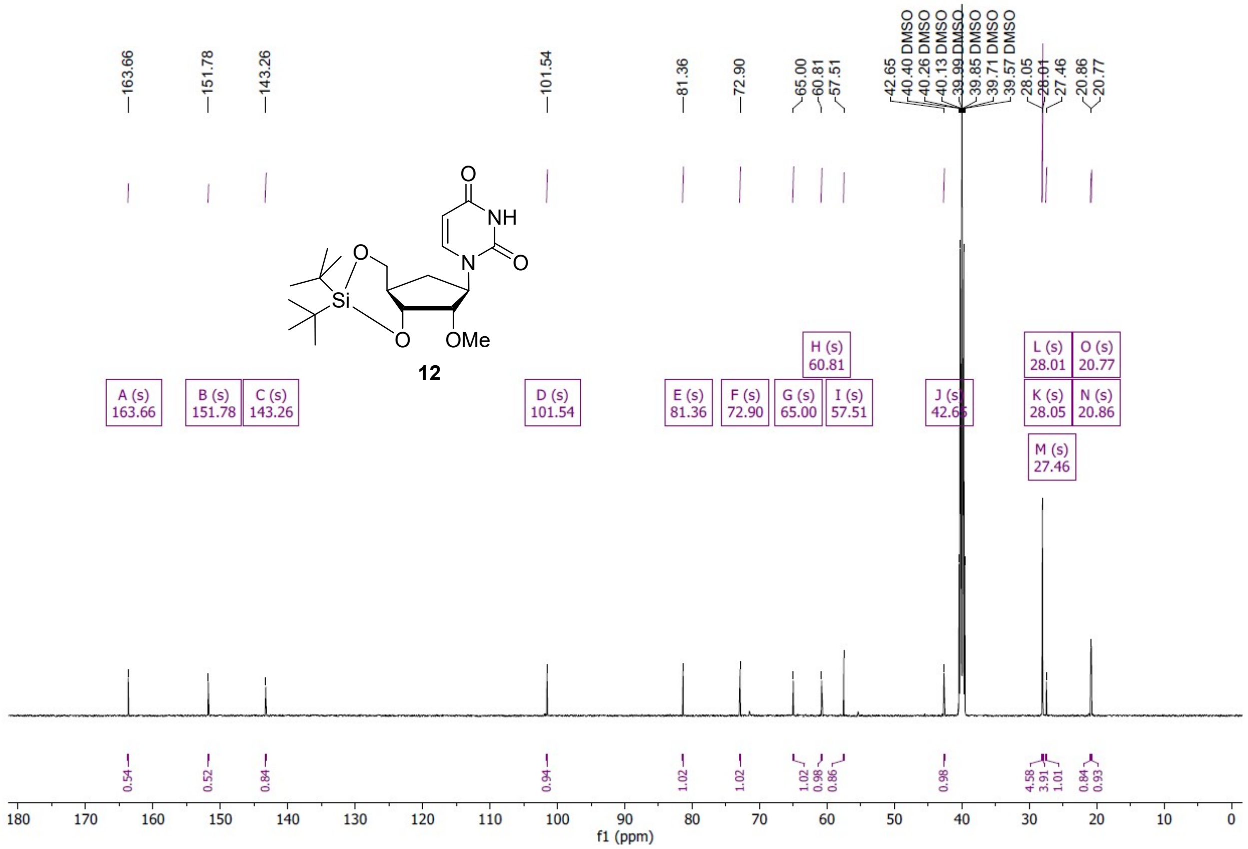

^{1}H NMR (600 MHz, DMSO-d_6) of compound **13**

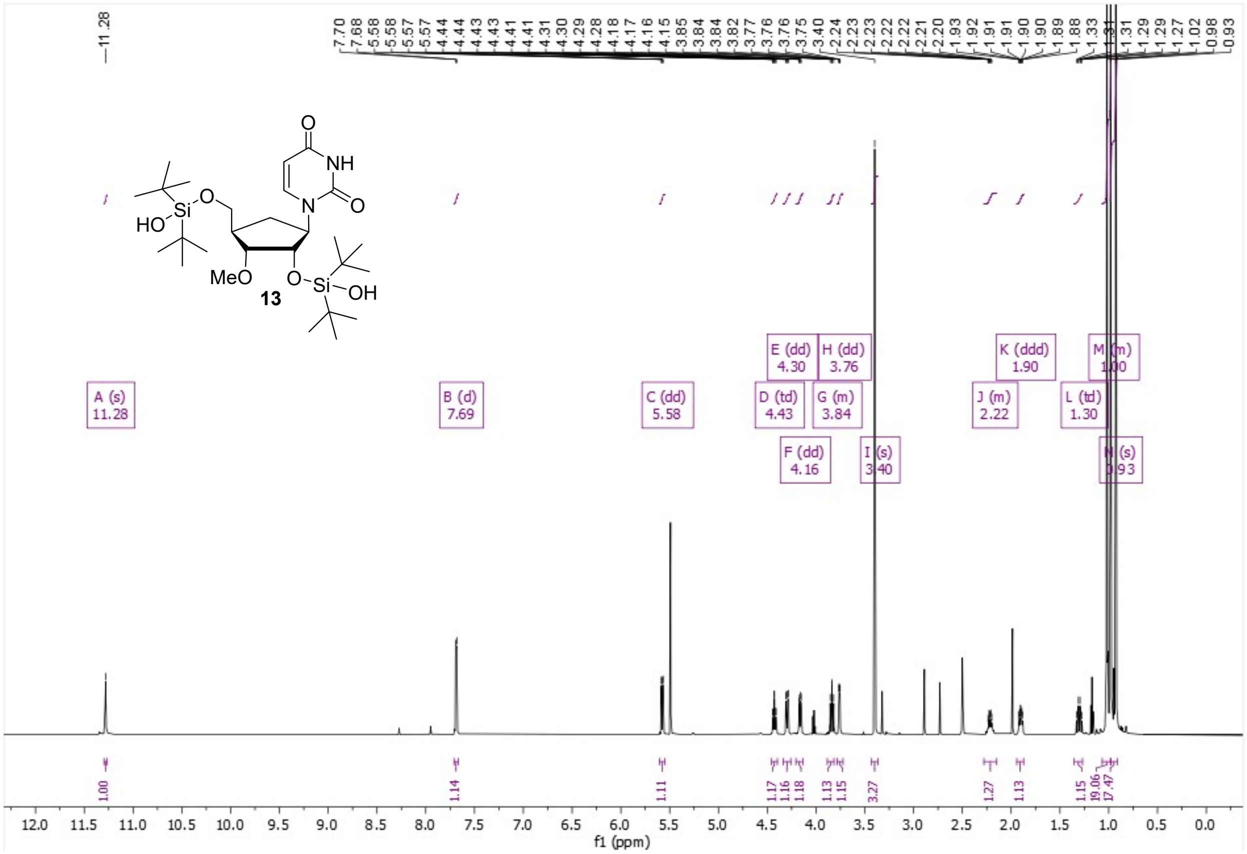

^{13}C NMR (151 MHz, DMSO-d_6) of compound **13**

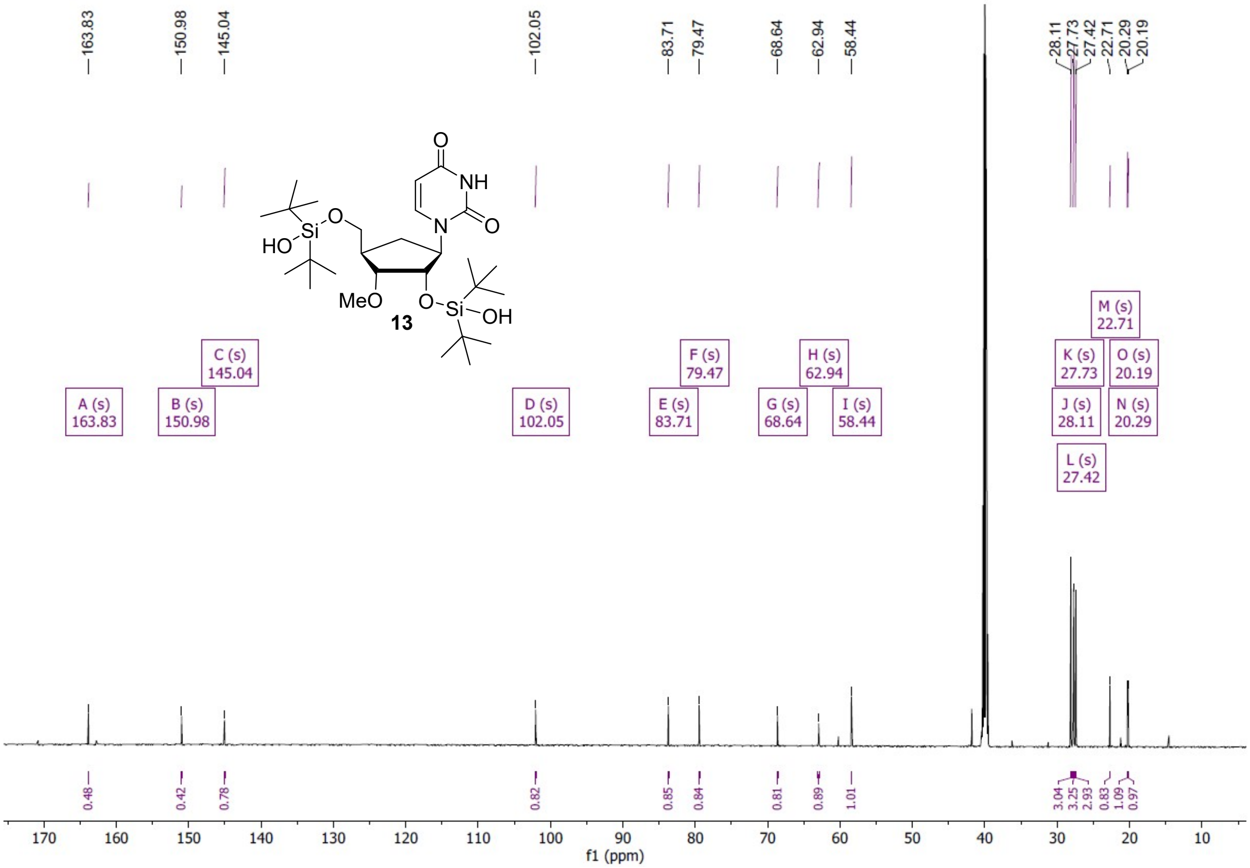

^{1}H NMR (600 MHz, DMSO-d_6) of compound **10**

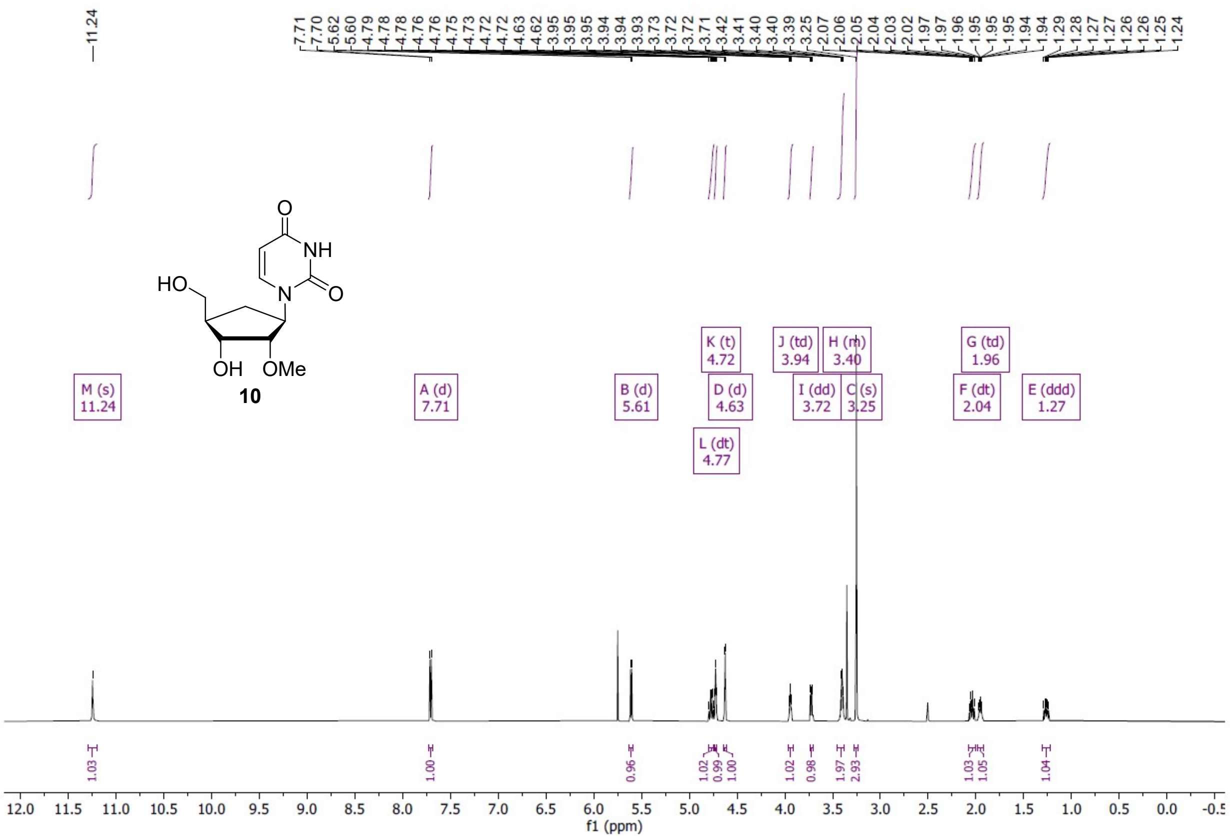

^{13}C NMR (151 MHz, DMSO-d_6) of compound **10**

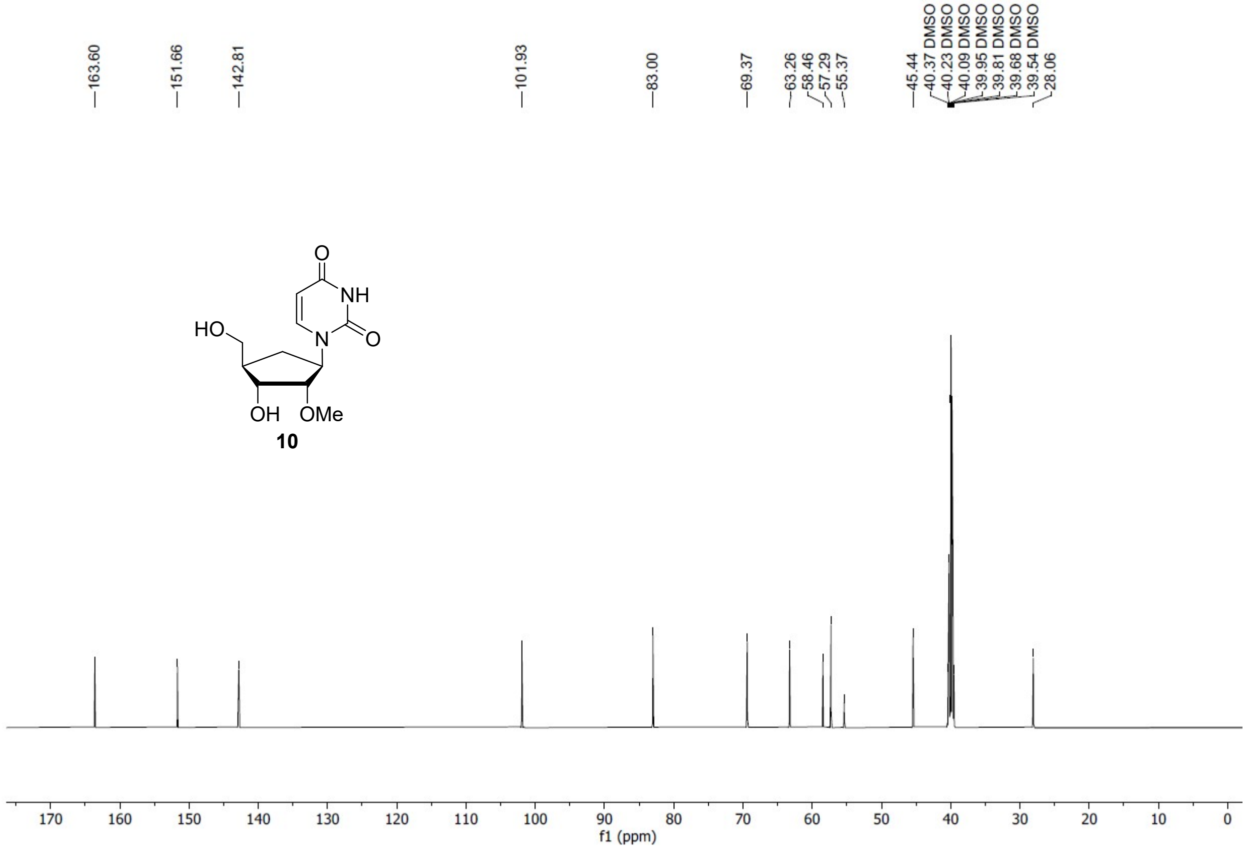

^{1}H NMR (600 MHz, DMSO-d_6) of compound **11**

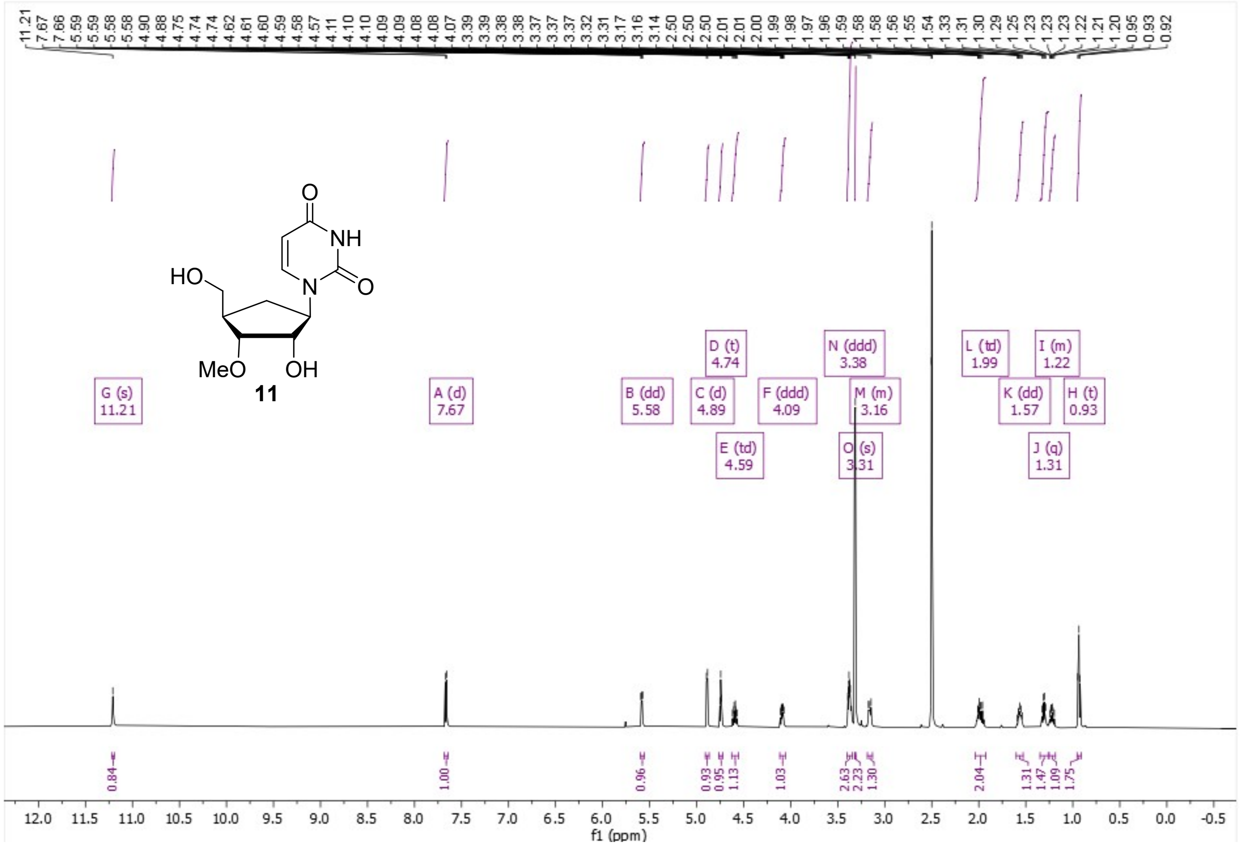

^{13}C NMR (151 MHz, DMSO-d_6) of compound **11**

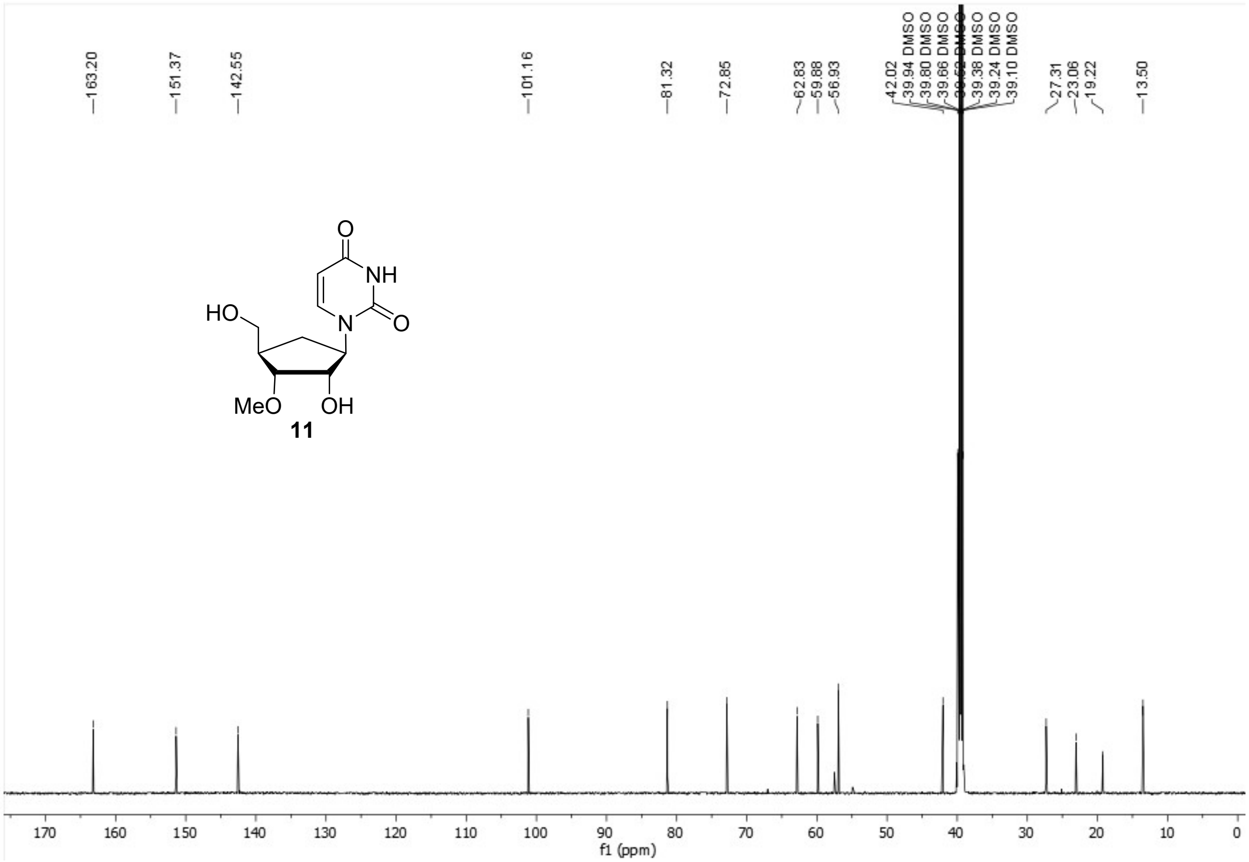

^{1}H NMR (600 MHz, CD_3CN) of compound **14**

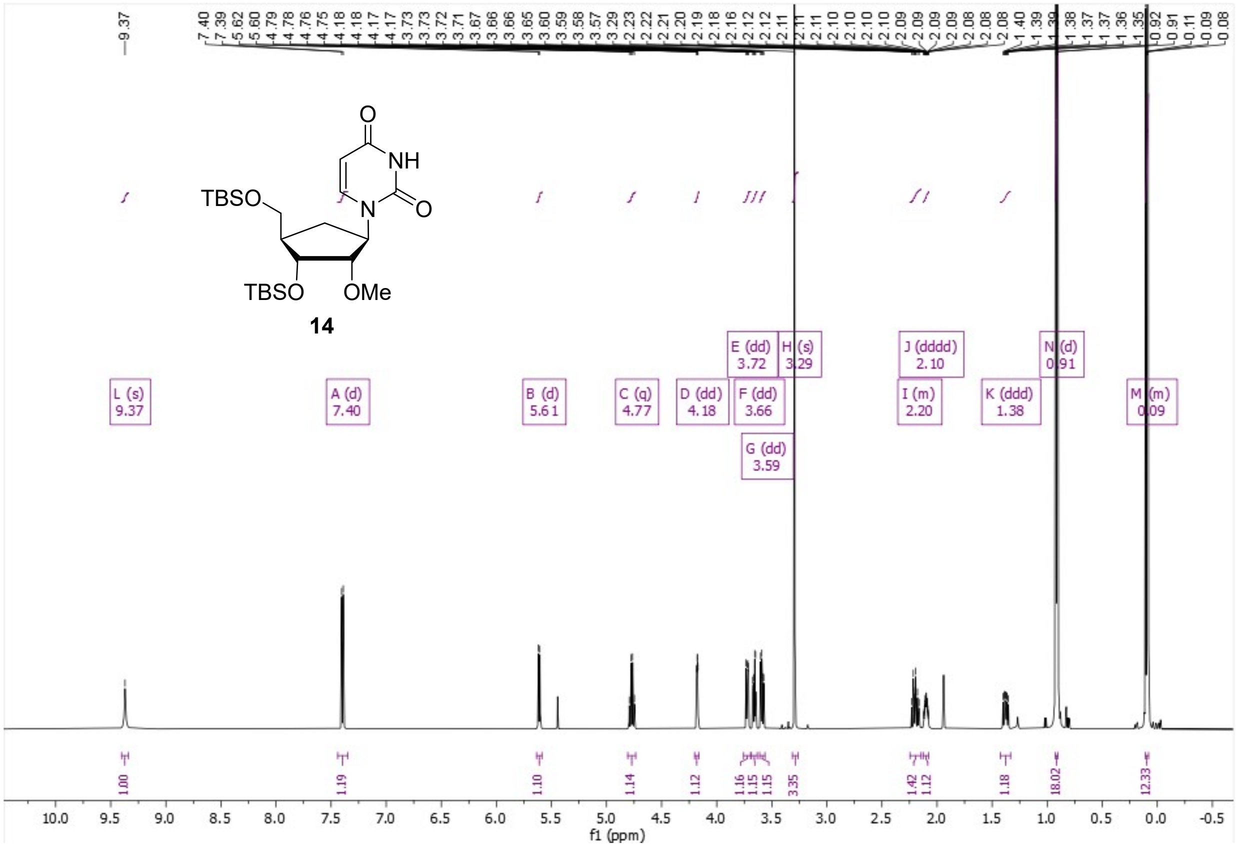

^{13}C NMR (151 MHz, CD_3CN) of compound **14**

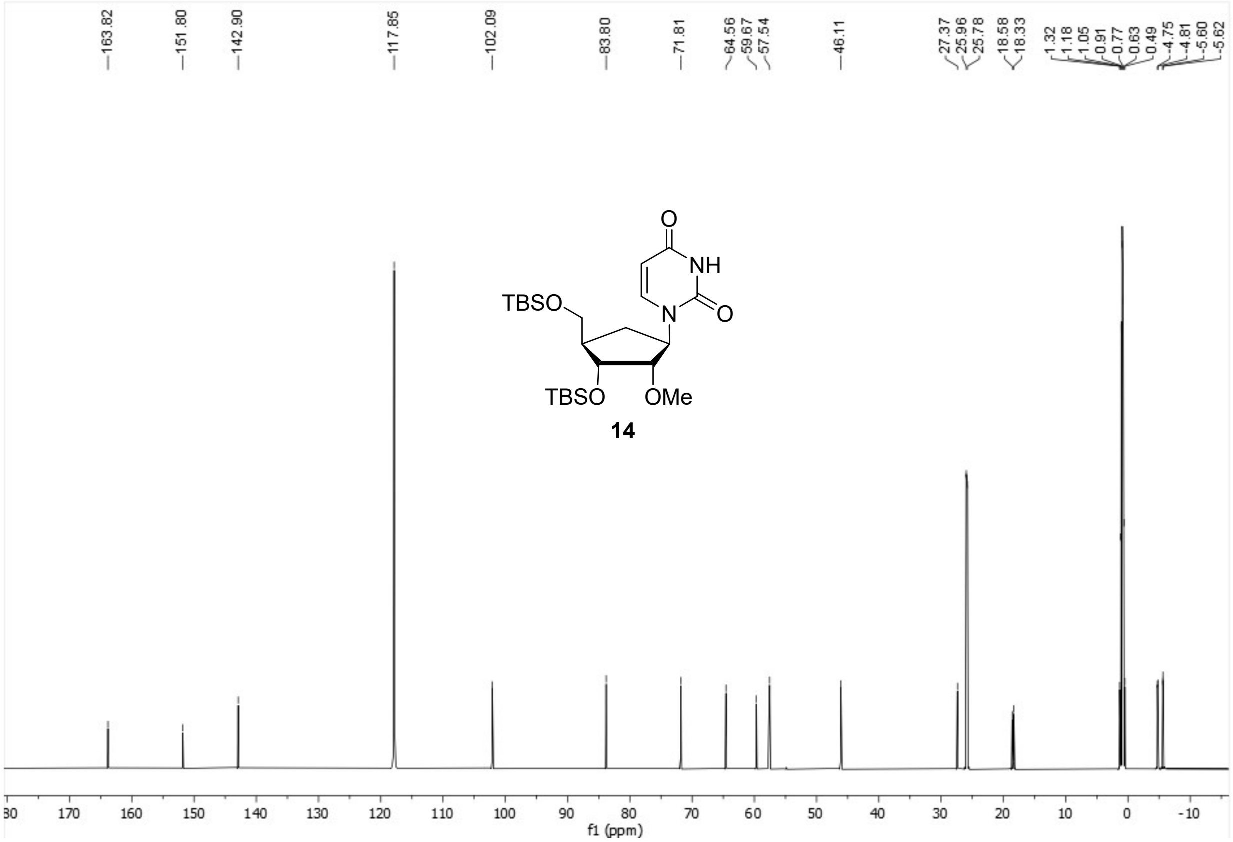

^1H NMR (600 MHz, CD_2Cl_2) of compound **15**

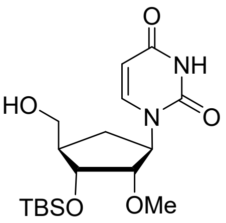

15

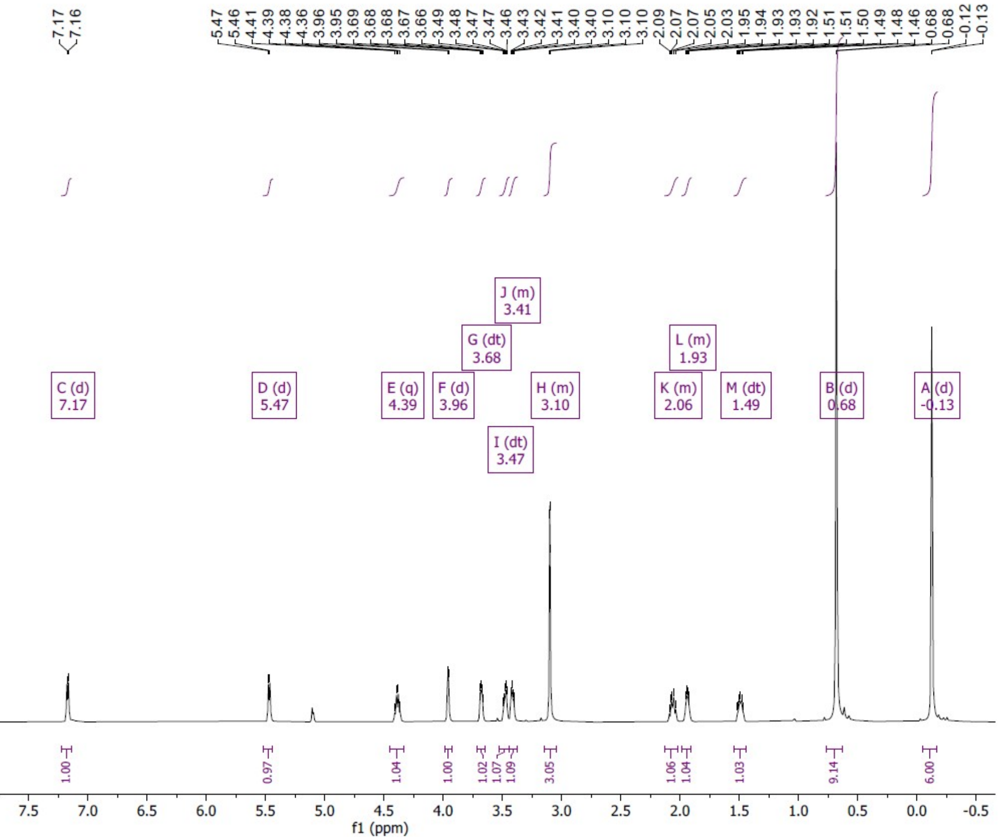

^{13}C NMR (151 MHz, CD_2Cl_2) of compound **15**

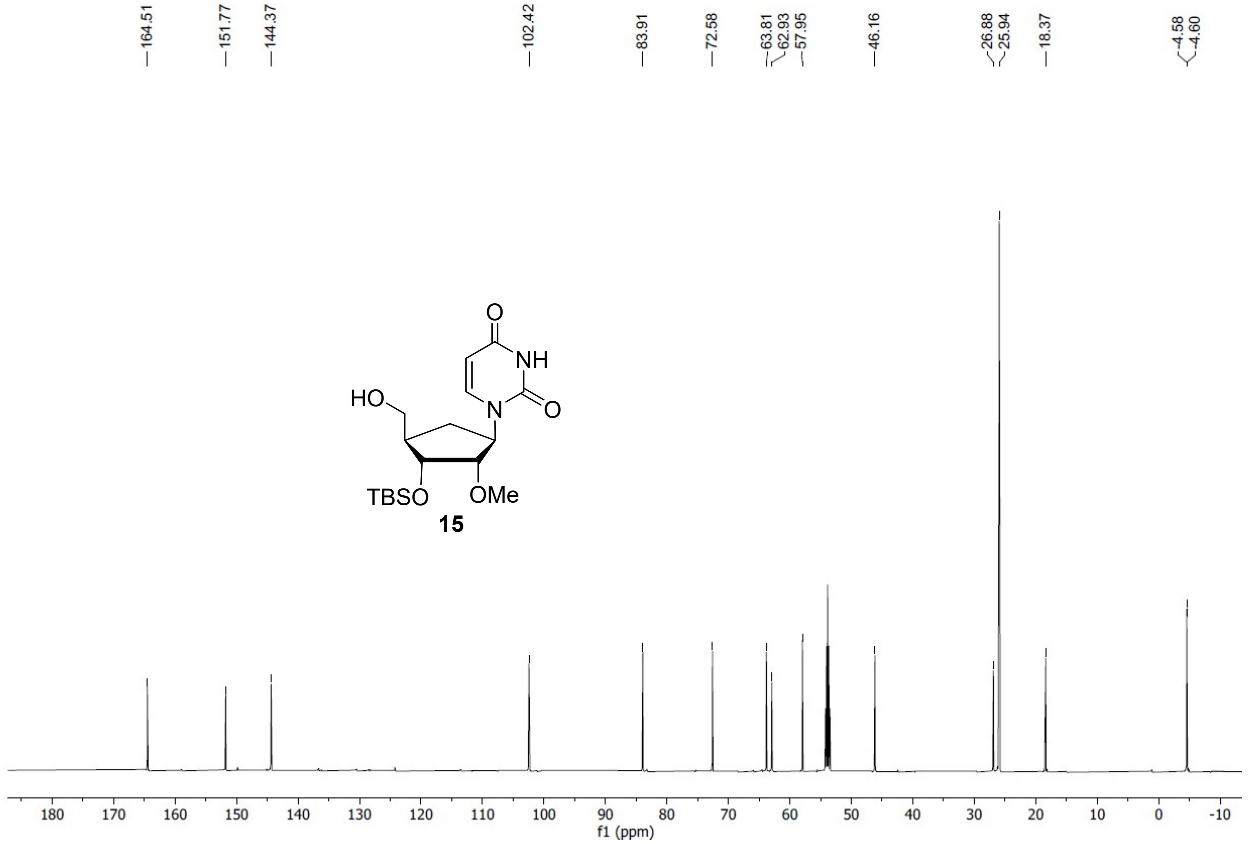

^{1}H NMR (600 MHz, CD_3CN) of compound **18**

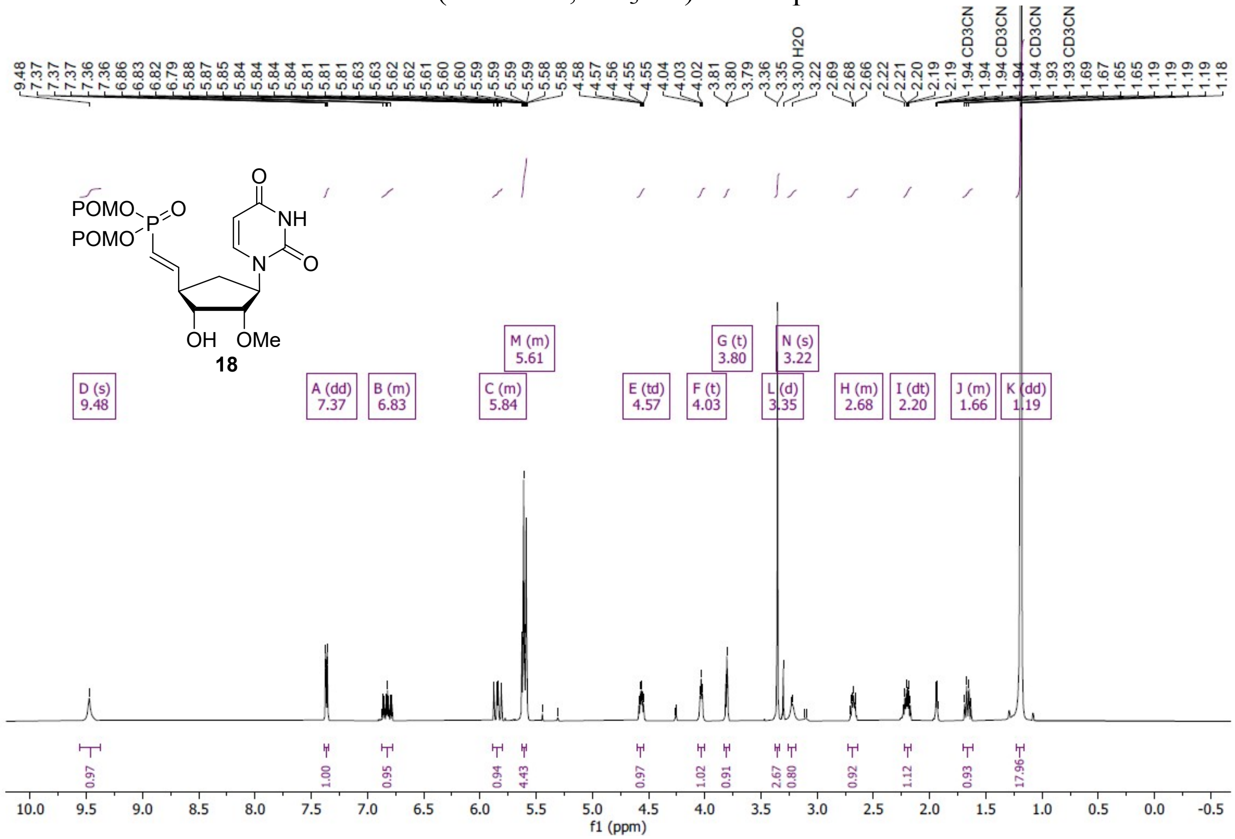

^{13}C NMR (151 MHz, CD_3CN) of compound **18**

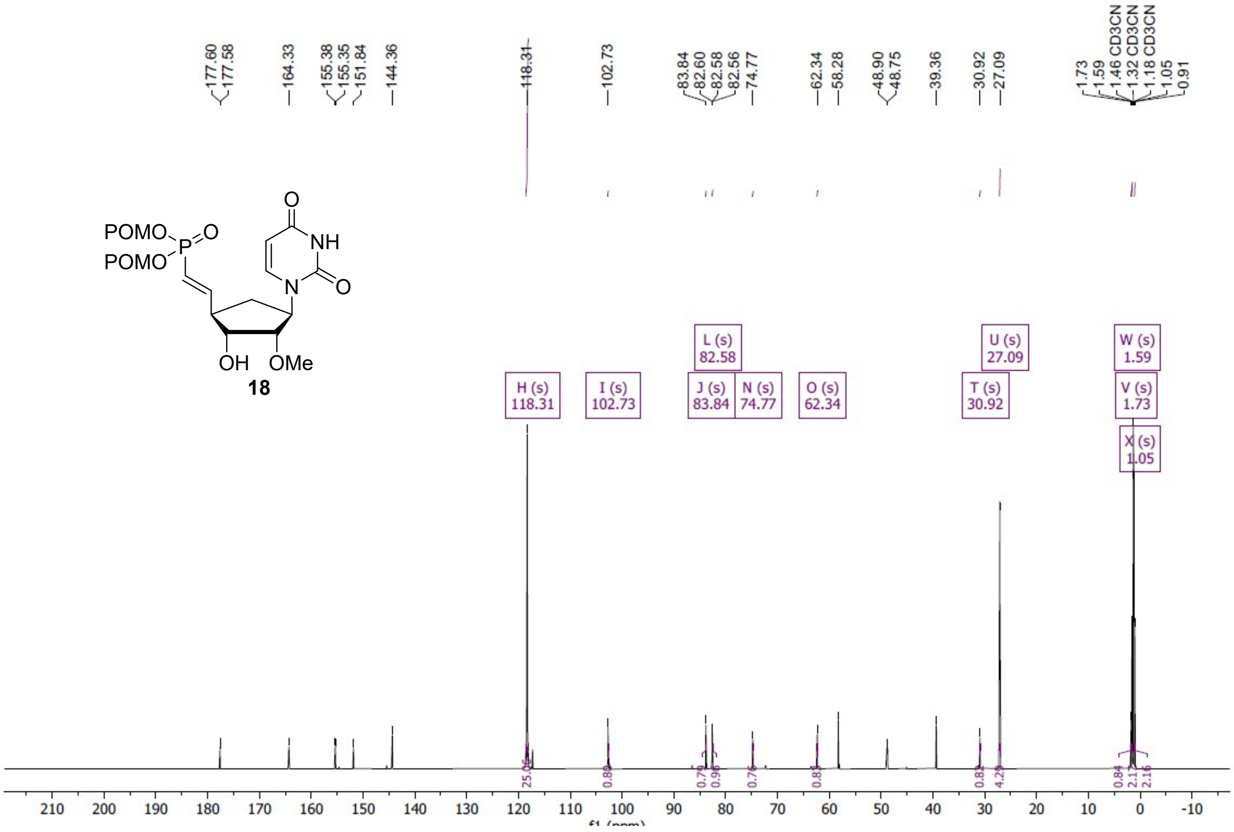

^{31}P NMR (243 MHz, CD_3CN) of compound **18**

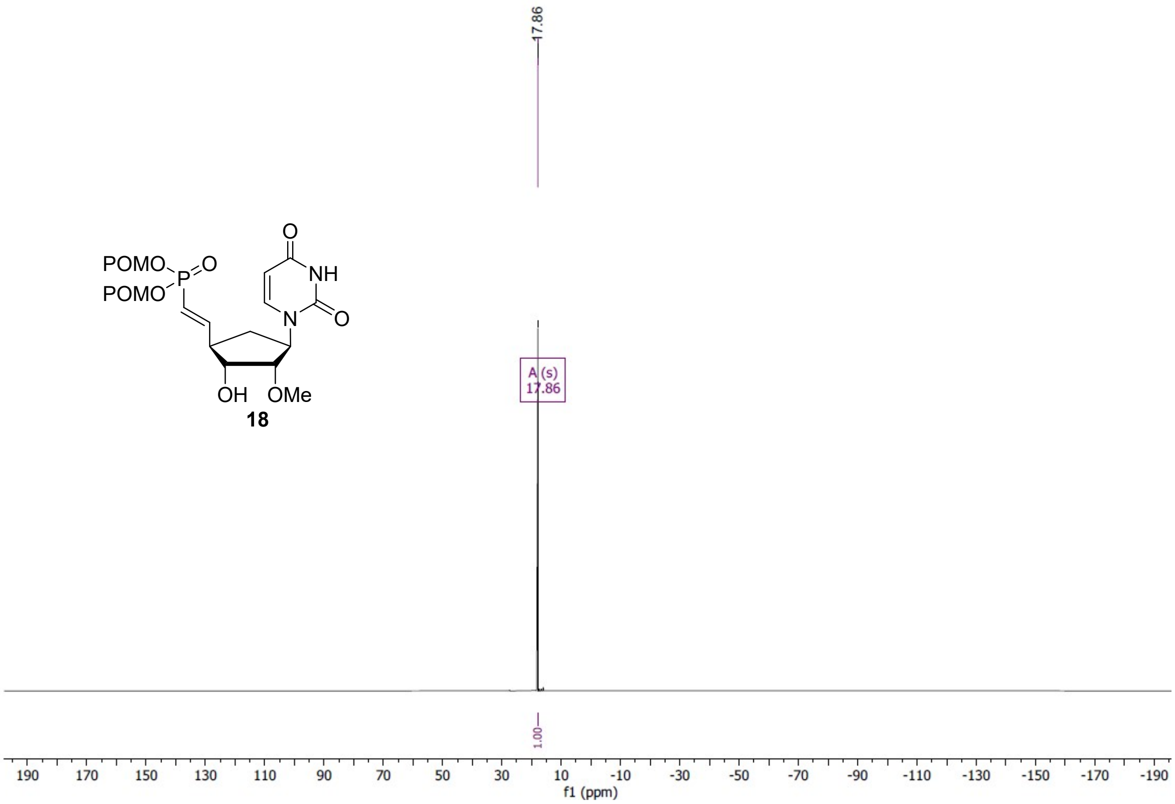

^{31}P NMR (243 MHz, CD_3CN) of compound (**E-Z mixture**)

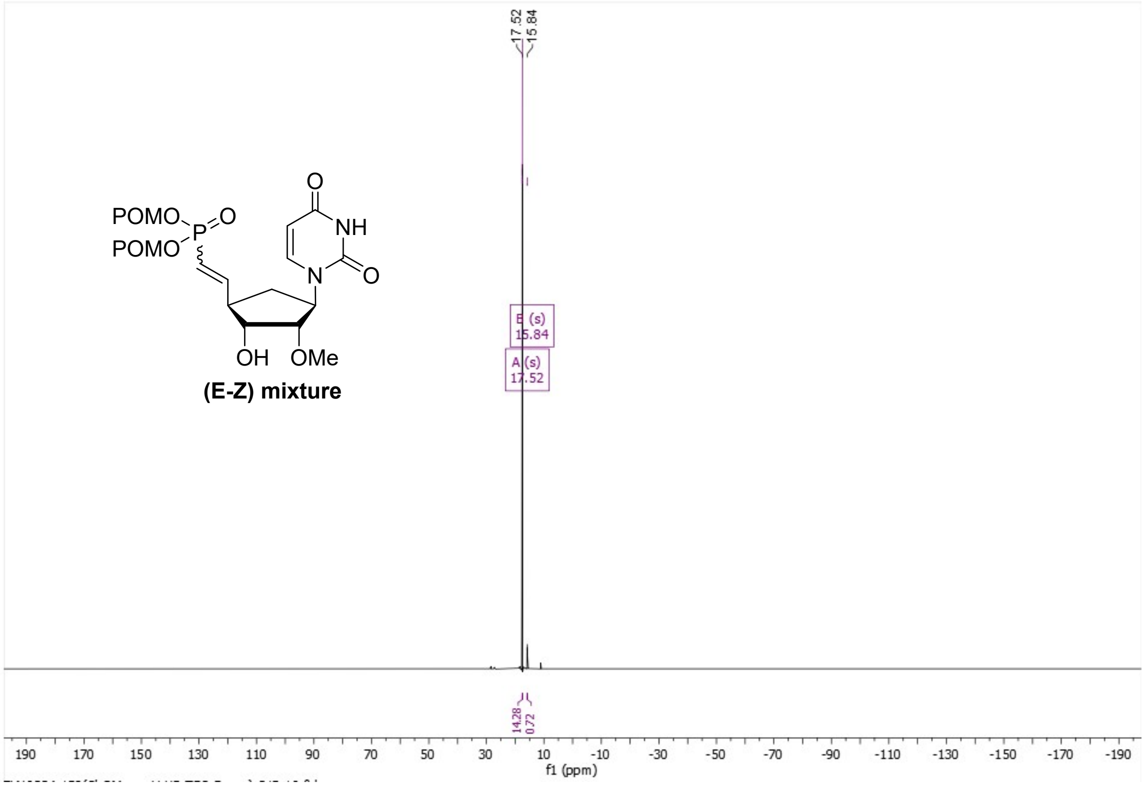

^{1}H NMR (600 MHz, CD_3CN) of compound **19**

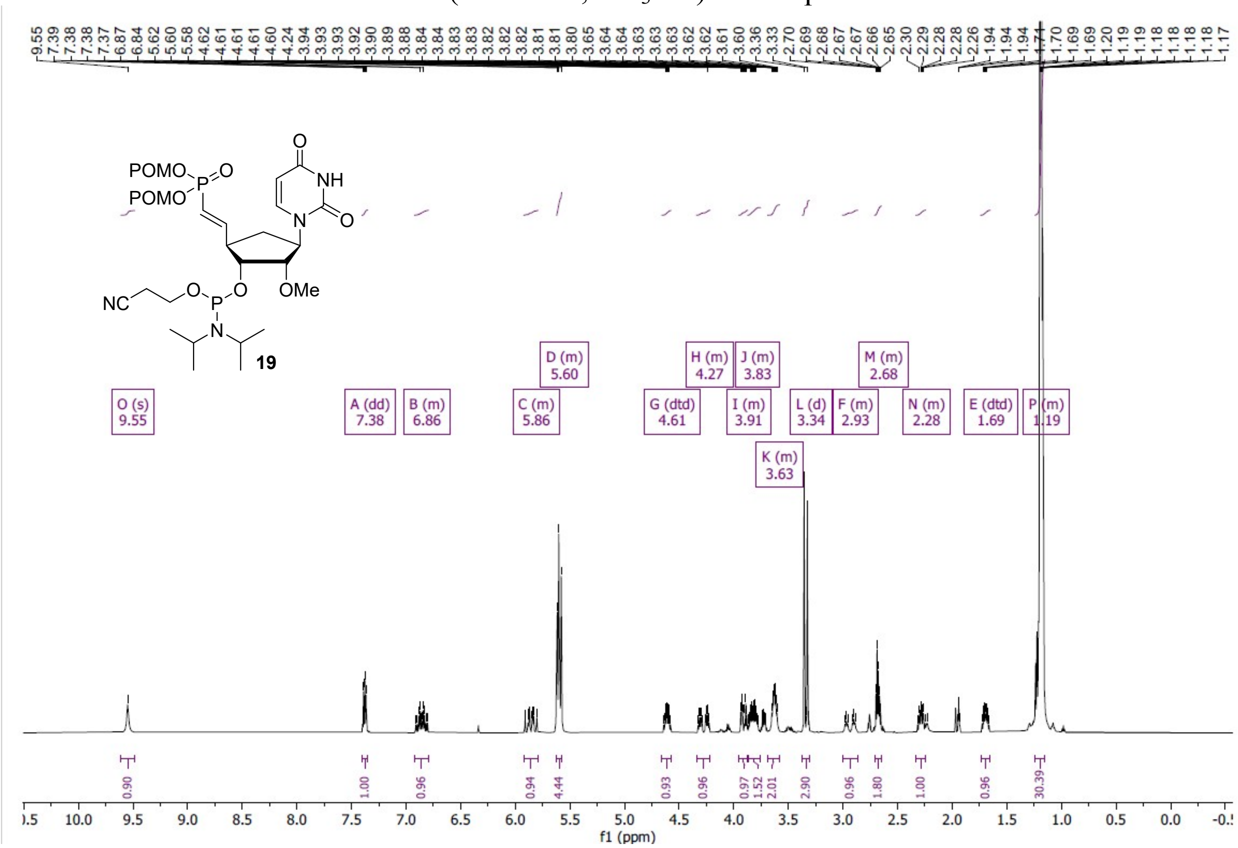

^{13}C NMR (151 MHz, CD_3CN) of compound **19**

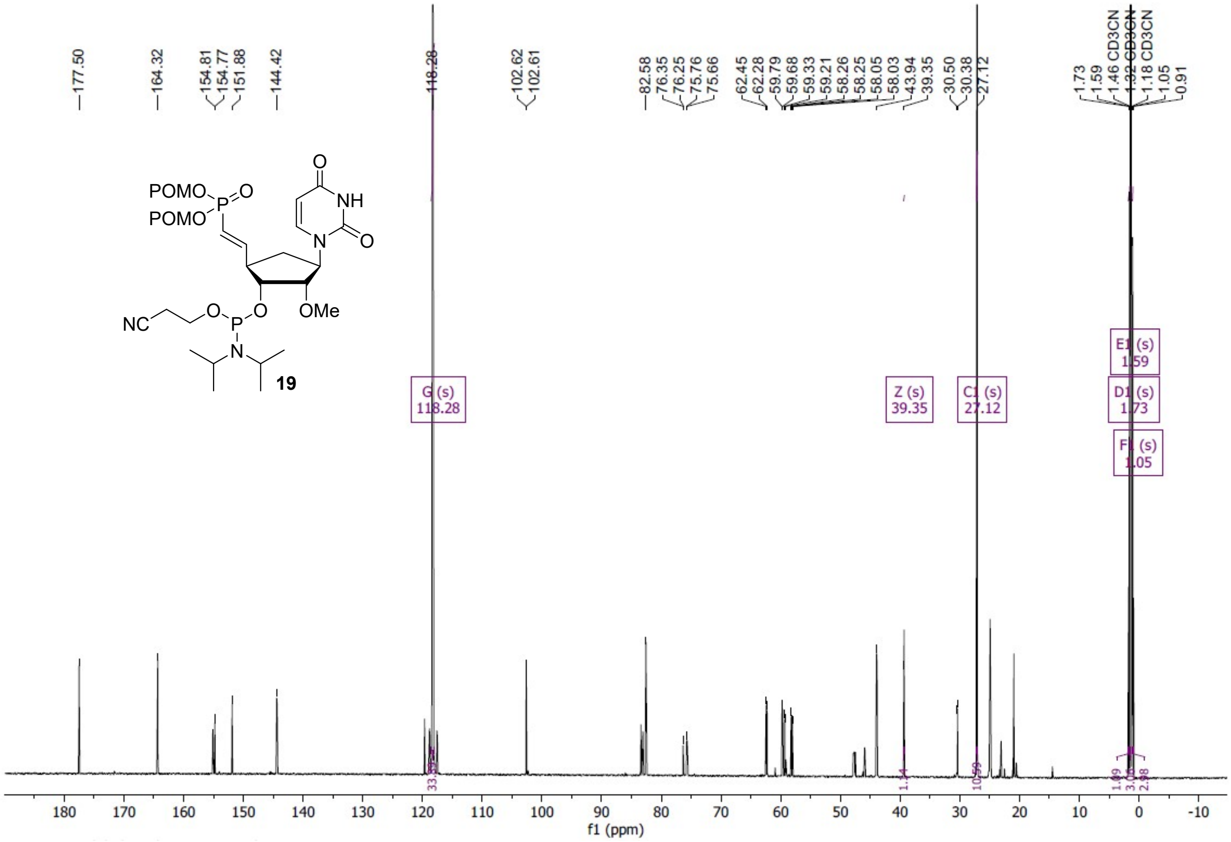

^{31}P NMR (243 MHz, CD_3CN) of compound **19**

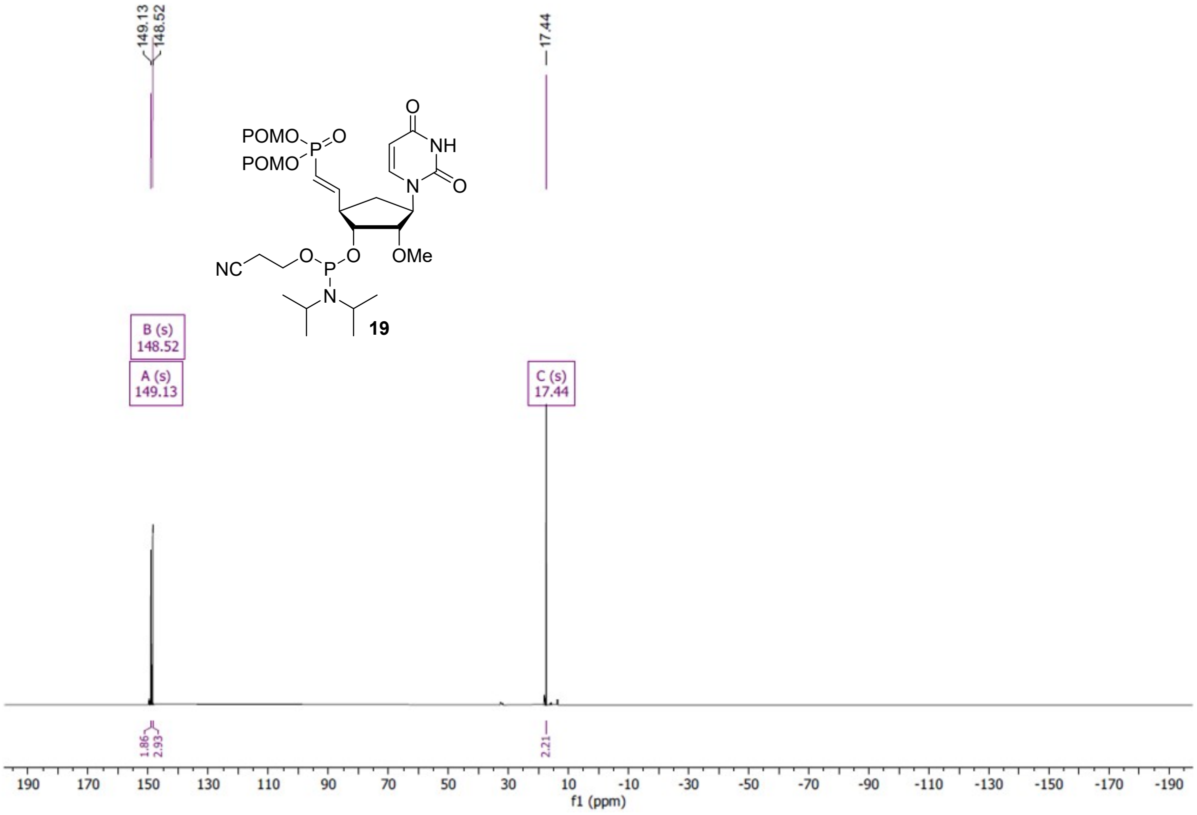

^{1}H NMR (600 MHz, CD_3CN) of compound **20**

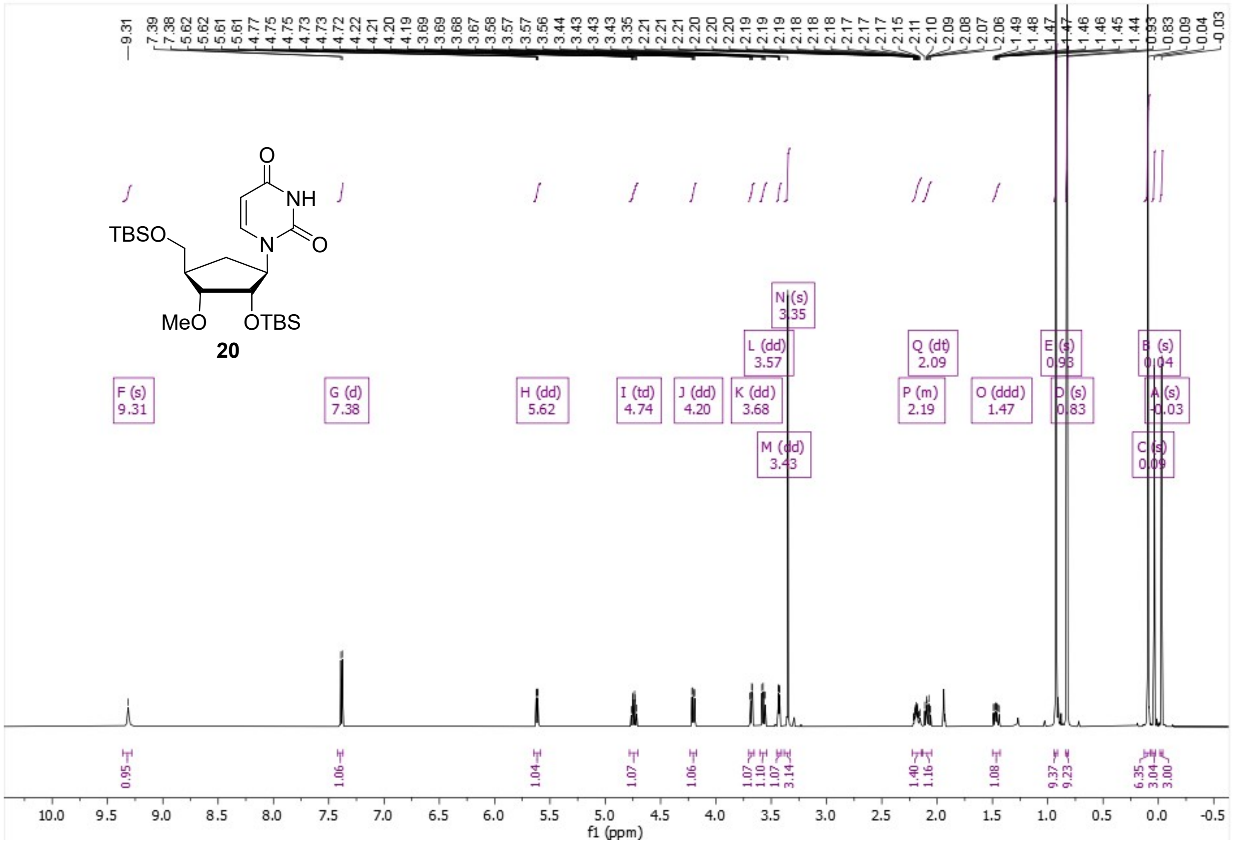

^{13}C NMR (151 MHz, CD_3CN) of compound **20**

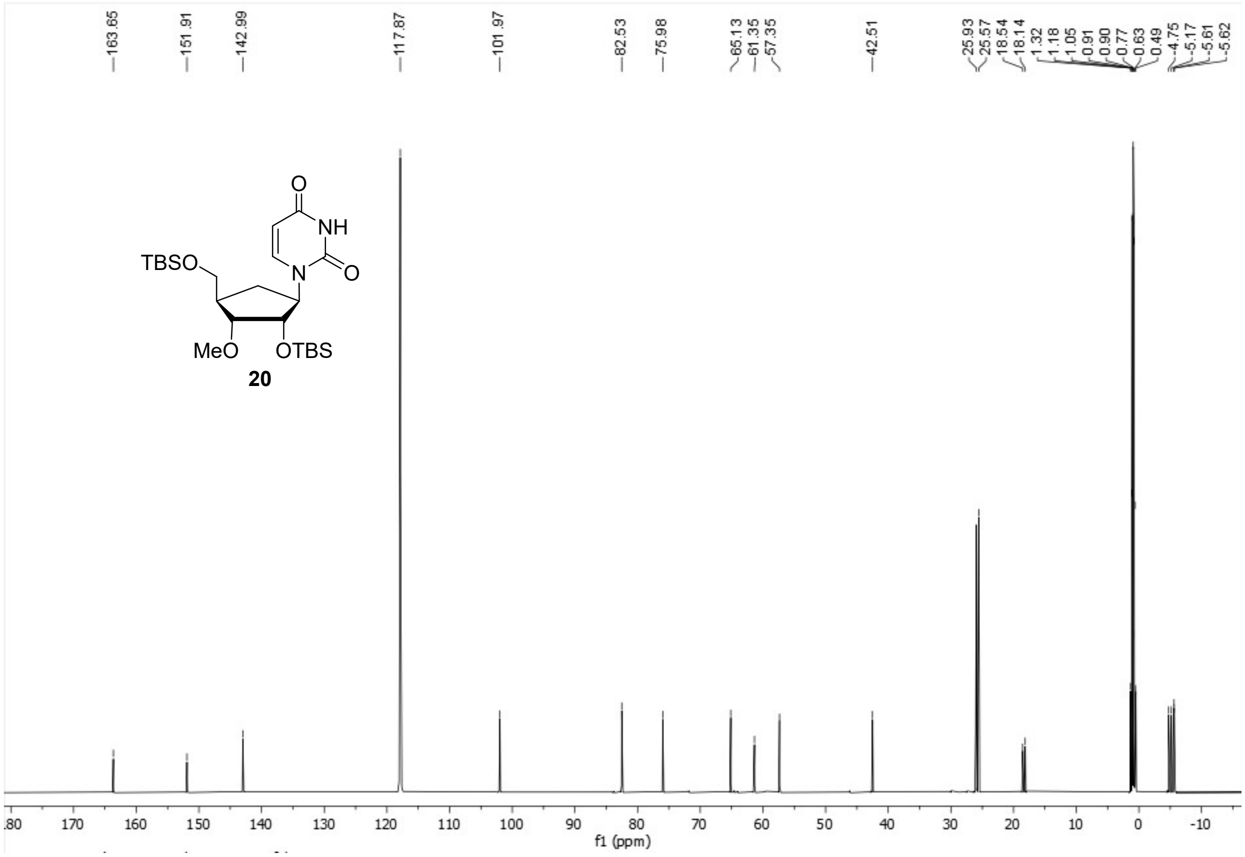

^{1}H NMR (600 MHz, DMSO-d_6) of compound **21**

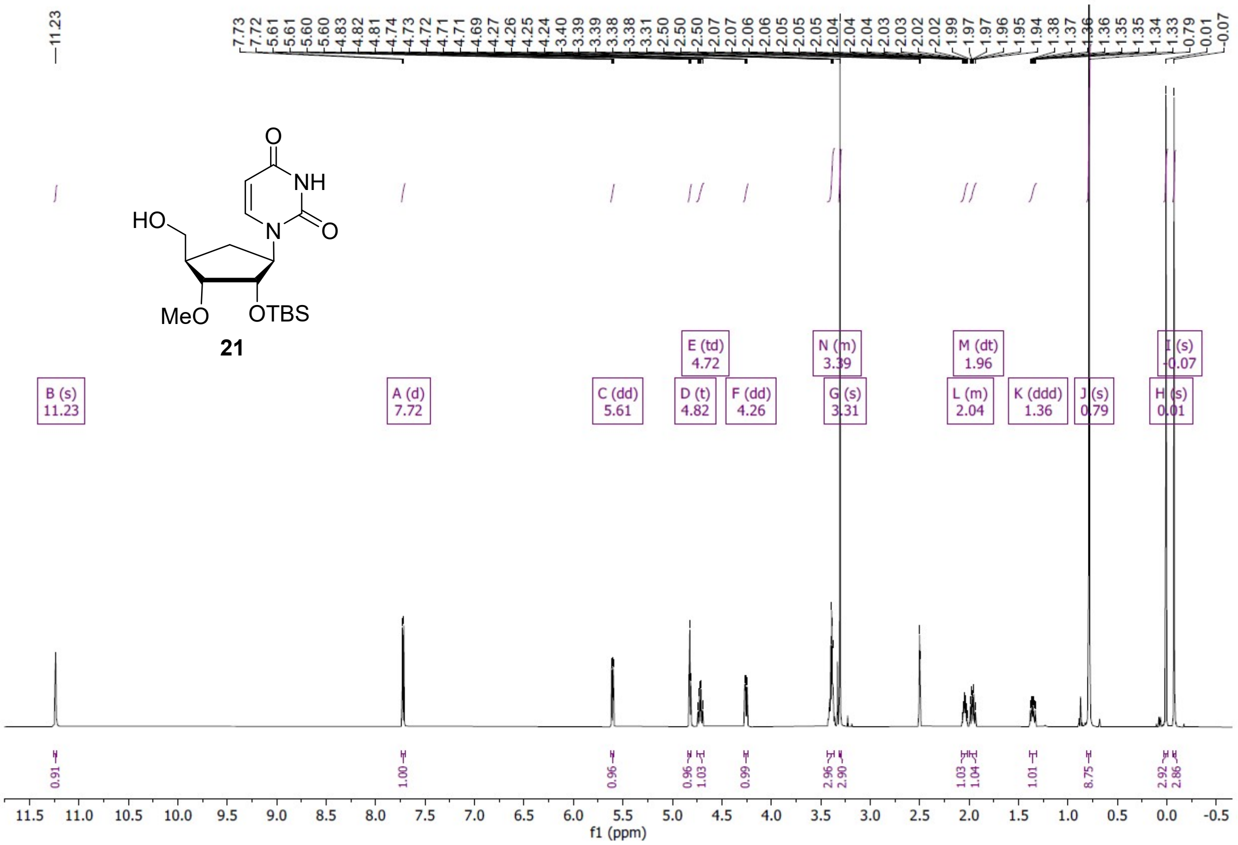

^{13}C NMR (151 MHz, DMSO-d_6) of compound **21**

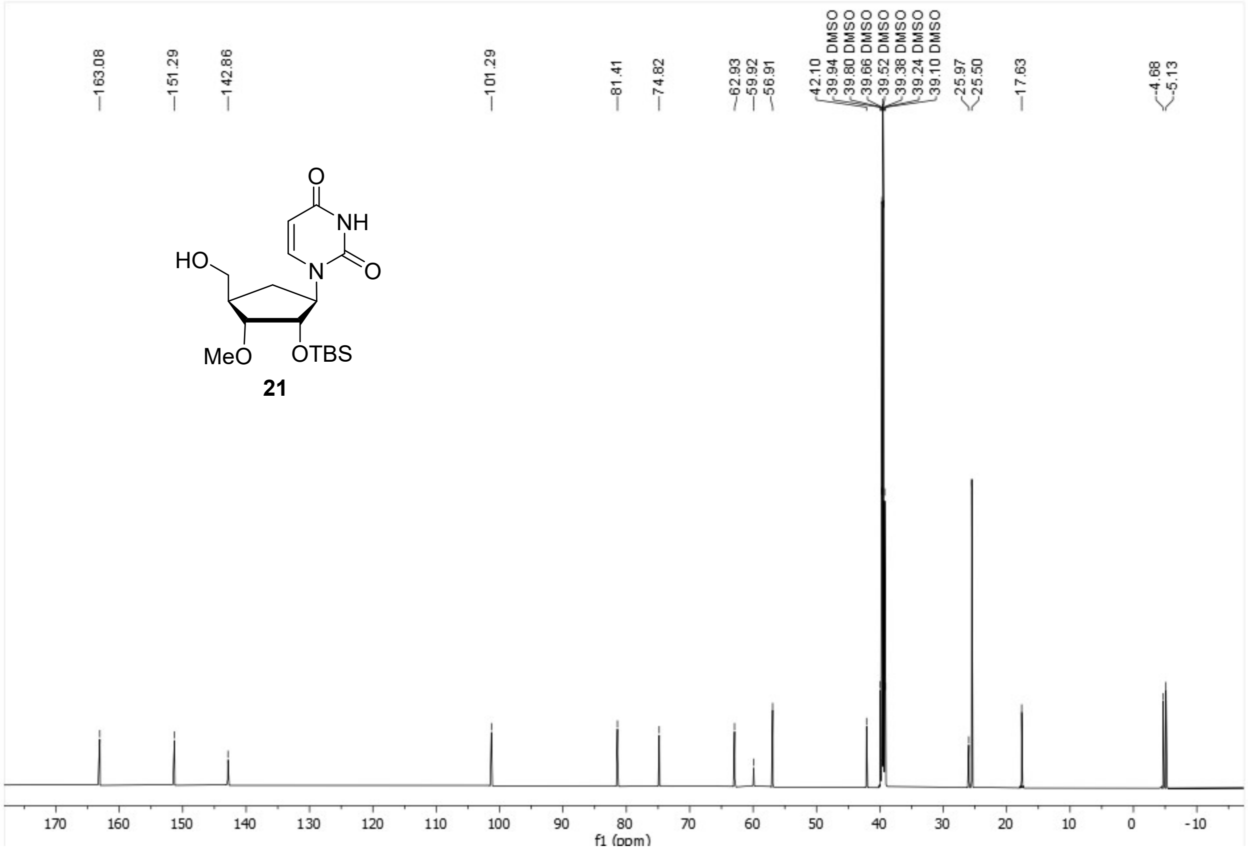

^{1}H NMR (600 MHz, CD_3CN) of compound **24**

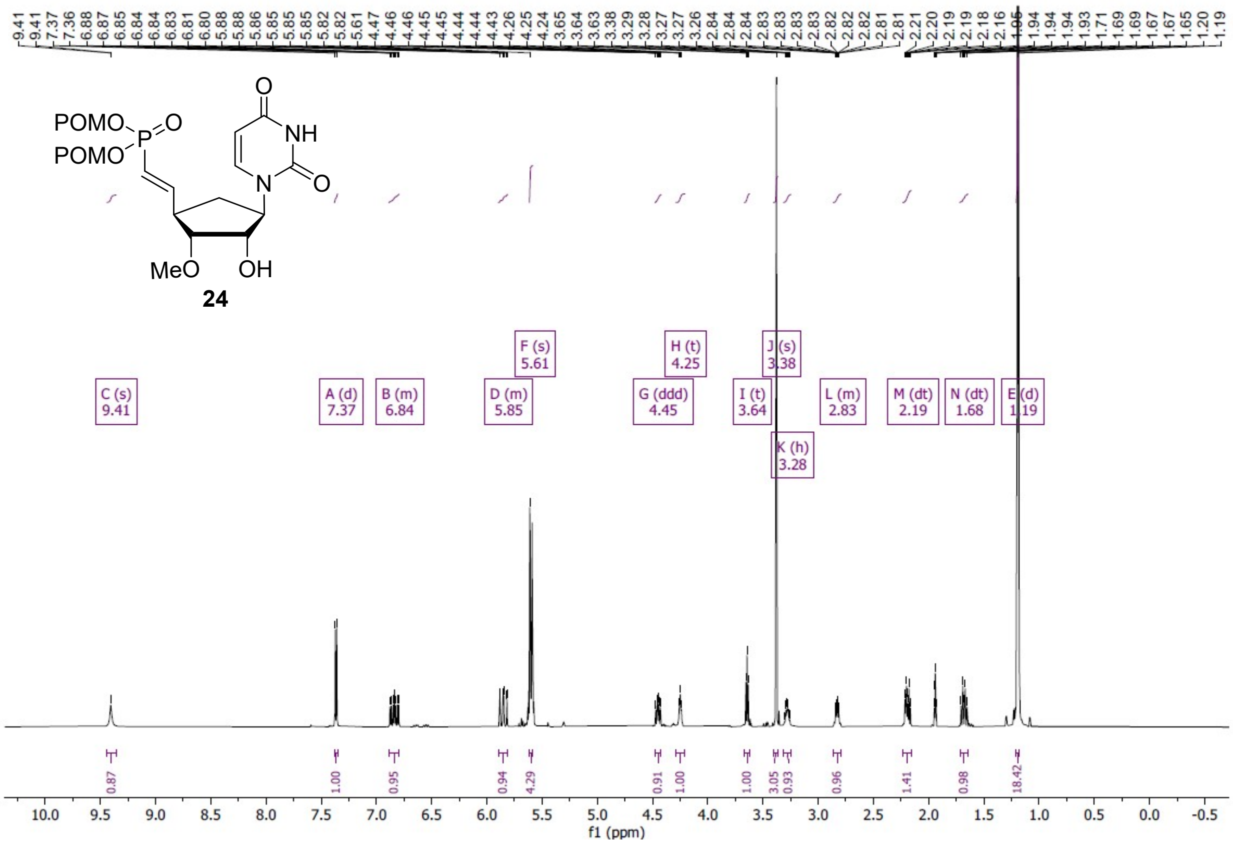

^{13}C NMR (151 MHz, CD_3CN) of compound **24**

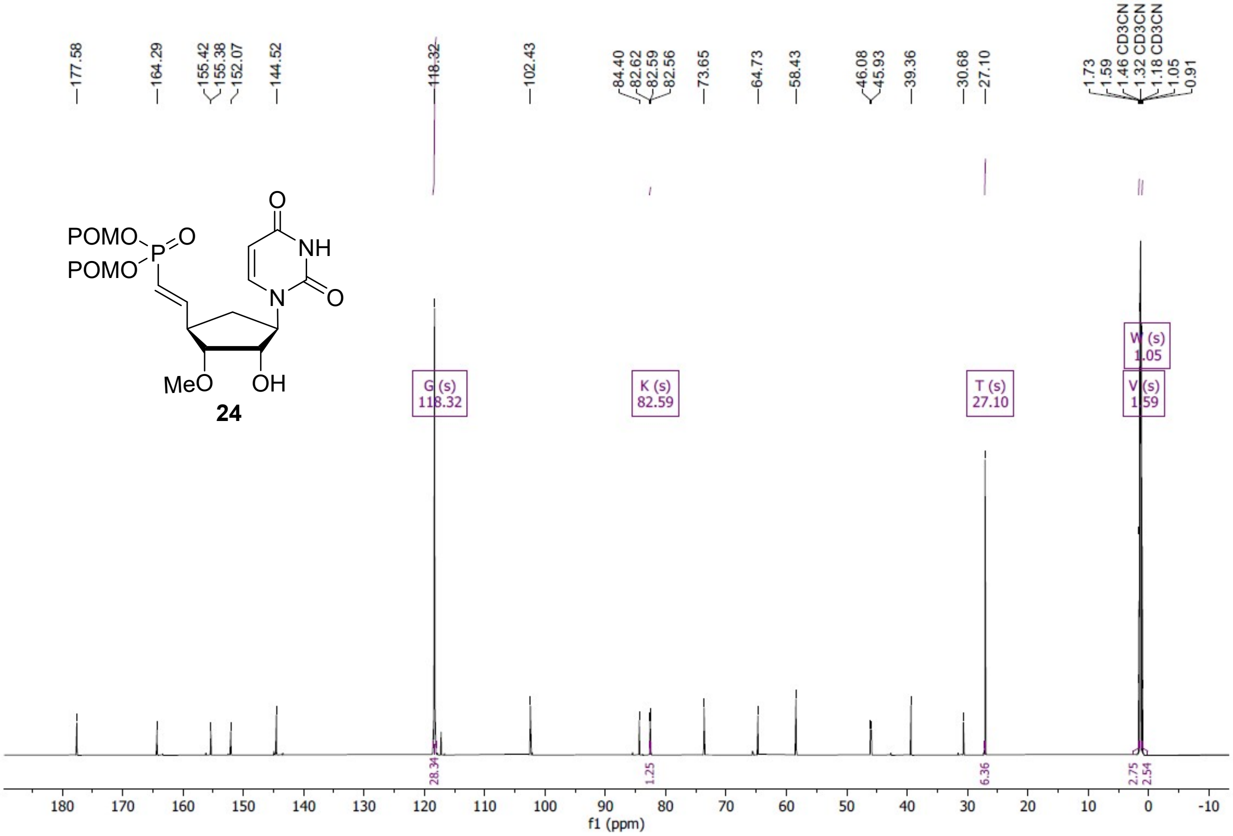

^{31}P NMR (243 MHz, CD_3CN) of compound **24**

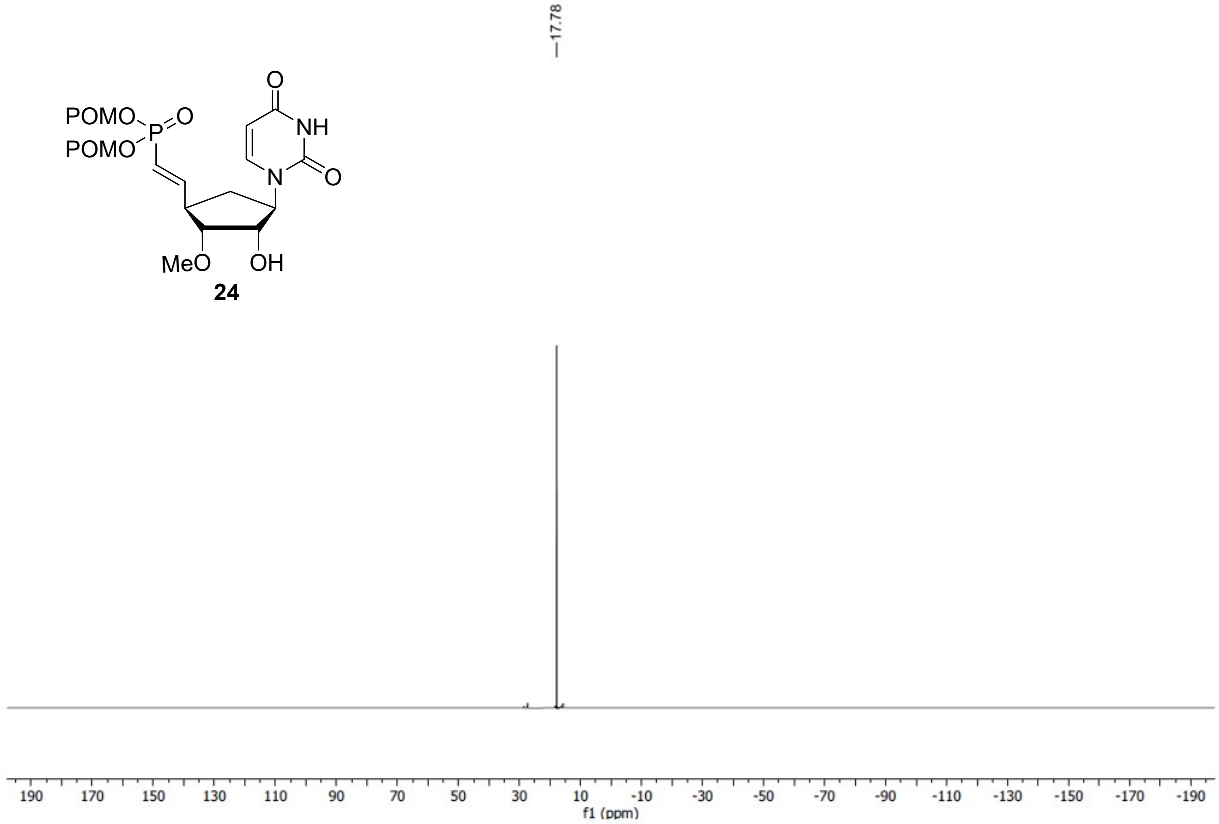

^{31}P NMR (243 MHz, CD_3CN) of compound (**E-Z mixture**)

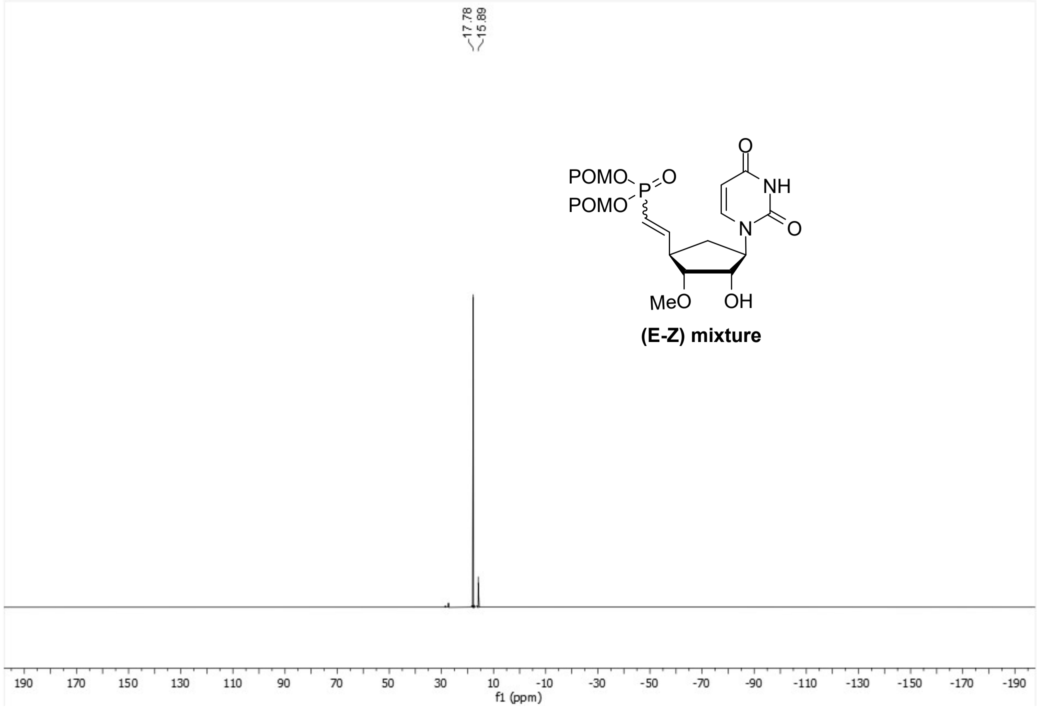

^1H NMR (600 MHz, CD_3CN) of compound **25**

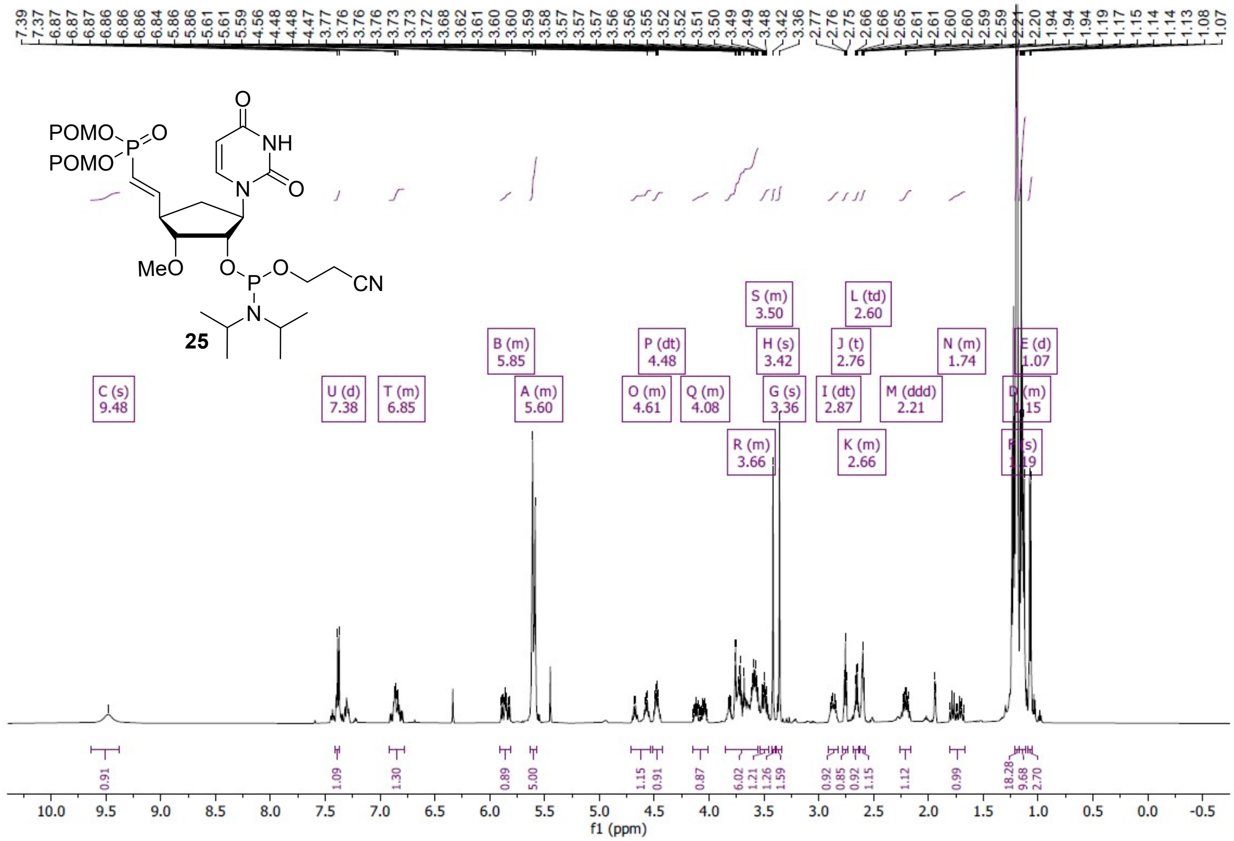

^{13}C NMR (151 MHz, CD_3CN) of compound **25**

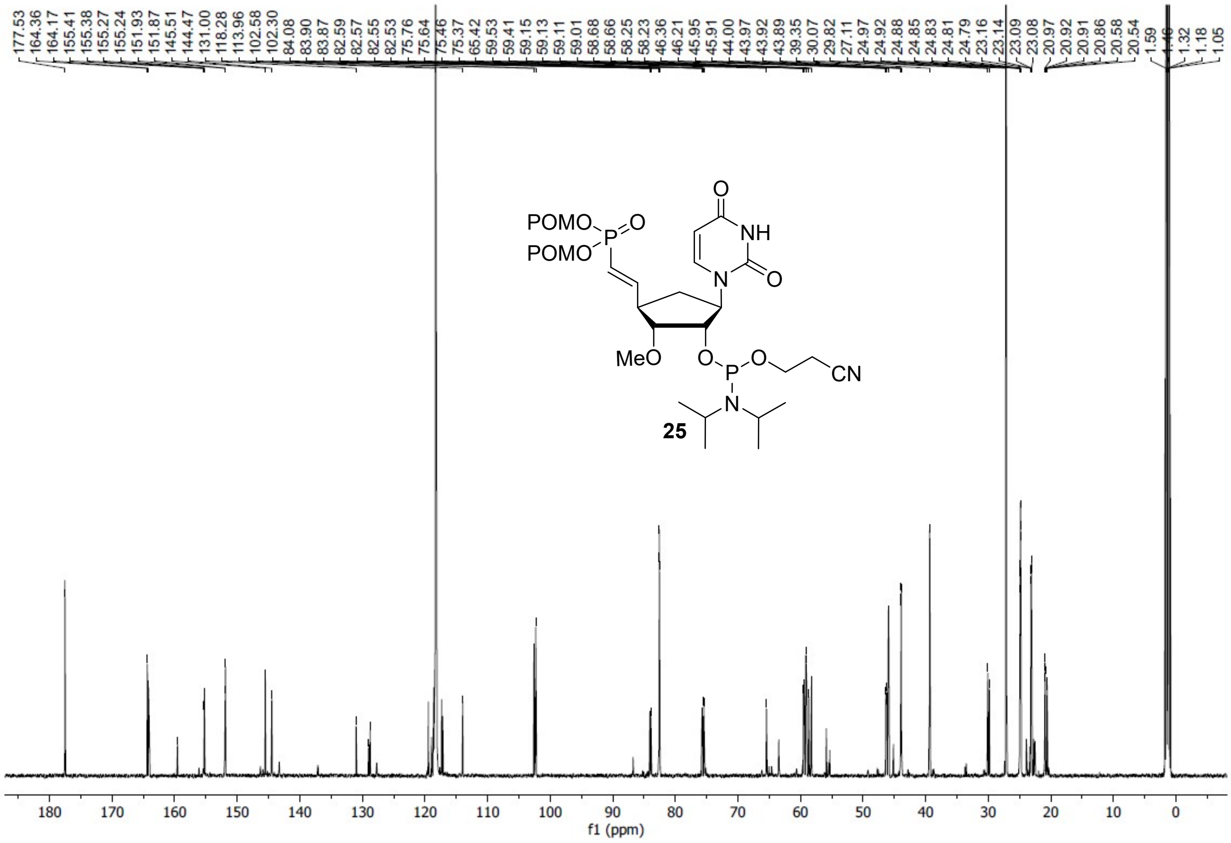

^{31}P NMR (243 MHz, CD_3CN) of compound **25**

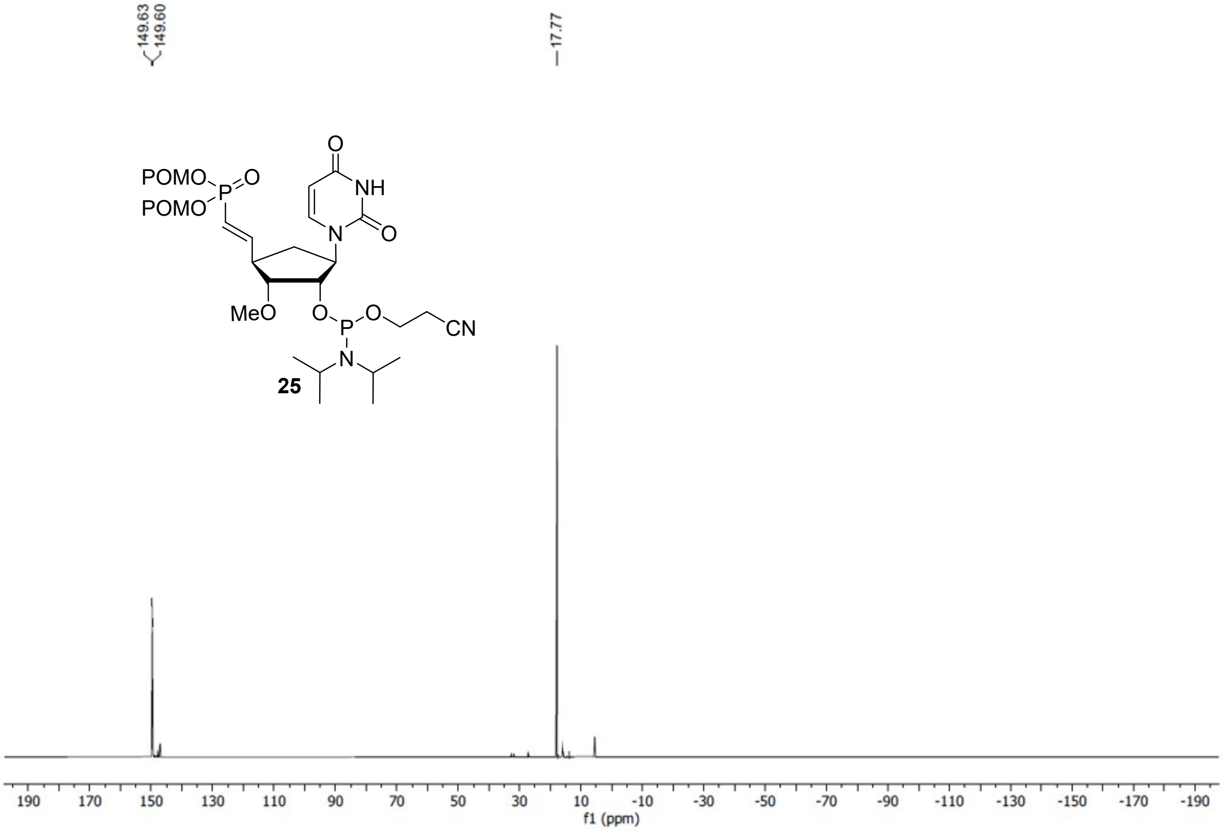

HPLC profiles of oligonucleotides

RP HPLC chromatogram for **ON1**

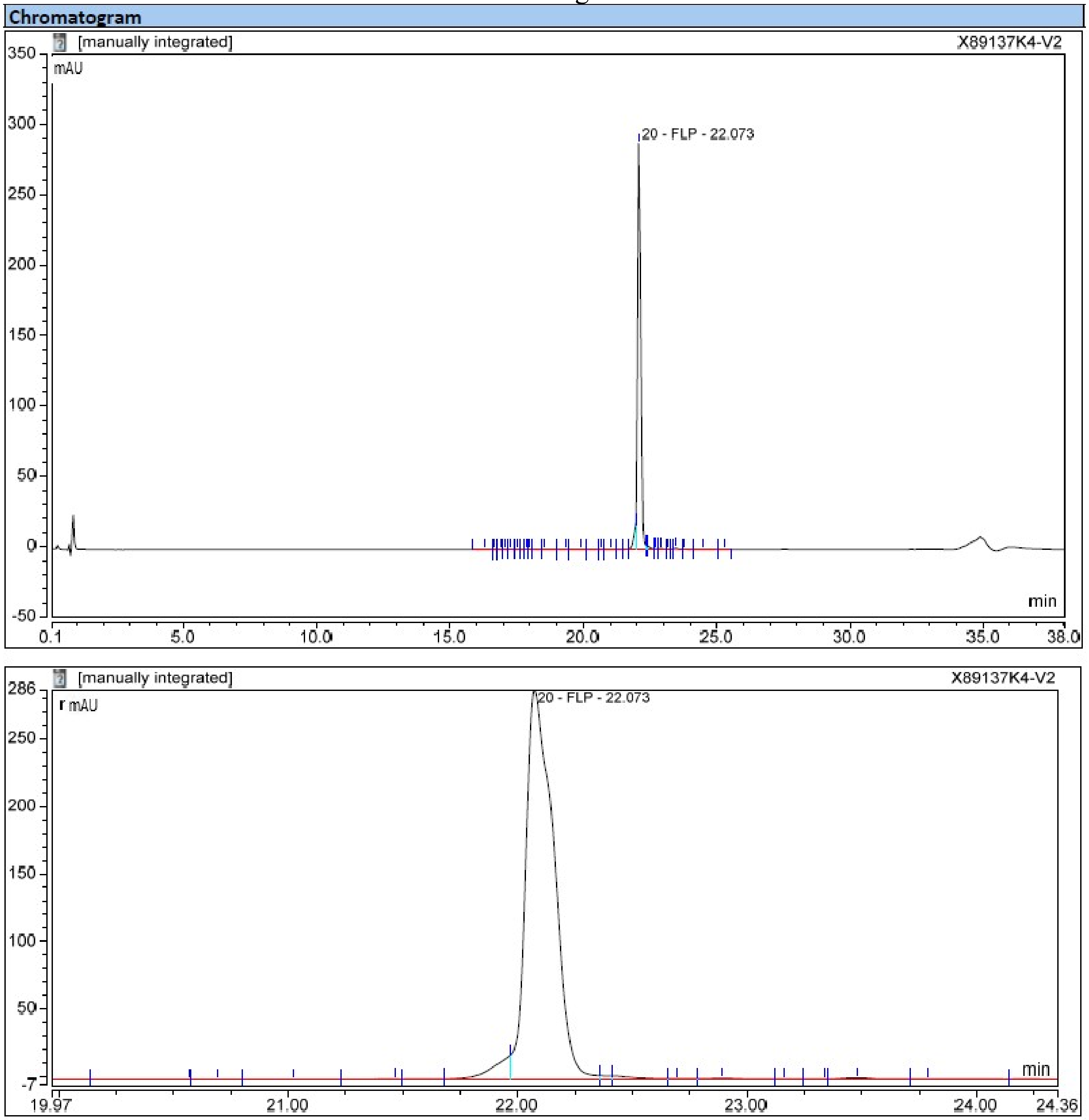

RP HPLC chromatogram for **ON2**

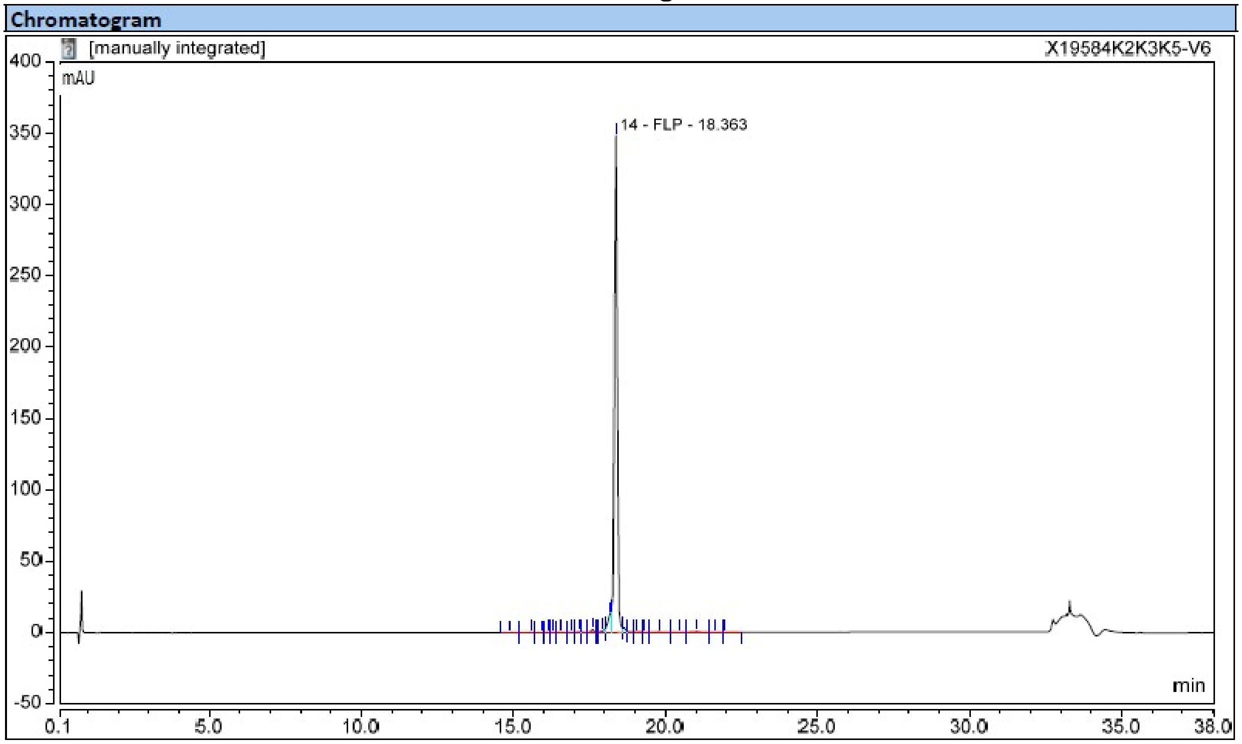

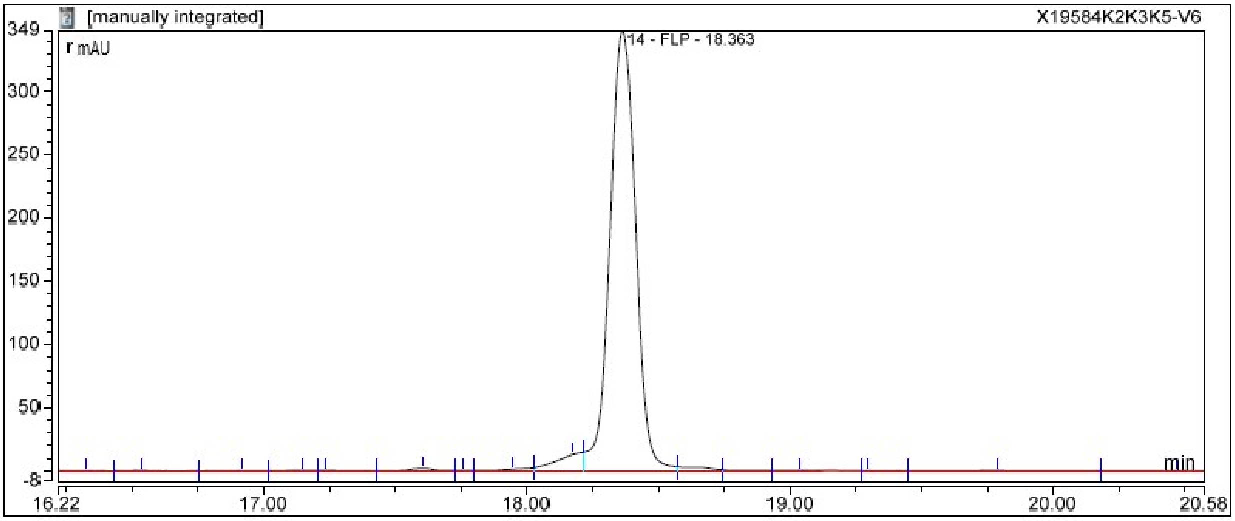

RP HPLC chromatogram for **ON3**

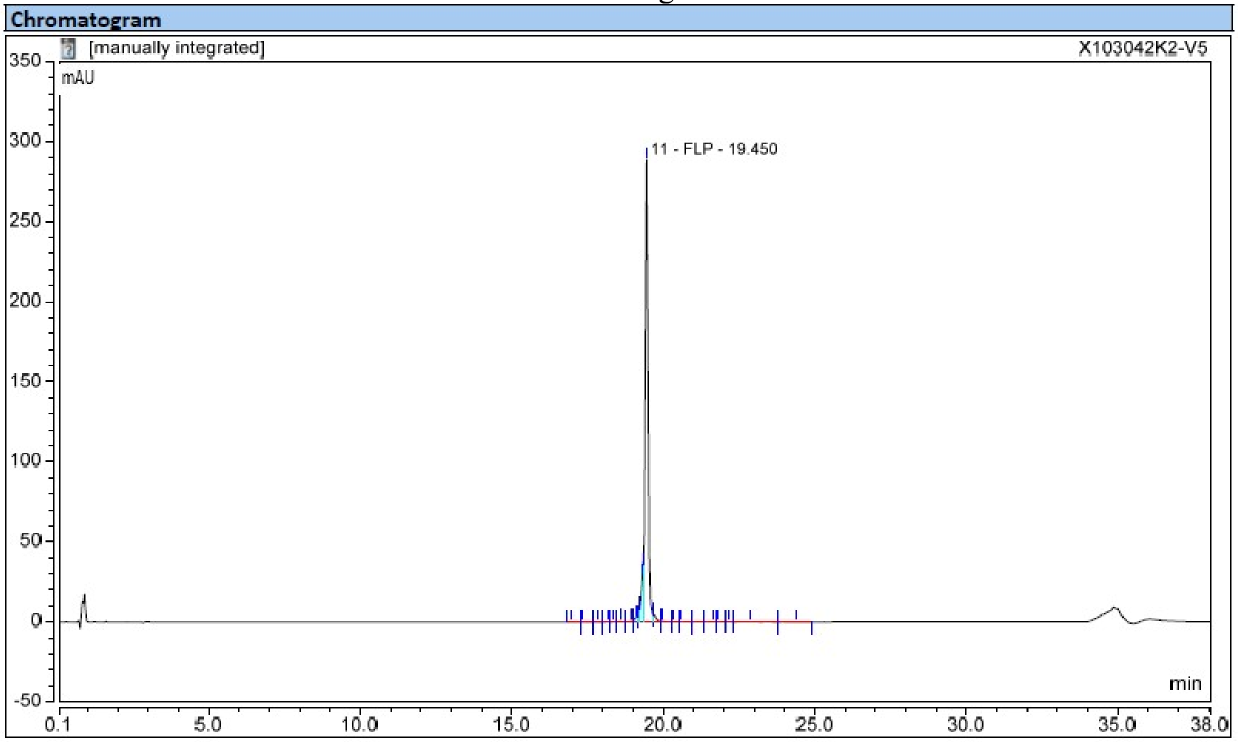

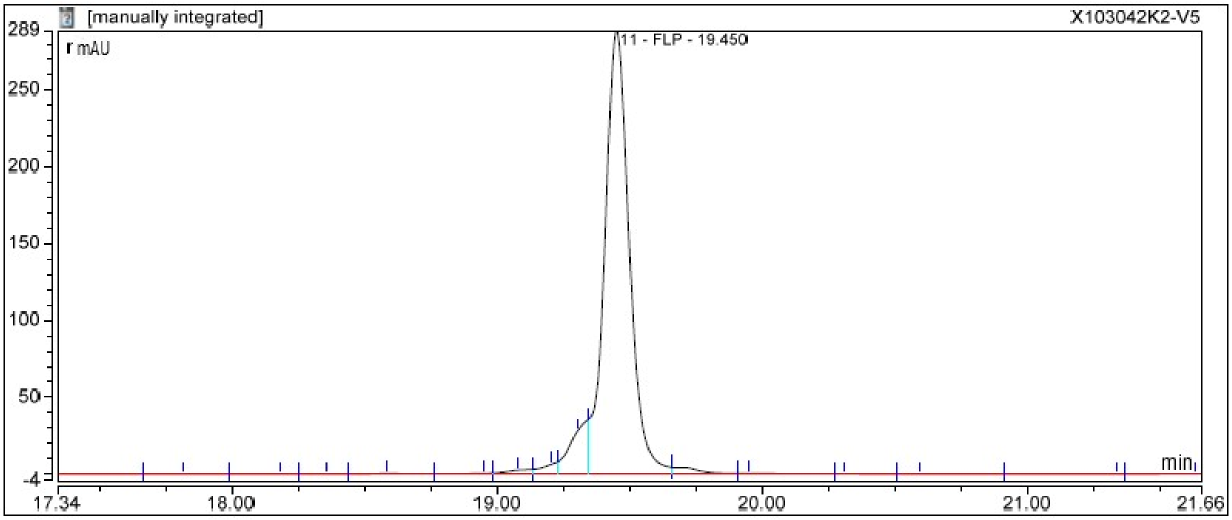

RP HPLC chromatogram for **ON4**

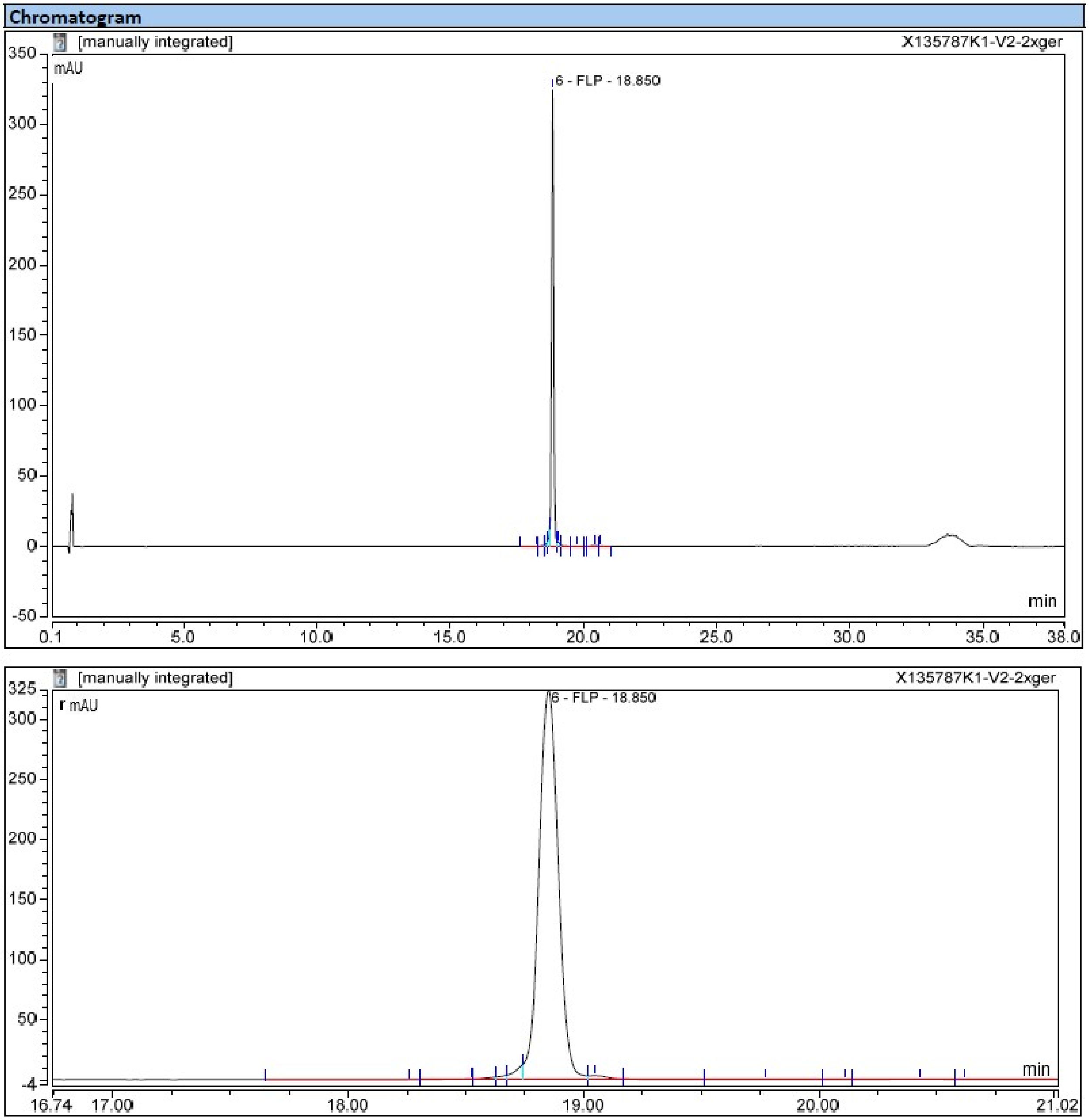

IEX HPLC chromatogram for **ON5**

Chromatogram

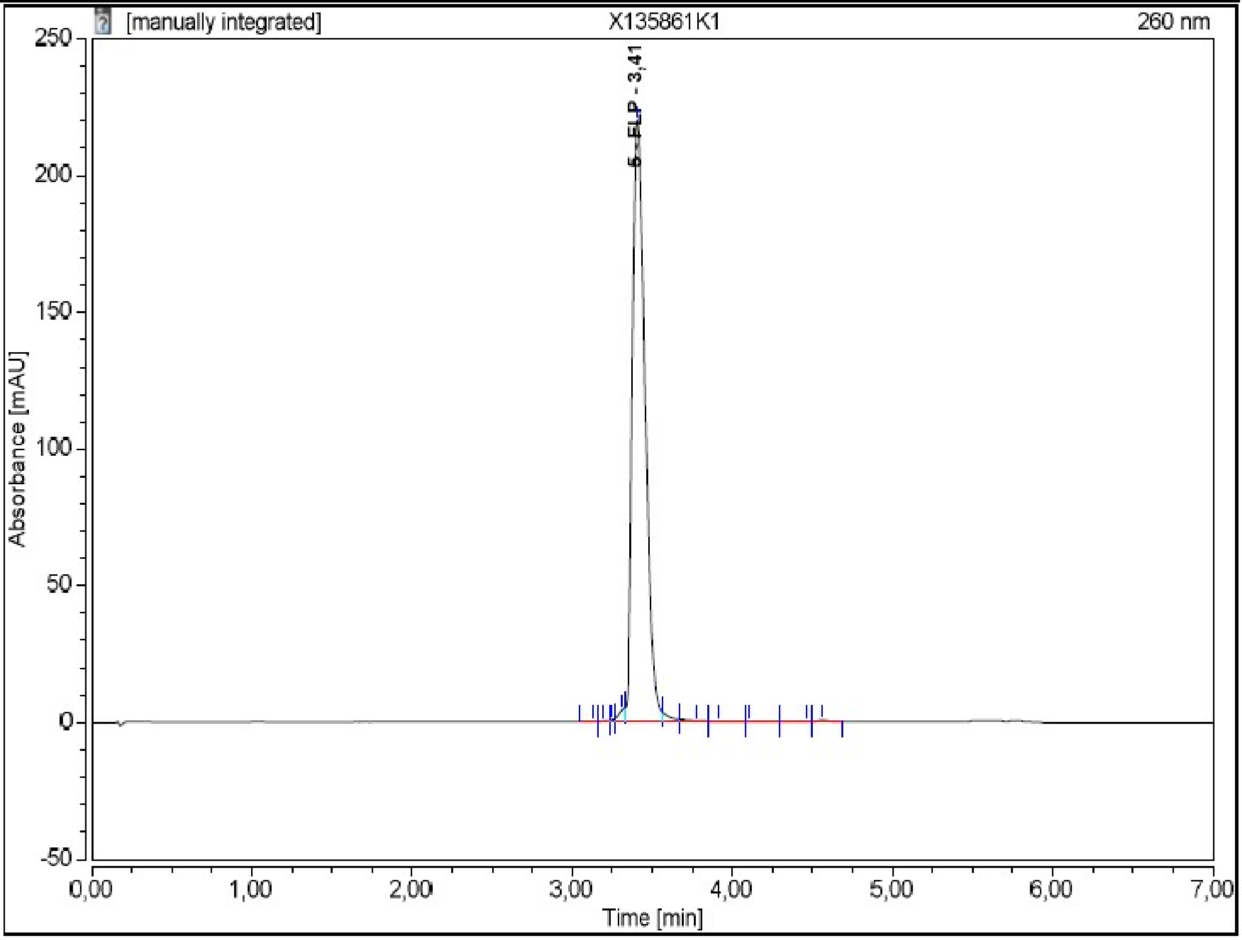

Integration Results

No.	Peak Name	Ret.Time min	Area mAU*min	Height mAU	Realitve Area %	Peak Width min	Res (USP) to FLP
1		3,13	0,005	0,068	0,03	n.a.	n.a.
2		3,20	0,016	0,199	0,08	0,09	1,79
3		3,24	0,017	0,469	0,08	n.a.	n.a.
4		3,31	0,208	4,165	1,00	n.a.	n.a.
5	FLP	3,41	20,108	219,779	97,29	0,15	0,00
6		3,56	0,184	3,498	0,89	n.a.	n.a.
7		3,78	0,068	0,293	0,33	0,07	3,36
8		3,92	0,019	0,130	0,09	0,25	2,56
9		4,11	0,003	0,030	0,01	n.a.	n.a.
10		4,47	0,006	0,063	0,03	0,12	7,80
11		4,56	0,035	0,540	0,17	0,10	9,38
Total:			20,67	229,235	100,00	0,78	

RP HPLC chromatogram for **ON6**

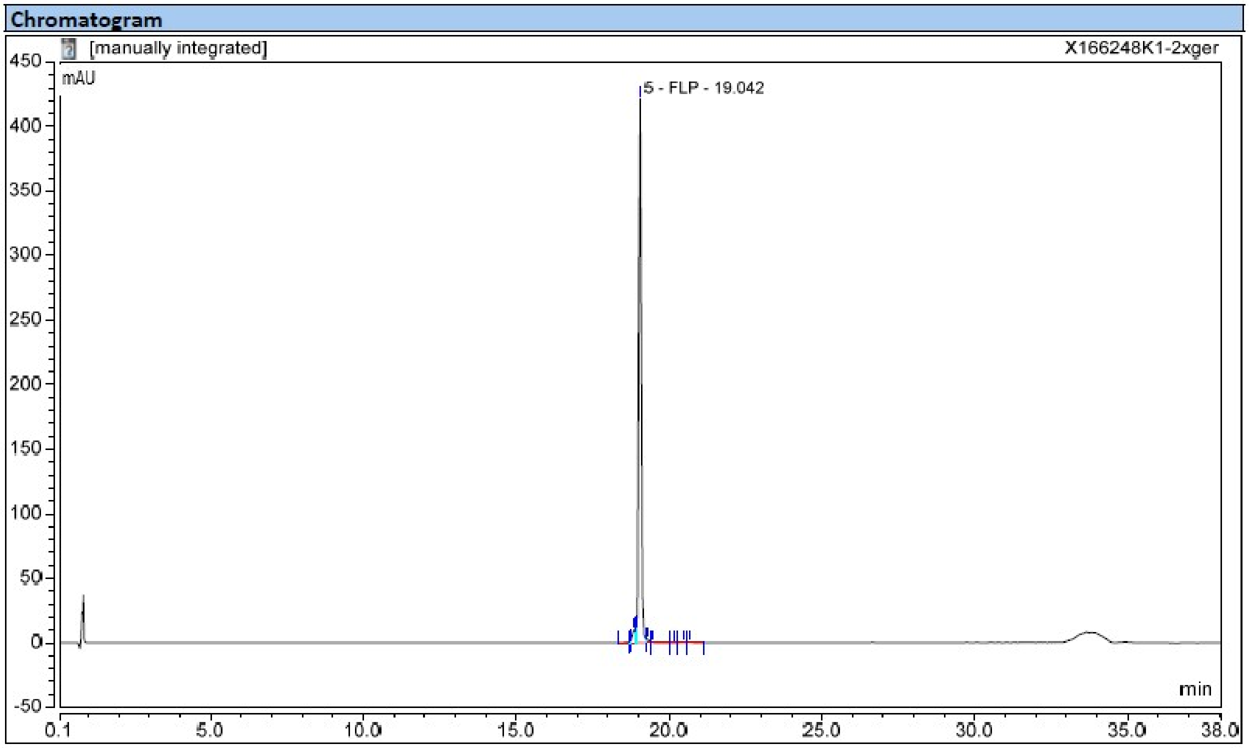

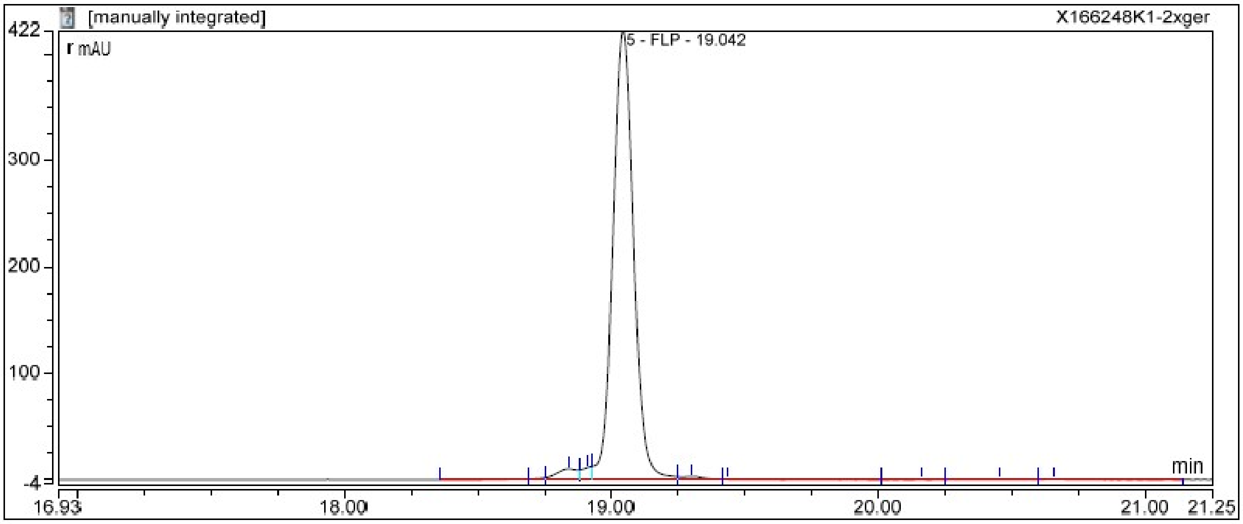

RP HPLC chromatogram for **ON7**

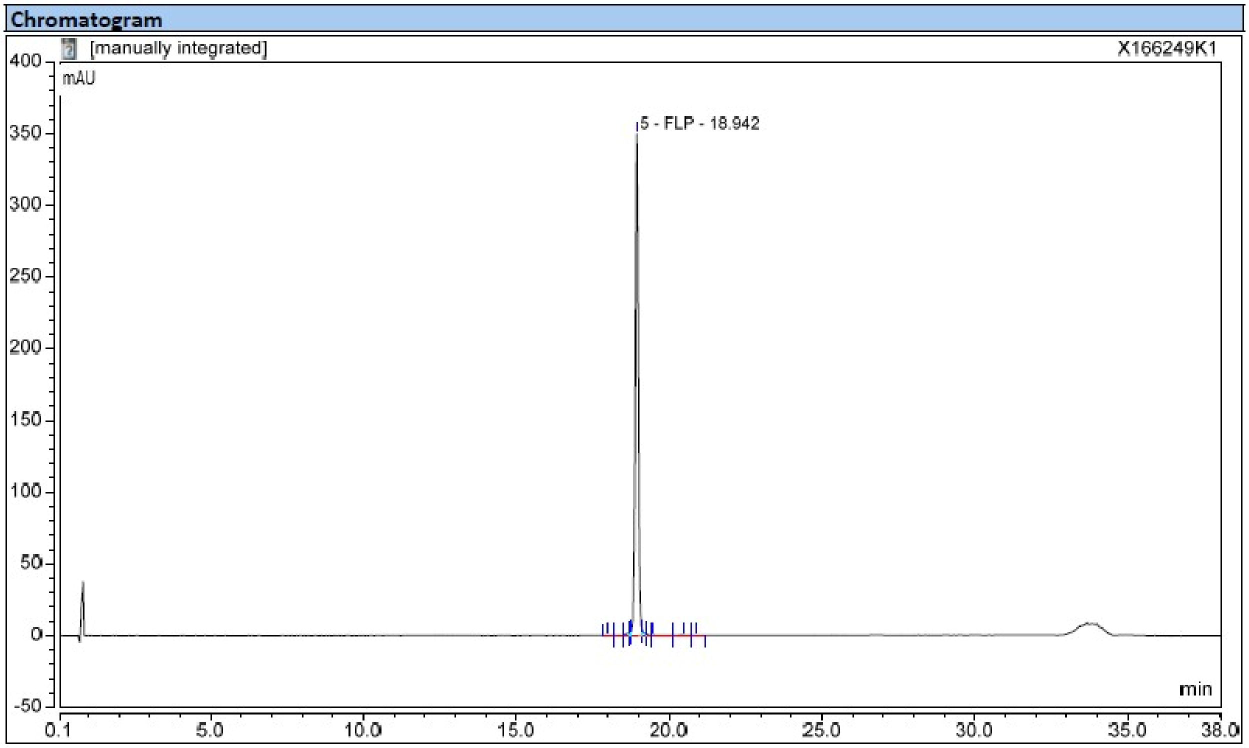

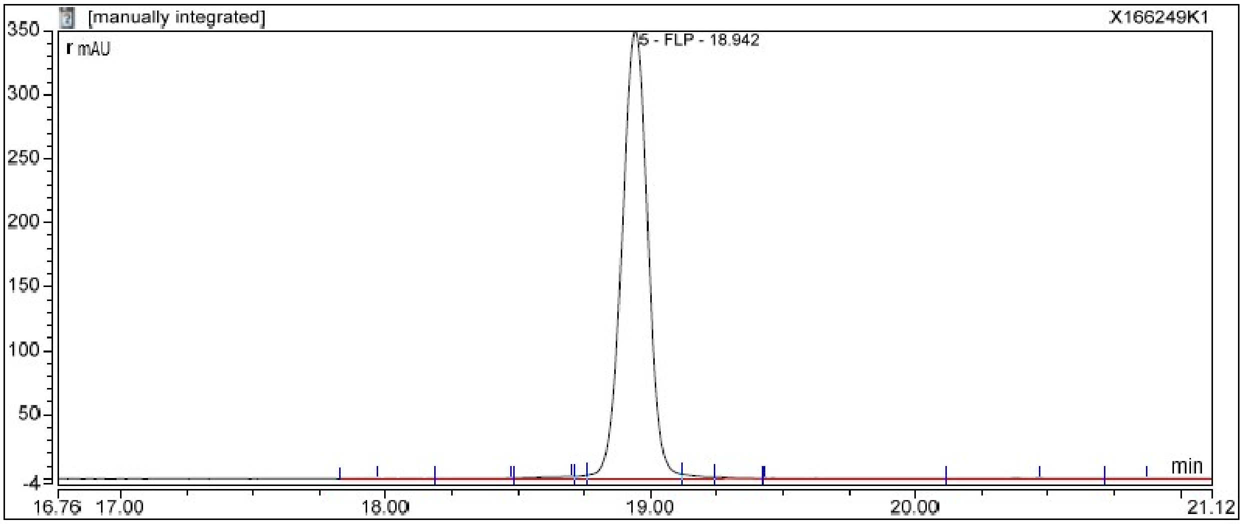

IEX HPLC chromatogram for **ON8**

Chromatogram

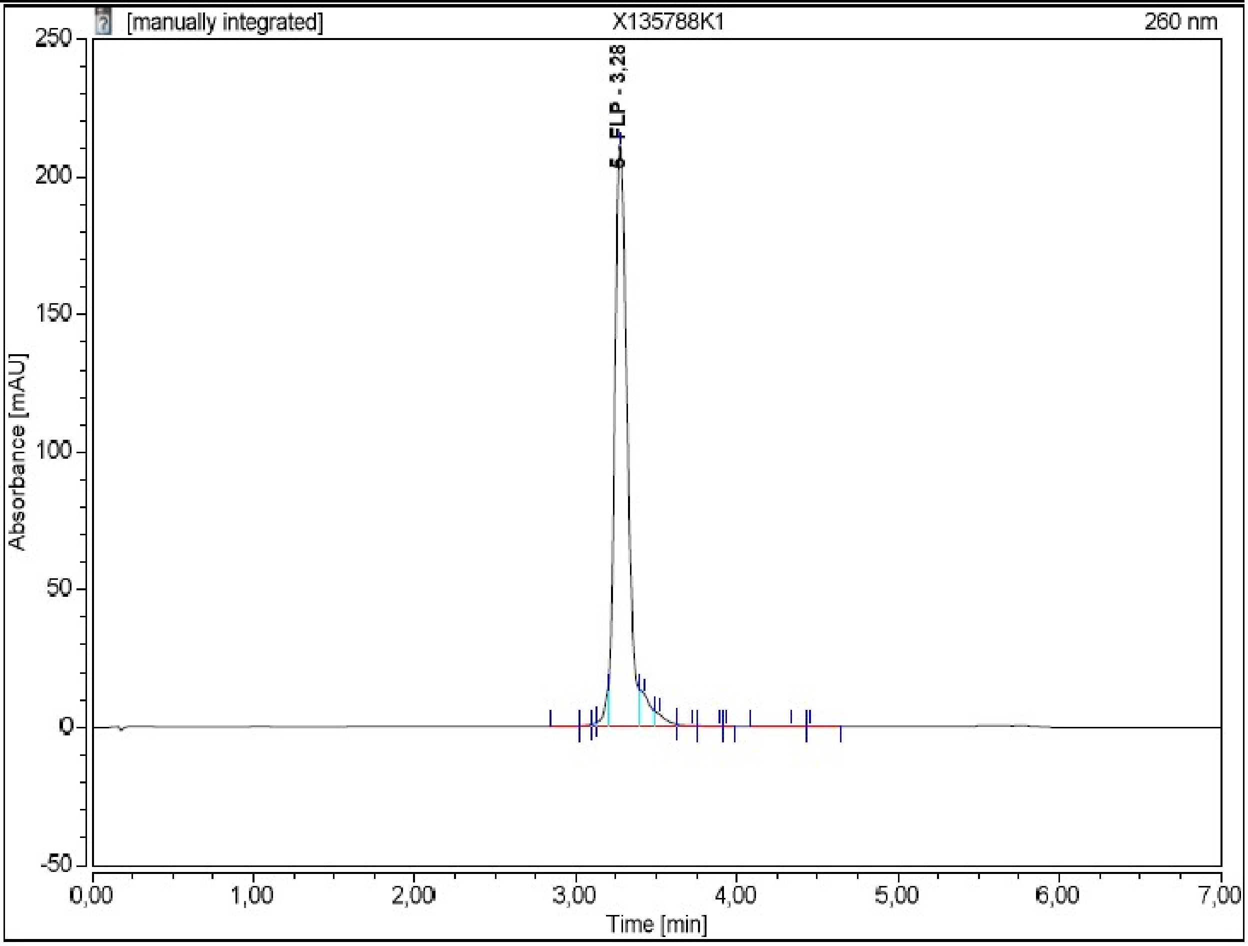

Integration Results

No.	Peak Name	Ret.Time min	Area mAU*min	Height mAU	Realitve Area %	Peak Width min	Res (USP) to FLP
1		3,02	0,008	0,124	0,04	n.a.	n.a.
2		3,09	0,021	0,540	0,10	n.a.	n.a.
3		3,13	0,037	1,394	0,18	n.a.	n.a.
4		3,20	0,333	13,125	1,62	n.a.	n.a.
5	FLP	3,28	18,827	210,227	91,73	0,14	0,00
6		3,43	0,831	11,733	4,05	n.a.	n.a.
7		3,52	0,392	4,382	1,91	n.a.	n.a.
8		3,72	0,041	0,212	0,20	n.a.	n.a.
9		3,89	0,011	0,034	0,05	n.a.	n.a.
10		3,93	0,001	0,027	0,00	n.a.	n.a.
11		4,34	0,019	0,104	0,09	n.a.	n.a.
12		4,45	0,003	0,034	0,02	n.a.	n.a.
Total:			20,52	241,936	100,00	0,14	

IEX HPLC chromatogram for **ON9**

Chromatogram

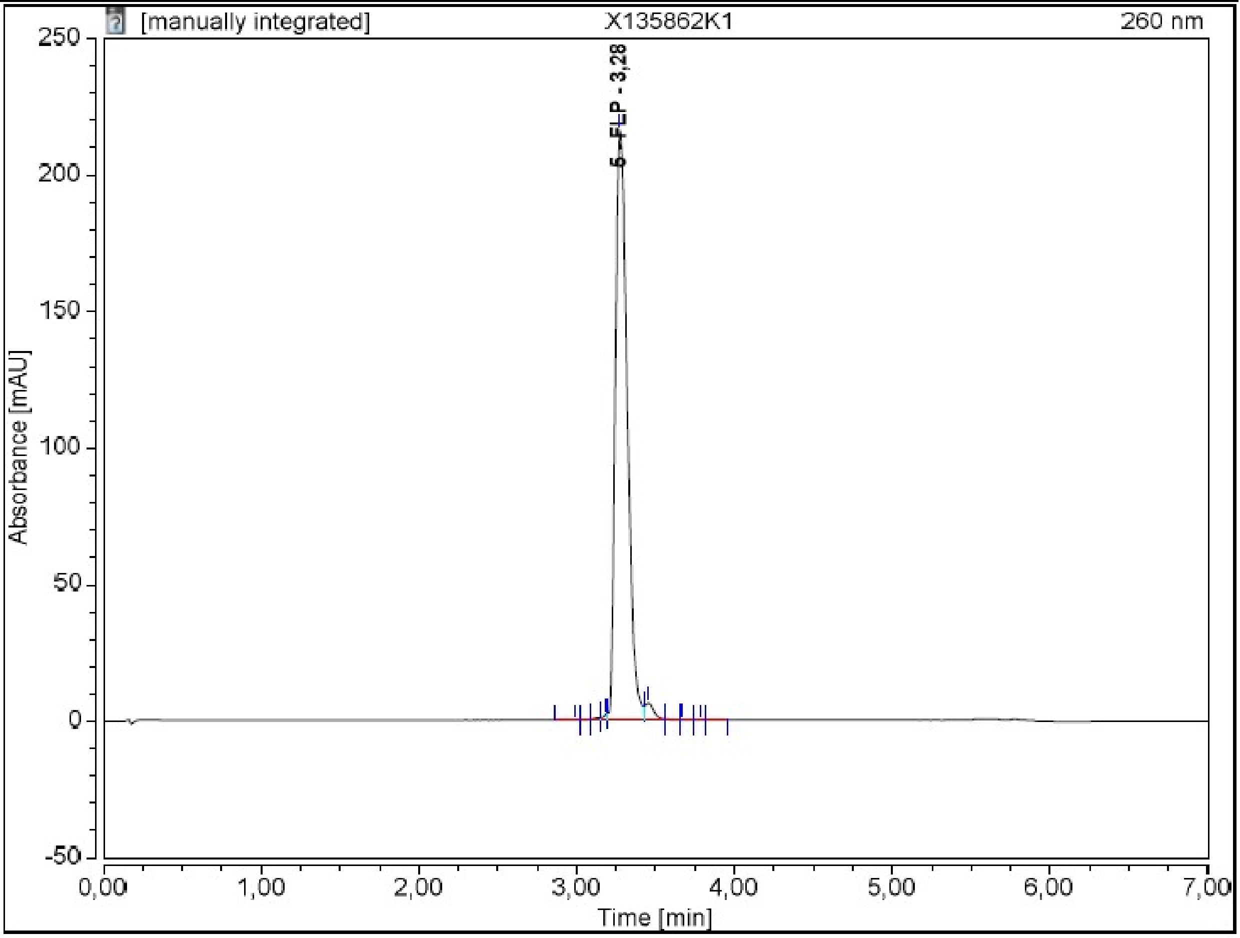

Integration Results

No.	Peak Name	Ret.Time min	Area mAU*min	Height mAU	Realitve Area %	Peak Width min	Res (USP) to FLP
1		2,99	0,005	0,061	0,03	0,11	2,31
2		3,09	0,008	0,247	0,04	n.a.	n.a.
3		3,16	0,046	0,898	0,23	n.a.	n.a.
4		3,19	0,061	2,074	0,31	n.a.	n.a.
5	FLP	3,28	19,101	216,046	97,08	0,14	0,00
6		3,46	0,426	6,252	2,17	0,17	1,16
7		3,56	0,015	0,319	0,07	n.a.	n.a.
8		3,66	0,006	0,094	0,03	n.a.	n.a.
9		3,78	0,003	0,056	0,02	n.a.	n.a.
10		3,82	0,004	0,053	0,02	n.a.	n.a.
**Total:**			19,68	226,100	100,00	0,42	

RP HPLC chromatogram for **ON10**

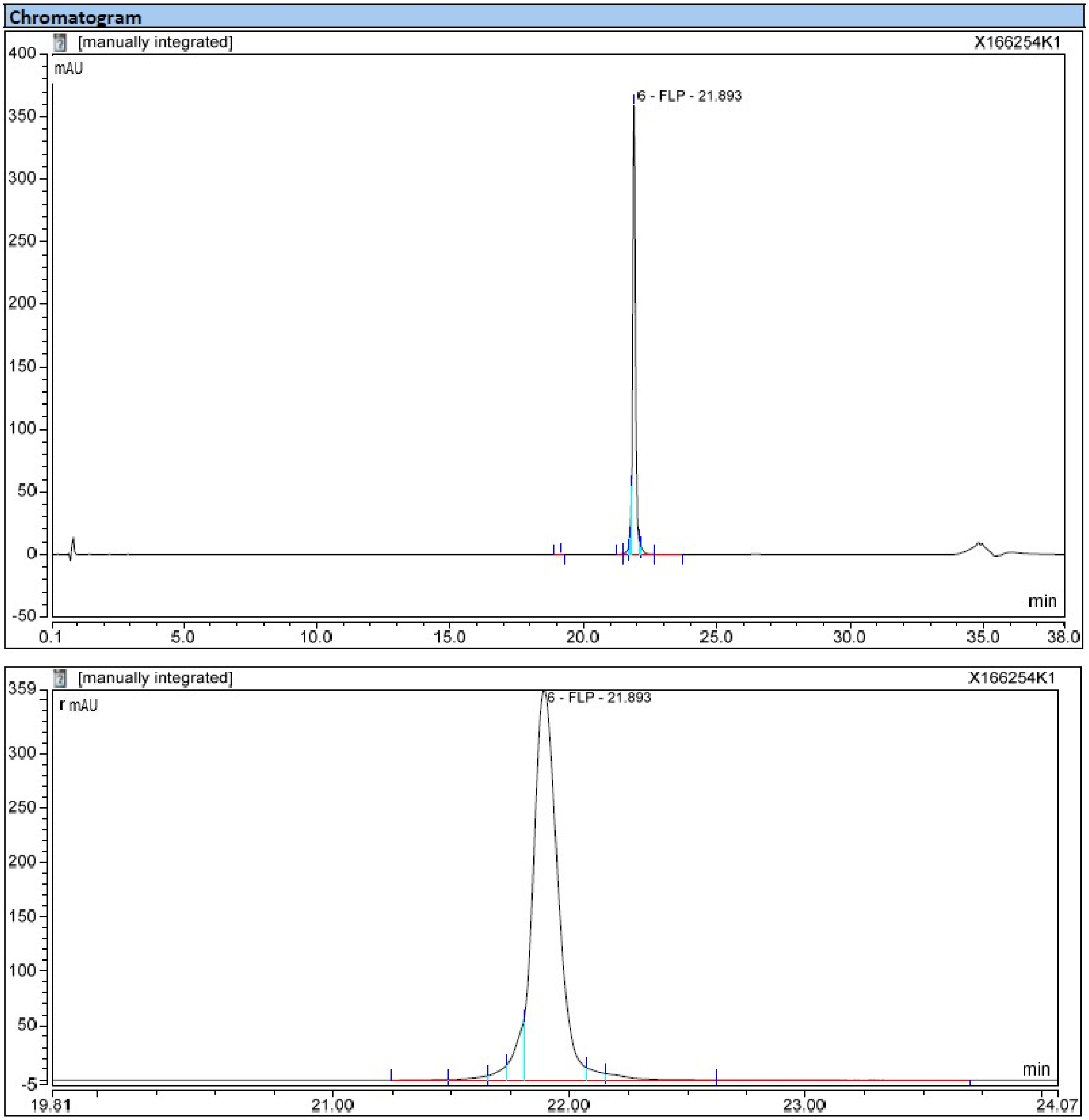

RP HPLC chromatogram for **ON11**

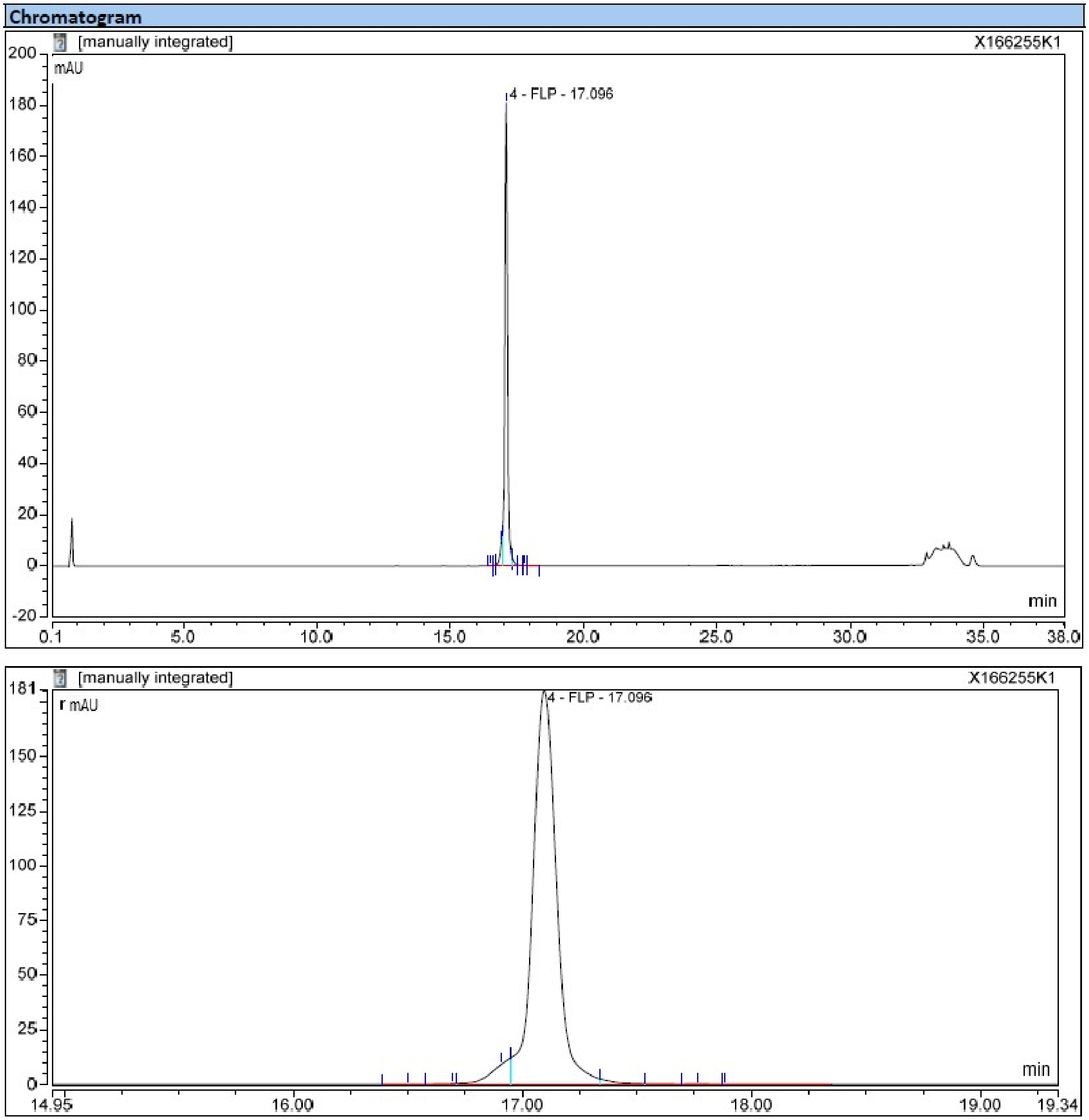

RP HPLC chromatogram for **ON12**

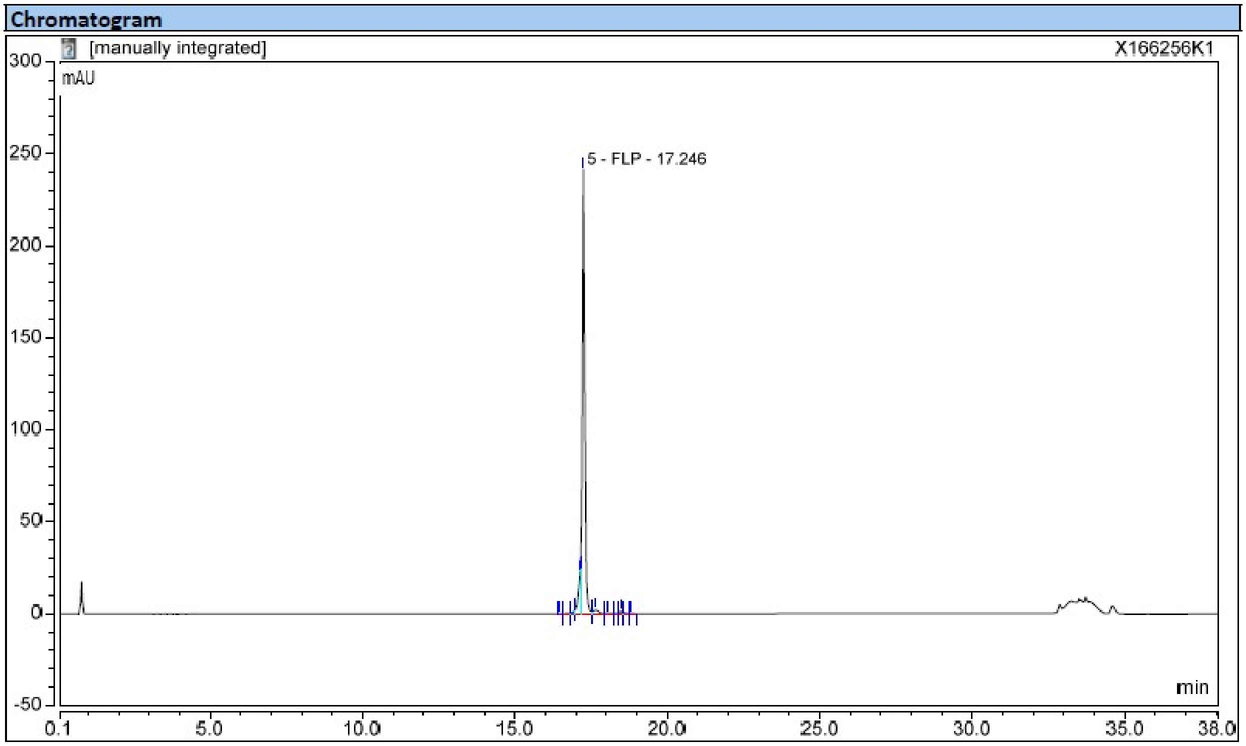

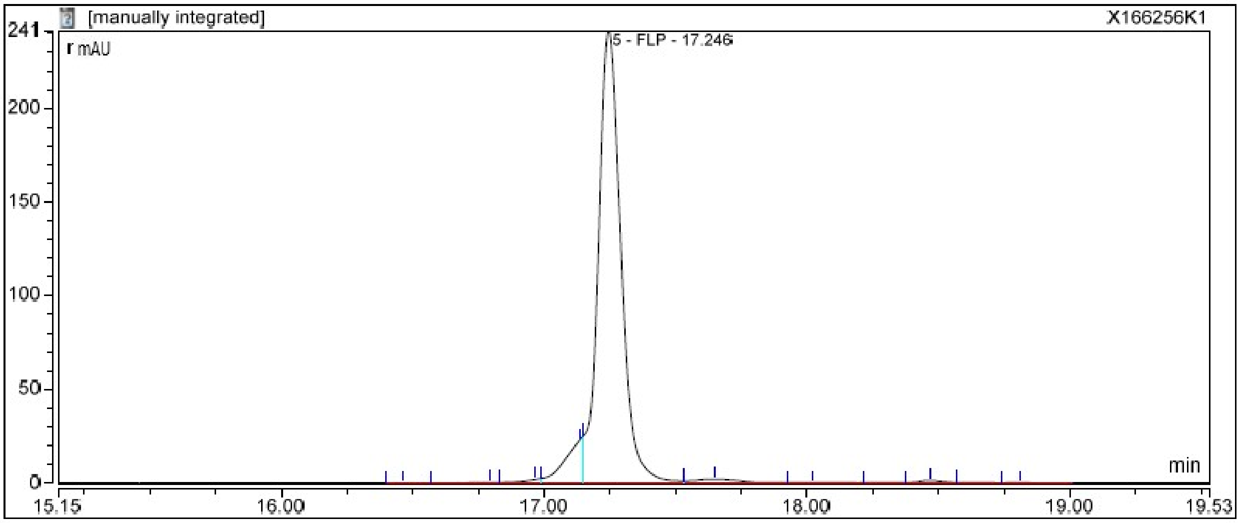

RP HPLC chromatogram for **ON13**

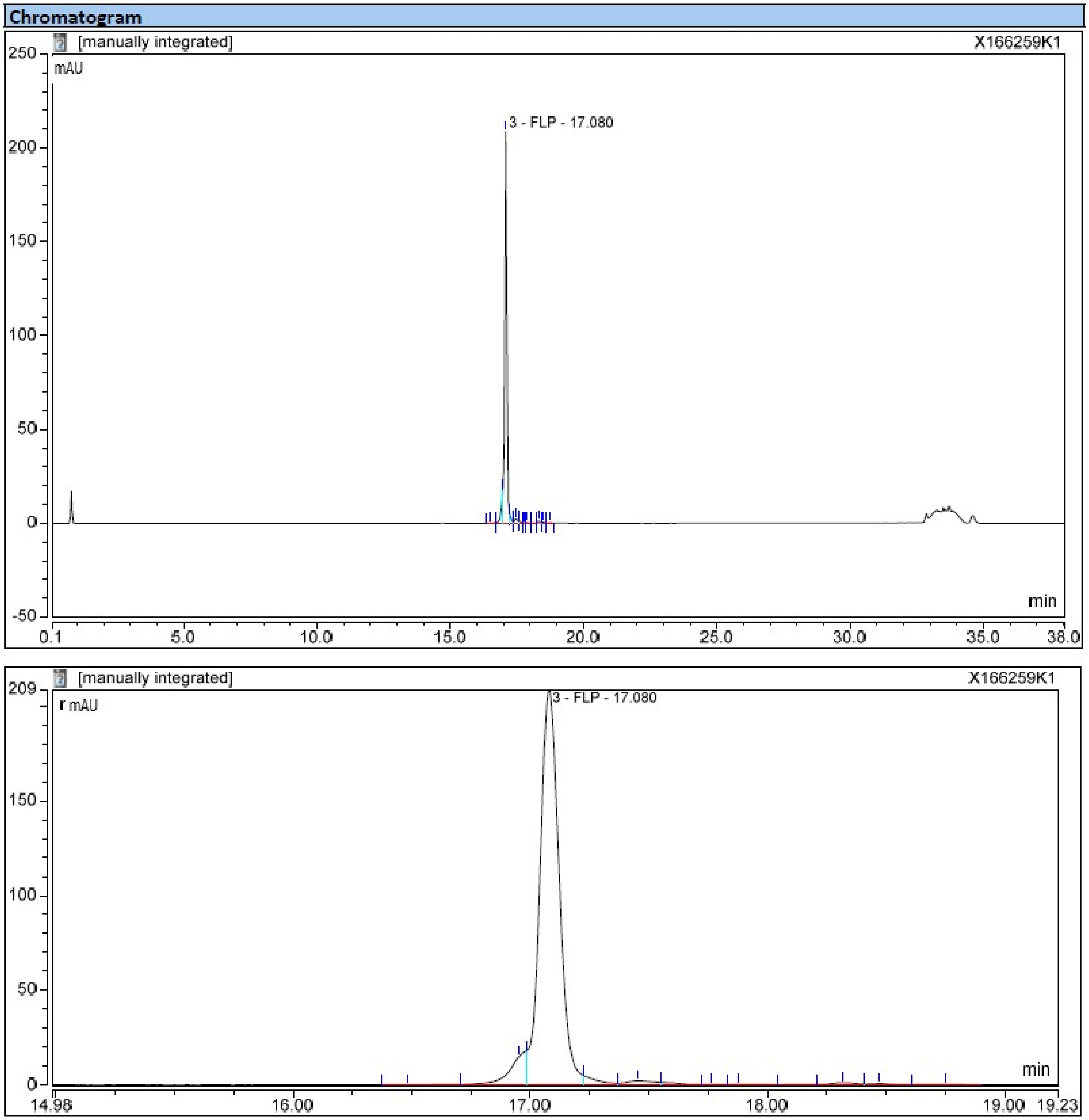

RP HPLC chromatogram for **ON14**

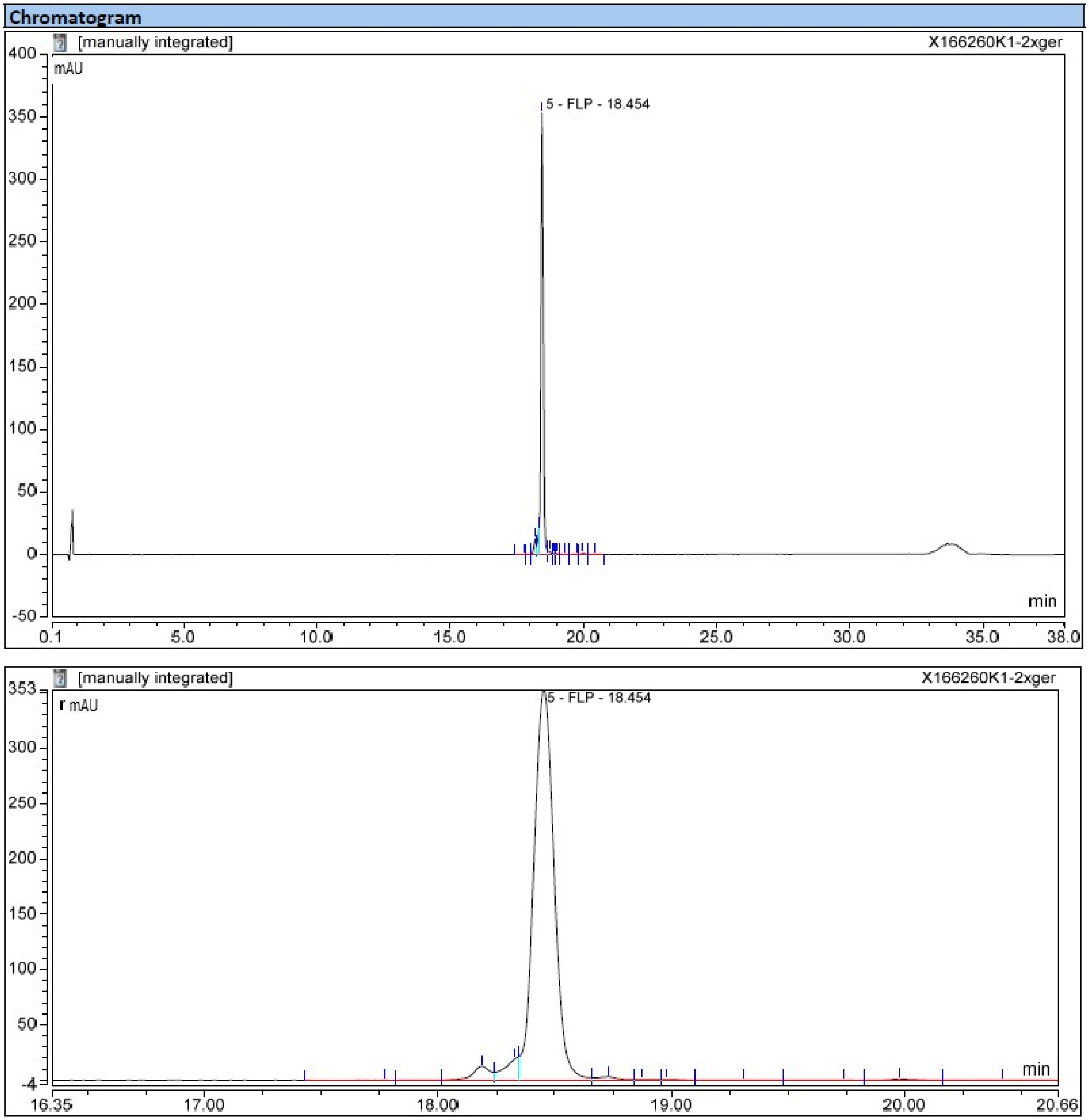

RP HPLC chromatogram for **ON15**

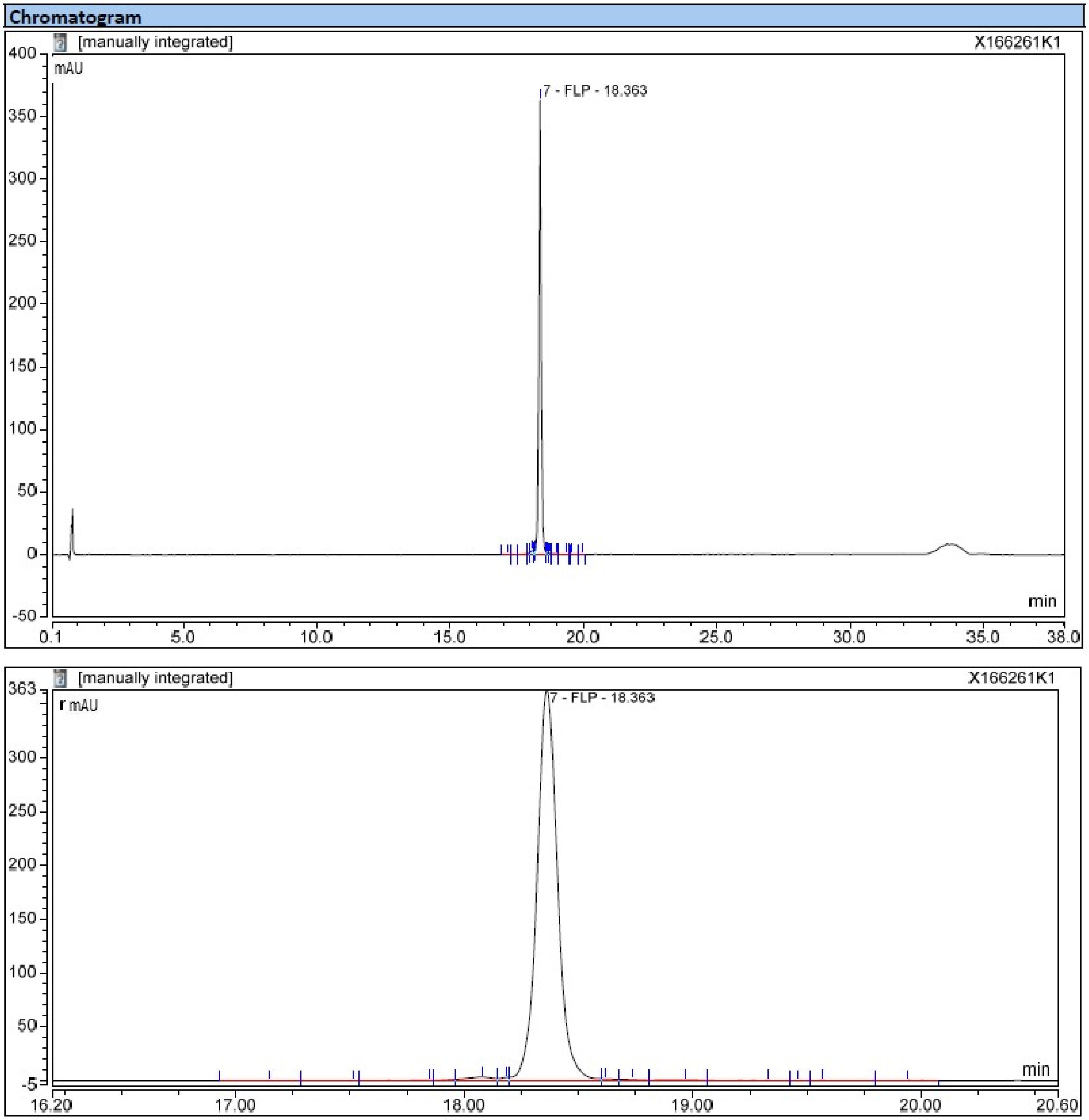

References

1. J. O'Shea, C. S. Theile, R. Das, I. R. Babu, K. Charisse, M. Manoharan, M. A. Maier and I. Zlatev, *Tetrahedron*, 2018, **74**, 6182-6186.
2. J. K. Nair, J. L. Willoughby, A. Chan, K. Charisse, M. R. Alam, Q. Wang, M. Hoekstra, P. Kandasamy, A. V. Kel'in, S. Milstein, N. Taneja, J. O'Shea, S. Shaikh, L. Zhang, R. J. van der Sluis, M. E. Jung, A. Akinc, R. Hutabarat, S. Kuchimanchi, K. Fitzgerald, T. Zimmermann, T. J. van Berkel, M. A. Maier, K. G. Rajeev and M. Manoharan, *J. Am. Chem. Soc.*, 2014, **136**, 16958-16961.
